# Observer Bias: An Interaction of Temperament Traits with Biases in the Semantic Perception of Lexical Material

**DOI:** 10.1371/journal.pone.0085677

**Published:** 2014-01-27

**Authors:** Ira Trofimova

**Affiliations:** Collective Intelligence Laboratory, Department of Psychiatry and Behavioral Neurosciences, McMaster University, Hamilton, Canada; University of Akron, United States of America

## Abstract

The lexical approach is a method in differential psychology that uses people's estimations of verbal descriptors of human behavior in order to derive the structure of human individuality. The validity of the assumptions of this method about the objectivity of people's estimations is rarely questioned. Meanwhile the social nature of language and the presence of emotionality biases in cognition are well-recognized in psychology. A question remains, however, as to whether such an emotionality-capacities bias is strong enough to affect semantic perception of verbal material. For the lexical approach to be valid as a method of scientific investigations, such biases should not exist in semantic perception of the verbal material that is used by this approach. This article reports on two studies investigating differences between groups contrasted by 12 temperament traits (i.e. by energetic and other capacities, as well as emotionality) in the semantic perception of very general verbal material. Both studies contrasted the groups by a variety of capacities: endurance, lability and emotionality separately in physical, social-verbal and mental aspects of activities. Hypotheses of “background emotionality” and a “projection through capacities” were supported. Non-evaluative criteria for categorization (related to complexity, organization, stability and probability of occurrence of objects) followed the polarity of evaluative criteria, and did not show independence from this polarity. Participants with stronger physical or social endurance gave significantly more positive ratings to a variety of concepts, and participants with faster physical tempo gave more positive ratings to timing-related concepts. The results suggest that people's estimations of lexical material related to human behavior have emotionality, language- and dynamical capacities-related biases and therefore are unreliable. This questions the validity of the lexical approach as a method for the objective study of stable individual differences.

## Introduction

### Language, emotionality and energetic capacities likely impose biases in perception of lexical material

#### Social bias of language

Language is an invention of group dynamics, which functions to facilitate socialization, an exchange of information and to synchronize group activity. The social function of language therefore serves the needs of the society more than the needs of an individual. This explains why there are several language-related biases in our lexicon. For example:

there are more behavioral descriptors of people in active states than in relaxed states;
there are more words related to social than physical aspects of behavior;
there are more words in our language describing negative emotions than positive emotions [1], and negative descriptors receive more attention [2]–[4]
there are more words describing energetic aspects of behavior than plasticity or tempo of actions.

Contrary to the bias of lexical descriptors in human language, healthy people are in a positive or balanced emotional state no less often than in a negative one, even when there are fewer words related to the positive states; people spend on average a minimum of half a day on either sleeping, napping, sitting while eating, driving, waiting, watching, or not engaging in intense activities. Unless we are talking about teenagers, people are involved in communication no more than in physical or intellectual activities. Furthermore, every action has a certain plasticity of construction and a tempo, i.e. not just the energetic, but also the lability aspects of actions are also present in every performance. These differences between the ratios in language descriptors and the ratios of actual occurrences of certain behaviors, demonstrate a language bias, compromising the validity of the public's evaluations which use lexical descriptors.

In addition to the social bias of language, people tend to conflate properties and traits that are similar in appearance, and this makes their opinions unscientific and unreliable [2], [4]–[8]. People's perception of “psychological types”, for example, are used by the lexical approach to derive a model of personality dimensions. In using this example, the conflation of the properties of an object of analysis (structure of human individuality) emerges when characteristics tend to either enhance or diminish each other's visibility when they play as an ensemble.

For example, high sociability (social endurance) is more noticeable in people with a high tempo of speech, or with high empathy, or with high impulsivity and adventure seeking. That does not mean that people with a low tempo of speech, or well-regulated people, or people avoiding risks cannot have high sociability. At the other extreme, well-regulated, intelligent and secure people, who have a low need for social approval and therefore low socialization, were thrown together by the lexical approach models into one (“introvert”) group with unempathic (psychopathic or autistic) people and/or people with low physical energy. Research into flat affect shows that patients with this symptom may be more affectively responsive than has usually been assumed. According to patients' reports as well as electrodermal measures of arousal, flat-affect patients actually have the same or even a higher affective response that do normal individuals [9]–[10]. It has been suggested that the discrepancy between the experience and expression of emotion (“affective incongruence”) may result from neuromuscular abnormalities that prevent normal (or even abnormally intense) emotions from being expressed in normal ways [11].

Anxious people were inferred to have a strong inhibitory system, and impulsive people were inferred to have a strong activation system. In clinical practice, however, anxious patients, especially children, show higher impulsivity and inability to focus than non anxious people. Another confusion is the attribution of impulsivity to highly active people with approach and sensation seeking tendencies, and of low-risk and low impulsivity to highly intellectual people. In reality, intellectual people are endowed with all kinds of “temperament packages”, including high or low sociability, with or without autistic tendencies, as well as with or without impulsivity, sensation seeking or high verbal-social tempo. Indeed in self-confident and/or energetic people, impulsivity is more visible when present, but depressed people, especially people with comorbid depression and anxiety, who report fatigue and slow-down also have impulsivity symptoms [12]. Such attribution errors are common, because a group of traits with similar behavioral appearances is more noticeable and can be attributed to one factor more readily than in cases where the same traits have “conflicting” directions.

#### Emotionality bias of cognition

Another factor that compromises the objectivity of people's evaluations: an impact of emotionality on cognition. Findings in neuropsychology showed that emotional evaluation always comes prior to the detailed cognitive assessment of the events, due to the lead of subcortical brain structures in information processing. Both subcortical and cortical structures are involved in emotional processing via multiple routes [13]–[16]. Vuilleumier and colleagues in their fMRI studies of the amygdala and related brain structures found a key role of these structures not only in emotional processing, but also in attention and perception [17]–[18]. Their results showed that emotional processes can modify perception – an effect that they called “emotional attention” [19]. Similar interlocking between emotionality and cognition was described be Pessoa [20]–[21]. Adolphs, Tranel and Buchanan [22] reported that enhanced memory for the visual details of a negative item within the affective-attentional network does not lead to the successful encoding of *all deta*ils of an item's presentation. Activity in these affective processing regions showed no correspondence (in the case of the amygdala) or a negative relation (in the case of the orbito-frontal cortex, striatum, and anterior cingulate gyrus) to the successful encoding of the task performed with an item. This means that even though emotional processing dominates the first stages of evaluation and creates emotionality bias, it misses important details of the perceived objects.

These neuropsychological findings, which showed how unconscious emotional processing can affect human cognition, were reflected in theories of emotionality, offering the concepts of “primary appraisals” [23], “basic emotions” [24]–[25], “core affects” [26], “somatic markers” [27] as pre-cognitive emotional dispositions [28]–[29]. This was in line with common observations from clinical psychology and psychiatry on biased perceptions of patients with mood disorders, based on the same neurochemical factors as temperament. The presence of a background emotionality seems to be a built-in primary biological component of semantic processing (facilitating approach-withdrawal behavioral reaction) not only in humans, but also in other animals, including the most primitive ones [30]. More recently direct studies of the impact of emotionality on the semantic perception of amodal lexical material reported a negative bias in the semantic perception of socially anxious and neurotic people [31]–[33].

With such a strong and direct impact on cognition, emotionality can collapse the structure of perceived phenomena into just two dimensions related to emotional valence. There are indeed two valence-based biases noted in neurophysiological studies of emotionality.

First, emotional arousal has a well-documented negativity bias [1]–[4] [34]–[36]. There are more cognitively distinct negative emotions than positive ones [37]–[38], and people appeared more likely to feel multiple positive emotions at one time than multiple negative ones [39]. A negative default reaction has been observed in human affective judgments [40]–[41], judgements of objects, events, or choices [42]–[43], and event-related brain potentials to affective stimuli [44]. Meta-analysis of autonomic activity in response to all negative emotions combined compared with all positive emotions combined also showed a negativity bias. The components of autonomic response (diastolic blood pressure, blood volume, cardiac output, left ventricular ejection time, pre-ejection period, pulse transit time, heart rate) had significantly greater activation during negative than positive emotions, and no autonomic responses showed the opposite pattern [44].

Second, socialization and perceived social support was found to have a positive emotionality bias. Mu-opioid receptors (MOP) inducing positive emotionality were found to be important players in the perception of social support [45] and in motivation by possible engagements in activities [46]. The impact of MOP and oxytocin on perceived social support and affiliative behavior was found to include dopamine (DA) release: both MOP and oxytocin induce this release during pro-social behavior. DA release in projections between NA, VTA and medial PFC also plays an important role in affiliative behaviour [24], [47], and an activation of the 5-HT2A receptor in hypothalamus reportedly increases hormonal levels of oxytocin, prolactin, ACTH, corticosterone, and rennin [48]. There are numerous reports on the role of oxytocin in perceived social support [47], [49]–[50], in affiliative behavior, and the formation of social memories [47]. Moreover, gonadal steroids, and vasopressin were found to have modulating effects on sensory, perceptual, and attentional processing of affiliative stimuli [49]. There are obvious evolutionary benefits for the development of a positive reinforcement system for socialization in humans, which we do not discuss here.

The bottom line is that these two regulatory systems – a system of emotional arousal and a system of affiliative behavior – are just two among many systems regulating human behavior. They, however, differ from other systems by having the power to induce a negative or positive emotional bias onto cognitive evaluations, due to the above noted interlocking between emotionality and cognition. As a result their biases will more likely appear in human estimations and even in the lexicon. The unconscious contribution of emotionality to cognitive processes raises concerns about the scientific validity of models that are based on people's evaluations, and not on experimental or clinical work.

#### Capacities-related bias in estimations of lexical material

Physical and social capacities were found to be interlocked with the way how people evaluate stimuli. For example, physical exercises were found to shift perception to more positive evaluations and even to endorphin-based improvement of depressive symptoms [51]–[54]. People with higher endurance in communication were reported to perceive neutral abstract concepts significantly more positively [31] and performed better on recall or categorization of emotionally positive words [32]–[33], than other temperament groups in these studies. A series of theories of embodiment, which echoed James-Lange theories emerged in cognitive psychology during the past 20 years [55]–[63].

Trofimova [31], in her study of the impact of sex and temperament on the perception of common words, described the phenomenon of «*projection through capacities*». Such a phenomenon emerges when a person's evaluations depend on their capacity to handle the events, and such evaluations therefore have bipolar outcomes (“can handle” or “cannot”) with corresponding positive or negative emotional dispositions towards the subject of evaluation. In projection through capacities, individual information processing therefore has a capacity-related bias, as the individual registers mostly those aspects of objects or of a situation that they can properly react to and deal with according to their inherent capacities (including a capacity to avoid harm). The interlocking of energetic capacities with emotional valence is in line with the idea of embodiment in cognition (supporting James-Lange theory) and the findings that the same neurochemical agents (neuropeptides and monoamine neurotransmitters) are involved in both energetic and emotional regulation of behavior. Such interlocking between energetic status and emotionality suggested that the largest dimensions in lexical approach models, i.e. Extraversion and Neuroticism, are not independent and essentially represent one dimension, and that executive and evaluative systems regulating human behavior might have a much closer interaction than was previously thought. This questions the benefits of a dimensionality-oriented approach in differential psychology, including the use of factor analysis, which relies on the independence of dimensions.

### Lexical approach models might not be applicable to investigations in biology of individuality, due to language, capacities and emotionality biases

The lexical approach in differential psychology involves people estimating very long lists of words for describing human traits and characteristics. A factor analysis is then applied in order to classify the traits into factors presented as dimensions of personality. The lexical approach was used by Cattell to derive his 16-factor model [64] and by a number of researchers proposing 5-factor models [64]–[71]. The modern 5-factor model was named the “Big Five” by Goldberg, and was intensively promoted around the world by McCrae and Costa [71].

Since phenomenon of personality is to a large extent a product of individual's socialization it would be natural to investigate how linguistic processes reflect the human perception of individual differences. This is a subject of investigation of cognitive psychology and, to a degree, personality psychology. The problem with the lexical approach arises when researchers manipulating the lists of verbal descriptors of personality present their models not as an interesting artifact of human verbal cognition or socialization processes, but as revealing a real core of biologically based individual differences in humans.

For example, researchers of the Big Five model claimed that it is capable of reflecting the structure of the biological systems of individuality (called either “first order” or “second order personality traits” within the lexical approach, and which are called “temperament”here), just by application of factor analysis to the lists of personality descriptors. (Note that temperament was a topic of research for over 100 last years, especially in European tradition in the work of Kant, Pavlov, Heymans, Wundt, Stern, Lasursky, Jung, Adler, Kretschmer, Spranger, Teplov, Nebylizyn, Eysenck, Thayer, Gray, Tellegen, Rusalov, Netter, Watson and Tellegen, Strelau, etc. Historically the North-American tradition of temperament research was scattered within three different disciplines: developmental psychology, psychiatry and personality theory looking for biologically based traits using a lexical approach. This might explain frequent misconception on North American psychology that “temperament is the same as personality”). In other words, the conclusions on the structure of biologically based traits were made using cognitive psychology methods.

The main assumption of the lexical approach is that correlations between common verbal descriptors of personality correspond to correlations between biologically based systems of human individuality. Only if this is true then the models of lexical approach can claim that they found a structure of the biologically based systems of individuality, and not just the nature of verbal descriptors themselves. To sort out whether or not the relationships between verbal descriptors can be considered as a representation of relationships within real biological systems of individuality we should look at: 1) nature of the descriptors (their source, scientific validation, possible biases), and 2) completeness of the list and any asymmetries in distribution of these descriptors that might lead to biases in resulting factors (keep in mind that the size of factors and the pattern of loadings are the direct reflection of such relationships).

1) In terms of the nature of lexical descriptors or their perception, the arguments above showed that they have at least three well-recognized types of biases: the socialization-bias of descriptors themselves, capacities-related (embodiment) bias and an emotionality bias in cognition. There are too many words related to socialization, in comparison to other words (socialization-language bias); there is a negativity bias in words related to emotionality; and there are more words related to energetic rather plasticity aspects of activities (capacities bias). Due to social and emotionality biases in our lexicon, if we collect all words related to individual differences and apply either factor analysis or just common perception in order to group these descriptors into conflating characteristics, the largest factors (groups) will be a) the words separated by the criteria of pro-social and socially-pragmatic values (Extraversion, or Activity are the candidate names) and b) the words contrasted by emotional valence, with a prevalence of negative emotionality, due to the prevalence of such words in human lexicon.

That is what happened to early temperament models and to the personality model of the lexical approach (often generalized as a general model of human individuality). Practically all early temperament models (starting from Kant's presentation of Hippocrates's four temperaments along the dimensions of Activity and Emotionality), which were based on people's observations (Kant, Wundt, Heymans, Adler, Kretschmer [72]–[75]), came up with two dimensions – those describing emotionality and energetic aspects of behavior. As language- and emotionality-biases are real and very strong, it is not a surprise that two factors of the Big Five model (Extraversion and Neuroticism) consistently appeared in the personality research performed in various cultures, and three other factors (Openness to Experience, Conscientiousness and Agreeableness) failed to show the same consistency across cultures or even within the same culture for different age groups [76]–[77]. The outcomes of the lexical approach in its contribution to differential psychology were, therefore, rather modest. Testing thousands just to confirm the relative independence of the two major dimensions of temperament would seem a rather expensive enterprise, especially since these two dimensions were already described in differential psychology for at least a century.

The emotionality bias in cognition implies that higher emotional people might have a greater bias and less ability to distinguish between non-evaluative criteria of assessment than lower emotional people. If so, there is a possibility that, if we rely on the estimations of verbal material by lower emotional people, it will diminish the emotionality bias (even though the language bias will remain). On the other hand, the estimations of lower emotional people might bring more contextual diversity, which would produce so much variance that it would result in too large a portion of variance unexplained by the resulting factors. The first possibility would improve the validity of the lexical approach, and the second possibility would not. The lexical approach does not differentiate between high-emotional and low-emotional people, but it is not clear if using it with low-emotional people would make any difference. Based on the reported emotionality bias in cognition, there are high chances that this bias affects the results of factor analysis, gluing criteria of estimations of highly emotional people in evaluative manner.

The existence of language- and emotionality biases in verbal descriptors of human behavior has serious implications for the use of the lexical approach in differential psychology. If these biases indeed exist then the models of lexical approach reflect only relationships between the lexical descriptors of human individuality, and not biologically based systems of this individuality. For this reason science usually does not rely on inexpert evaluations of words describing natural phenomena, but experiments and natural observations of these phenomena are used instead, especially when it comes to claims in regards to biology and physiology.

This paper investigates if and how these biases show up in different temperament groups, but before we focus on the details of the study, let us briefly comment here on the second key flaw in the clam of the lexical approach related to a discovery of biological systems of individuality.

2) As noted, in order for lexical approach models to reflect the nature of phenomena under study (biological systems of individuality) their lists of descriptors should be complete and should arise within the scientific, not the general, community. Eschewing experimental and physiological techniques for investigation of what biological regulatory systems indeed exist, lexical approach researchers also insist that their concept of personality is practically equal to the concept of temperament. In American psychology every time that a study on temperament is performed, it is common for reviewers to demand the inclusion of references to findings from some lexical approach study.

Meanwhile temperament is defined as the most consistent, biologically based dynamical aspects of behavioral regulation, which are relatively independent from educational and socio-cultural impact [78]–[84]. Similarly to other factors which depend on neurochemistry and neurophysiology of the body (such as sex and age), temperament should be treated as comprising the biological systems of human individuality, and not personality. Similarly to the interaction of sex- and age with social factors leading to specific personality peculiarities, temperament also interacts with socialization factors; it is a different, more basic and older concept than personality, and has its own research field. There is more than a century of findings from experimental studies on the structure of temperament, clinical work in differential psychology or studies in neuropsychology by Pavlov, Spranger, Stern, Lasursky, Jung, Teplov, Nebylizyn, Eysenck, Rusalov, Strelau, Cloninger, Robbins and Everitt, Halgren and Marinkovic and many prominent neuropsychologists ([78]–[93] – see the review of temperament models and references in [28]–[29], [83], [94]).

On this criterion of completeness the lexical approach also shows significant flaws: it completely misses the regulatory characteristics related to lability of behaviour (plasticity-rigidity, tempo of verbal or physical activities). Yet, these characteristics are well-known for being based on biological systems. Pathologically low plasticity (perseverance) has been described in clinical cases of frontal lobe damage for over 70 years. Contrary to unification of endurance and tempo under one dimension of Extraversion in the Big Five model, these characteristics are based on different biological systems: just consider the difference between capacities used by marathoners and sprinters.

More specifically, it was suggested that temperament appeared to regulate three dynamical aspects of activities (energetic, programming-lability and sensitivity-orientational), multiplied by two probabilistic levels of regulation (stereotypical, learned vs. new or complex activities). There also are different traits regulating three distinct functional aspects activities of activities (motor vs. social vs. mental) and there are, of course, at least three types of emotionality traits (neuroticism, confidence and impulsivity), which do not substitute for energetic or plasticity components of behavioral regulation [28]–[29], [83], [94], [95]. These dynamic properties, described by the concept of temperament, traditionally were considered separately from content-related aspects of behaviour, such as values, beliefs, knowledge, motives, preferences, i.e. the key components of personality. After all, content-related aspects were thought to be mainly based on socio-cultural factors, and temperament is based on biological factors. Ever since it was described by the physicians Hippocrates and Galen 2500 year ago, European researchers of temperament linked it to the properties of nervous systems, neurochemistry and endocrinal regulation of the body.

This work showed much higher, nonlinear, dimensionality and complexity in biological regulation of behavior, even though these models also included emotionality and energetic dimensions. After all it is hard to believe that complex human behavior would be regulated just by two biological systems. Robbins and Everitt [92] pointed out in regards to the concept of Extraversion that “*various indices of arousal do not intercorrelate to a high degree, as would be expected of a unitary construct (Eysenck, 1995), and putative manipulations of arousal, whether pharmacological or psychological, do not interact in a manner suggestive of an underlying unidimensional continuum*” (p.703). Instead they described not one, but rather four reticular arousal systems, related to four types of neurotransmitters.

There have been practically no studies (except the cited three [31]–[33]), which directly investigated all named biases in the semantic perception of verbal material. One of the main reasons for this is the methodological challenges involved in studies perception of the meaning, i.e. semantic perception.

### Methodological considerations of studies investigating the semantic perception of lexical material

The main challenge in studying the semantic perception of words is the diversity of meanings and associations that people attribute to the words. Meaning appeared to be individually unique and different not only between people from different cultures, social and family background, but also between all individuals. Many parametric methods appeared to be useless in measuring meaning, as what psychomantics does looks like comparing oranges and apples.

The first success in identifying commonalities in semantic perception came with Kelly's [96] theory of personal constructs. This theory suggested that people assess events and objects using criteria (“constructs”) of a bipolar nature. Subsequently, a branch of cognitive psychology, psychosemantics, was established and flourished after Osgood [97] demonstrated the existence of at least three universal dimensions in semantic perception in people from 24 cultures. Osgood named the three most commonly found factors “Evaluation” (which included, for example, the scales “pleasant-irritating”, “clear-dirty,” “kind-cruel”), “Activity” (“energetic-constrained”, “monotonous-keen”, “fast-slow”) and “Potency” or “Power” (“strong-weak”, “firm-flimsy”, “massive-miniature”). Eventually it was noticed that Osgood's factors present predominantly evaluative (emotional) criteria for the assessment of objects.

The evaluative nature of Osgood's three dimensions of semantic perception was often presented as a criticism of Osgood's findings, but what is important in the context of this paper is that the scales had a tendency to glue together into factors in a rather consistent manner, regardless of the objects of estimation. The fact that the scales' grouping was not related to the specifics of objects, was bad news for the lexical approach. This meant that people, as observers, had some intrinsic structures in their semantic space that could impose consistent biases on their estimations.

At the same time, studies of human and animal cognition showed that our perceptual processing is organized not only around assessing the valence of objects or events, but also around probabilistic features. Subsequent studies in psychosemantics found a few additional universal dimensions: “Typicality”, or “Probability” (“typical-exclusive”, “regular-rare”) [31], [98], “Improvement”, or “Organization” (“organized–non-organized”, “regular-spasmodic” “constant-changeable”, “precise-indefinite”), “Reality” (“imaginary-real”, “evident-fantastic”, “abstract-concrete”), “Complexity” (“complex-simple”, “mysterious-usual”, “unlimited-limited”), Stimulation (“interesting-boring”, “trivial-new”) [31], [99]–[100], and other small factors. These findings suggested that people might be using non-evaluative criteria (complexity, organization, stability and probability of occurrence) and evaluative, emotion-based (“good-bad”, “exciting-boring”) criteria independently. Such independence of non-evaluative criteria in semantic perception would validate the lexical approach method.

A common way to extract biases in meaning attribution is to apply projective methods, using material of a very general nature, which is open to individual interpretation. Projective Semantics [31], [101] based on Osgood's [97] Semantic Differential method (SD), asks people to estimate well-known general concepts using common adjectives in the form of bipolar scales. These scales are then grouped into a small number of factors to facilitate the analysis. The difference between the Semantic Differential and the Projective Semantic methods is that the SD studies conducted by Osgood or his followers used various numbers (between 15 and thousands) of diverse adjectives and concepts to be assessed. The Projective Semantic method uses primarily concepts of a very specific nature: these nouns correspond to the 7 groups (factors) of adjective-scales, which were those most consistently found in cross-cultural studies, as described in the previous paragraphs. Seven types of adjective scales and seven types of concepts describing the same aspects of reality as the scales establish the *object-scale symmetry* (OSS) between scales and objects, which improves the sensitivity of the method to any bias in responses.

For example, if there are no differences between participants in the perception of the common words, then the concepts “Reality”, “Present” are expected to be assessed unequivocally as “very real” on adjectives such as “real-imagined” (i.e. scales of the Reality factor); the concepts “Complexity”, “Chaos” as “very complex” along the scales of the Complexity factor, the concepts “Beauty”, “Freedom” are expected to be on the positive pole on the Evaluation scales, “Time”, “Development” – on the negative pole of the scales of the Stability factor, etc. It is expected that a deformation of this symmetric and very basic matrix would reveal underlying biases of two types: in either using certain scales and/or in the assessment of certain concepts. The concepts are chosen to correspond not only to the groups (factors) of scales, but also to temperament traits, i.e. to the dynamical characteristics of behaviour (Effort, Work, Relaxation – to scales measuring endurance; Speed, Motion – to scales related to Tempo and Plasticity of activity; Prestige, Reputation, Beauty – to Emotionality scales) and to the scales related to different areas of activity, such as physical (Motion, Work, Speed), social (Society, Person, My contemporary) and intellectual (Complexity, Chaos, Time, History).

Before we summarize the hypothesis and methods, the following requirements should be mentioned, which narrowed the focus of our study:

- *A concern about an impact of culture and education* on the semantic perception of lexical material. Since Luria's experiments in the 1930s within low-educated cultures it was shown that culture is an important determinant of which kinds of non-evaluative criteria people use for classification and evaluation (See [102] for review). One component of the educational impact is a previous functional experience related to the objects of assessment. We agree with Cree and McRae's [103] differentiation between amodal semantics, sensory/functional semantics and domain-specific semantics (which classifies objects according to concrete, knowledge-based features). The assumptions of the lexical approach relate to amodal semantic perception as this approach was applied to the concepts of personality and human character, and searched for cross-cultural universality. Our study therefore should, similar to the lexical approach, focus on amodal (and not so much on sensory/functional or feature-specific) semantic perception using the most common lexical material, accessible for all cultures and educational levels.
- *A concern about a situational embodiment effect.* The idea that the somato-visceral feedback from the body affects the emotional perception of an individual was suggested independently by James and Lange at the end of the 19^th^ century. It was echoed in Bernstein's work on action construction [104]–[105], who concluded that the initial stage of our attribution of meaning to objects is related to a body-object interaction (p. 126). Several constructivist theories suggested that evaluative and motor systems of behaviour do not function in parallel, but rather work in an ensemble, simultaneously constructing an action every time anew based on the state, need and resources of the body [27], [31], [55], [104]–[108]. The embodiment theories of cognition suggested that neurophysiological systems involved in physical action contribute to the representation and comprehension of language stimuli [56]–[63], [109]–[113]. Fast implicit motor activation during language processing was found to be an important component of disposition for semantic retrievals [59]–[63]. Moreover, it was shown that the brain appears to process meaning attribution to words in a *modality-specific* manner when it comes to concrete activities and objects [61], [109]–[114]. To control these effects, in addition to the application of amodal lexical material, a setting up of physically identical conditions for all subjects would help to minimize a possible situational embodiment-related bias (i.e. bias based on the specifics of body-object interaction in a given moment). The difference between the majority of embodiment studies using tasks of specific modalities and this study is that the experimental groups of the former were contrasted by more diffuse (unrelated to specific actions) dynamical aspects of behavioral regulation: endurance, changeability, directionality of behavior. In contrast to the well-defined anatomy of motor and sensory systems, temperament is based on the neurochemistry of the body (neurotransmitters, neuropeptides, opioid receptors and hormones), and therefore this study presents a different aspect of embodiment research.

### Goals, hypothesis and methodological considerations of the present studies

One of the assumptions behind the lexical approach, which this study challenged, is that existing relations between features of human behavior will be adequately reflected in correlations between lexical descriptors of behavior without any language or emotionality bias. Therefore, one of the goals of our study was to investigate whether temperament groups, which are contrasted by the energetic and sensory capacities perceive lexical material differently. More generally, it was investigated if semantic perception of lexical material differed between these contrast groups for verbal non-evaluative criteria of organization, probability, complexity and stability. The first, the *projection through capacities* hypothesis, was based on embodiment theories and on reports describing the interlocking between capacities and emotionality, and between emotionality and cognition. According to this hypothesis people with higher endurance or tempo in either physical or social aspects of behaviour perceive objects, even amodal verbal material, in more positive terms in comparison to their opposite temperament groups.

It is not at all clear, however, whether people with higher social-verbal endurance and tempo of speech have more adequate semantic perception of lexical material than people with higher intellectual or physical endurance. These people might either have a more detailed perception of social concepts, or, alternatively, the social nature of language might create a positive bias in their perception in favor of socialization-related concepts. Mental (intellectual endurance) is expressed in individual attentive abilities. It is possible that people with higher scores on this trait might have more adequate use of criteria in the assessment of abstract concepts.

Moreover, the logic of embodiment theory suggests that lability traits of temperament (ability to restructure actions and tempo of performance) might have an impact on the perception of time-related lexical constructs. To date there has been practically no research focusing on the semantic perception of time concepts by people with different speeds of performance. The only study with such focus was conducted by Trofimova [31], which used a small number of scales and concepts. This study reported that participants with higher physical tempo perceived abstract concepts as more fast, more energetic and acute whereas participants with higher verbal tempo perceived the same object as more constant. As an indirect indication of the body's-tempo-related bias in the perception of action verbs, several studies showed a presence of motor activation during the processing of these verbs [112]–[113], [115]–[116]. Based on these results we hypothesized that people with a stronger dynamical (temperamental) system generating a *tempo* of performance will project more energetic and speedy properties to abstract concepts than people with slower tempo.

Our second goal of the study was related to the assessment of a possible impact of language (socialization) bias, especially in the perception of lexical material by people with high social-verbal capacities. The second, *language bias* hypothesis, suggested that people with stronger endurance and tempo in verbal-social activities would differ from people with stronger motor-physical or intellectual endurance in their semantic perception of lexical material. Moreover, as a part of this hypothesis we investigated if temperament traits which are not related to socialization or emotionality (such as plasticity and tempo) will have any interaction with semantic perception of lexical material.

The third goal of this study was to investigate if there is a conflating impact of emotionality bias in the use of non-emotional criteria of estimations. If there is no such emotionality bias, then the non-evaluative (probabilistic or structural) criteria would be used independently from evaluative criteria in semantic processing. If there is such emotionality bias, the polarity of non-evaluative criteria would be conflated with the valence of evaluative criteria. Our *emotionality bias* hypothesis suggests that emotional dispositions create a universal, object-independent bias in meaning attribution, and that this bias is especially strong in amodal semantic perception (i.e. when no concrete feature-specific objects are given, and a person has to assess abstract lexical material). We hypothesize that this bias might be so strong that it forces non-evaluative criteria to follow the valence of emotional evaluative criteria. In this case the background emotional component of semantic perception would correlate (or “glue”) parameters of assessment into the factors regardless of the objective features of these elements.

At this point we have three hypotheses, which relate to capacities-, language- and emotionality biases in semantic perception. In order to separate these biases in our investigation we had to use experimental groups that were contrasted by various aspects of physical, social and intellectual capacities and emotionality. Temperament traits are hard to monitor in short-term experiments, as they emerge only as consistent behavioral patterns in a variety of situations over long periods of time. Such monitoring is especially challenging if we need to study all traits at once. Neurophysiological methods for separating contrast temperament groups are also not developed yet, as temperament traits are based more on neurochemical processes rather than brain morphology, visible in neuroimagery studies. For these reasons the main instruments to diagnose temperament are validated temperament tests.

The present study used the activity-specific tests of temperament, which make the most detailed differentiation between all main aspects of temperament (i.e. biologically based systems regulating behavior). Both tests were developed within the longest-running (Pavlovian) experimental tradition investigating properties and types of nervous systems. These tests are called “activity-specific” because they differentiate between temperament traits regulating physical, verbal-social and mental aspects of actions [39], [94]–[95], [117]–[120]. Both tests consists of 12 temperament traits: 3 traits of Emotionality and 9 executive traits (related to energetic, lability and orientation aspects of actions, each considered in 3 types of activity – physical, social and mental). Such an integrated approach (admittedly complicating the readability of the results) was necessary to analyze for the possibility of interactions between the three described biases in the perception of the participants of the study.

In sum, to test our hypothesis we used 12 independent variables, corresponding to 12 temperament traits. Dependent variables were not single measures, but patterns of relationships between multiple semantic measures (scales). The analysis was focused on both quantitative (statistically significant differences) and qualitative effects (the way that these differences were grouped along the poles and features of the scales). We expected the following:

1. If the “*projection through capacities*” hypothesis is true, the results would show: a) that people with higher endurance attribute more positive meaning to neutral common words than people with low endurance; b) that people with higher tempo would have universally higher estimations to timing-related concepts (Time, Motion, Development, Speed) than other temperament groups.
2. If the “language bias” hypothesis is true, a temperament group with higher verbal-social endurance will give more positive estimations of concepts than other temperament groups, due to their better verbal capacities;
3. If the “*emotionality bias*” hypothesis is true, then a) the differences in estimations on non-evaluative scales (describing probability, complexity and organization aspects of concepts) would follow the same polarity as on evaluative scales (which belong to Evaluation or Stimulation factors); b) emotional reactivity, as an expectation of a failure, will affect the estimations of people with higher emotionality or neuroticism, emerging as a universal negative evaluative bias in their estimations.

## Method, Study 1

### Sample

846 undergraduate psychology students of McMaster University and Brock University (Southern Ontario) (85% of the sample) and volunteers from the same area aged 17–52, M/F  = 331/545 participated in this study in 2000–2003. All participants were fluent in English. After validity procedures were applied the final sample included 312 men, *M_age_ ± SD*  = 21.9±7.4 and 506 women *M_age_ ± SD*  = 20.5±5.4.

### Procedure

The study received an approval from the McMaster University Ethics Committee for all procedures. All subjects received debriefing and signed an informed consent form and then completed the Extended Structure of Temperament Questionnaire and participated in the experiment. University students received a practicum credit for their participation.

The Extended Structure of Temperament Questionnaire (STQ-150) [83], [95], [117]–[118] has 150 statements to be answered with a Likert scale format: “strongly disagree (1),” “disagree (2),” “agree (3),” “strongly agree (4)”. Six items are assigned to the validity scale, and 144 items to 12 temperament scales (12 items each). (A brief summary of the validation history of the Extended and Compact STQ is given in Text S1). The scales are as follows (alpha values are given in parenthesis based on the data in this study):

1–3. Scales of Motor, Social and Intellectual Endurance (“Ergonicity”) (ERM, ERS, ERI), assessing the ability of an individual to sustain prolonged physical (alpha = 0.83), social (0.83) or mental (0.73) activity respectively.

4–6. Scales of Motor, Social and Intellectual Tempo (TMM, TMS, TMI) assessing the speed of manipulations with objects (0.72), tempo of verbal activity (such as talking and reading) (0.75) and tempo of performing intellectual tasks (0.70) respectively.

7–9. The scales of Motor, Social and Intellectual Plasticity (PLM, PLS, PLI) assessing the ability to adapt quickly to changes in instructions or circumstances in object-related operations (0.71), communication (0.77) or intellectual activity (0.71) respectively.

10–12. The scales of Motor, Social and Intellectual Emotionality (EMM, EMS, EMI) – assessing sensitivity to failure and to success in physical (0.74), social (0.71) and mental (0.75) activities respectively.

Protocols having scores of 18–24 on the Validity scale were considered invalid as the respondents were likely to demonstrate a positive impression bias in their responses.

The Semantic Task experiment used 60 6-point bipolar scales to estimate the 29 general concepts (Table 1) chosen according to the Projective Semantic method as described above. Each concept was presented by the program “Expan” on a computer monitor at the top of the screen along with each of the bipolar evaluating scales placed horizontally at the middle of the screen (i.e. 1740 screens were presented for the estimation). Both poles of the scales had 3 degrees of freedom (“very much”, “somewhat”, “weakly”). The order of scales and concepts was changed for each protocol to avoid the consecutive use of several scales related to one factor.

**Table 1:** The list of scales (grouped into 7 factors) and concepts used in the Study 1.

*Scales:*	*Scales:*	*Concepts:*
+ pole − pole	+ pole − pole	
**“Stimulation”**	***complex-simple***	Activity
original – trivial	***continuous-discrete***	Future
exciting-indifferent	***inexplicable-understandable***	Faith
bright – pale	***difficult - easy***	Time
stimulating-draining	**“Reality/Probability”**	Society
interesting-uninteresting	natural-artificial	Movement
unusual-ordinary	true-false	Life
arouses – calms	existent-imagined	Unknown person
sharp – dull	real – imaginary	Task
**“Evaluation”**	possible-impossible	History
pleasant-irritating	known-unknown	Beauty
kind – severe	inevitable-improbable	Present
progress-decline	typical-atypical	Relaxation
light – dark	common-rare	Order
pure(clean)-dirty	**“Organization”**	Prestige
good – bad	*clear – blurred*	Past
warm – cold	*regular – irregular*	My contemporary
useful – harmful	*rational – irrational*	Work
mine – not mine	*justified – senseless*	Development
soft – rigid	*obvious – obscure*	Reality
**“Power”**	*reliable – unreliable*	Reputation
**massive-delicate**	*precise – imprecise*	Freedom
**rough – smooth**	*organized-unorganized*	Power
**large – small**	*assembled-unassembled*	Speed
**deep-superficial**	*planned- spontaneous*	Complexity
**resonant – tinny**	**“Stability/Limits”**	Simplicity
**powerful – weak**	stable – unstable	Effort
**leading-following**	steady – faltering	Chaos
**significant-insignificant**	Constant– changeable	Person
**“Complexity”**	fixed – flowing	
***multi-dimensional – one-dimensional***	restrained-unrestrained	
***chaotic – ordered***	limited – boundless	
***diverse – uniform***	finite – infinite	
***irreplaceable- replaceable***	solid – fragile	

### Statistical processing

To avoid the impact of a social desirability or negative impression bias, the contrast temperament groups were selected, not based on the T-scores on the STQ, but based on the relative rank of the position of a score on a given scale in relation to scores on the other scales within an individual temperament's profile. The implication was that participants might over/underestimate the expression of their dynamical traits under the influence of social expectations, but they could be more objective about which temperament characteristic is stronger or weaker in comparison to their other characteristics. For each protocol, the scores on 12 scales were transformed into 12 ranks. The protocols were sorted into three groups with lowest, middle and highest ranks and the contrast (i.e. lowest (1–4) and highest (9–12)) groups were processed further. Table 2 shows the sizes of the contrast groups, and Table 3 shows the statistical details related to temperament scores. The differences in estimations were assessed with the Mann-Whitney U test, separately for men's and women's contrast temperament groups (based on 12 scales of the STQ-150). To control multiple comparisons using the Bonferroni correction the significance of differences was set to *p*<0.0063.

**Table 2:** Sizes of the temperament contrast groups.

Study 1	Men	Women		Study 2	Men	Women
*STQ-150 scales*	w/s	w/s		*STQ-77 scales*	w/s	w/s
Motor Endurance,ERM	92/141		182/157	Motor Endurance	44/51	91/71
Intellectual Endurance, ERI	110/92		169/166	Intellectual Endurance, ERI	52/40	93/55
Social Endurance, ERS	93/125		125/155	Social Endurance,ERS	44/45	45/90
Motor Plasticity, PLM	77/100		151/138	Plasticity, PL	39/31	52/62
Intellectual Plasticity	90/84		139/133	Sensitivity to probabilities, PRO	36/50	78/63
Social Plasticity, PLS	74/106		164/162	Empathy, EMP	30/51	58/71
Motor Tempo, TMM	80/74		150/166	Motor Tempo, TMM	49/51	81/60
Intellectual Tempo, TMI	63/103		149/132	Sensitivity to sensations, SS	42/46	62/67
Social Tempo, TMS	101/103		91/149	Social Tempo, TMS	51/36	54/70
Motor Emotion-ty, EMM	111/78		130/140	Self- confidence, SLF	33/40	71/75
Intellectual Emotion-ty	91/95		157/195	Impulsivity, IMP	58/33	73/63
Social Emotion-ty, EMS	123/96		140/195	Neuroticism, NEU	43/49	59/88

*Note:* w/s means the number of individuals with **w**eaker/**s**tronger expression of a given trait. Three degrees of the strength of a trait (weak, medium and strong) were derived from the ranking of the 12 scores in individual temperament profiles (4 traits per degree).

**Table 3:** Means (*M*), and standard deviations (*SD*) and ANOVA effects (*F, p*) of sex differences in means on STQ-150 scales (Study 1).

	Men,	N = 312	Women	N = 506	*F*	*p*
*STQ-150 scales*	*M*	*SD*	*M*	*SD*	*(1, 816)*	
Motor Endurance, ERM	33.56	7.87	30.29	7.12	37.61	0.00
Intellectual Endurance, ERI	30.81	5.73	30.18	5.47	2.46	0.12
Social Endurance, ERS	33.40	7.21	35.47	6.82	17.10	0.00
Motor Plasticity, PLM	31.85	5.65	30.43	5.44	12.72	0.00
Intellectual Plasticity, PLI	31.38	4.88	29.01	5.03	43.91	0.00
Social Plasticity, PLS	29.72	6.05	28.98	6.67	2.56	0.11
Motor Tempo, TMM	34.10	6.04	32.56	5.02	15.65	0.00
Intellectual Tempo, TMI	34.14	5.47	31.80	5.36	36.37	0.00
Social Tempo, TMS	32.81	5.75	36.17	5.77	65.77	0.00
Motor Emotionality, EMM	27.84	5.46	26.04	6.04	18.28	0.00
Intellectual Emotionality	29.08	5.28	30.94	6.05	20.06	0.00
Social Emotionality, EMS	27.93	6.02	30.09	5.68	26.79	0.00

## Results, Study 1

**Figure 1:**
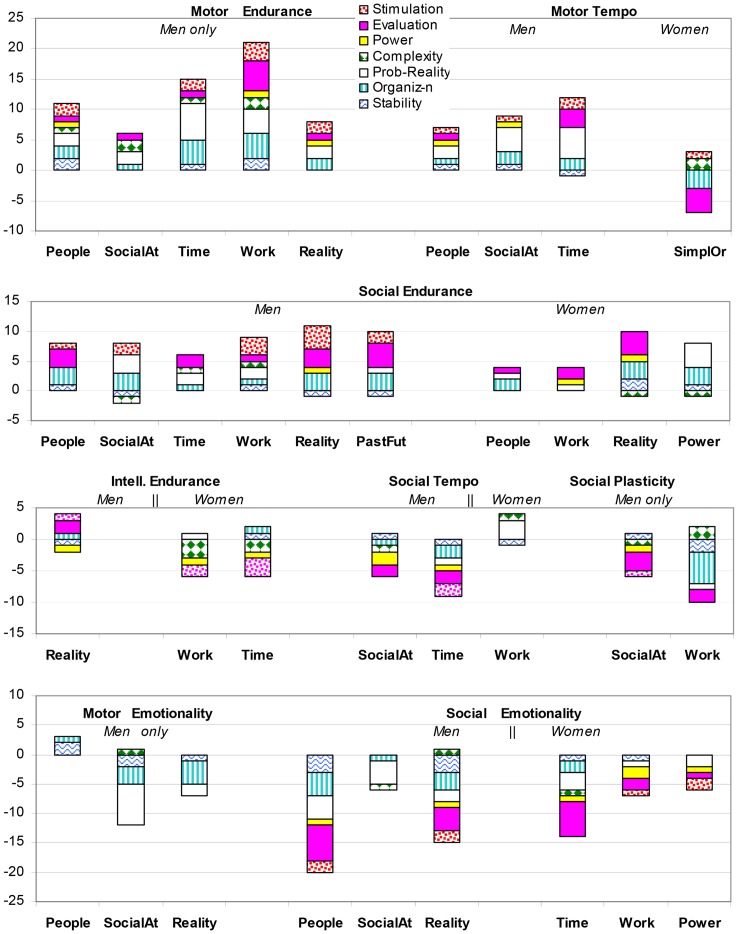
The number of statistically significant differences in estimations of contrast temperament groups, Study 1. Axis Y: the total number of significant differences; the sign indicates the pole of the scales chosen by the group with the higher scores on a given temperament trait. The colours represent the spectrum of these differences along seven factors to which the scales are associated. Axis X: groups of concepts (see text for details).

In order to summarize the results of 1740 estimations given by each participant in 24 contrast temperament groups and 2 gender groups, we grouped the objects according their cluster analysis and grouped the scales according to factor analysis. Factor analysis confirmed the affiliation of the scales to seven factors, as presented in Table 1: Stimulation, Evaluation, Power, Reality-Probability (typicality), Organization, and Stability-limitation. Figure 1 shows the number of statistically significant (at *p*<0.0063) differences in estimations in the following groups of concepts: “People”(Person, Unknown person, My contemporary, Society), “SocialAt” (social attractors: (Prestige, Reputation, Beauty, Freedom), “Reality” (Reality, Present, Life), “Work” (Work, Task, Activity, Effort), “Time” (Time, Speed, Motion, Development), and “PastFut” (Past, Future). Other concepts did not show noticeable significant differences in contrast temperament groups, except 2 cases: “Power” in the male Social Tempo contrast group, and “Simplicity”, “Order” in the female group contrasted by Motor Tempo (marked as SimpOr in the Figure 1).

In more detail, Figure 1 assigns a specific color pattern to each of 7 factors (connotative groups), to facilitate the perception of the spectrum of significant differences. The height of the pattern is proportional to the number of scales related to the particular factor that showed significant differences, with greater heights representing more scales (see Table 4, Table S1 for statistical details). The position of the patterns with respect to the zero line indicates the polarity of estimations preferred by the group with the higher scores on a given trait, i.e. to which side of the scale this group's means were closer. The above 0 position indicates that means were closer to the positive pole of the scale; below 0– to the negative pole, see Table 1 for the assignment of poles. For example, for the concepts of social attractors, Figure 1, Table 4 (and Table S1) shows that in total 10 scales had significant differences in the male group contrasted by Social-verbal Endurance trait. From the height of each pattern one deduces that there were significant differences on the scales of the Stimulation (2 scales), Probability (2 scales), Organization (3 scales), Stability (1 scale) and Complexity (1 scale) factors. This stacked column shows that the estimations of socially energetic men are significantly closer to the positive poles on Stimulation, Probability and Organization descriptors and to the negative pole on Stability and Complexity adjectives than those given by lower energetic men. Similarly, for the same concepts and the same temperament group (high Social Endurance), but in the female sub-sample, there were 9 scales, with the height of the patterns indicating significant differences on 4 Probability scales, 3 Organization scales, and also 1 Stability and 1 Complexity scale, with highly social women preferring the positive poles of the eight scales.

**Table 4:** Selected list of the significant differences in estimations of contrast temperament groups, Study 1.

MEN with	Z	*p*-level		MEN with	Z	*p*-level	
WEAKER	Motor	Endur.	STRONGER	SLOWER	Motor	Tempo	FASTER
estimated		**People**	AS MORE:	estimated	'**Soc**	**At**'	AS MORE:
draining	3.98	.0001	stimulat-g	draining	2.84	.0046	stimulat-g
uninterg	3.06	.0022	interesting	**weak**	2.89	.0039	**powerful**
decline	2.79	.0053	progress	artificial	2.71	.0067	natural
**weak**	**3.68**	**.0002**	**powerful**	false	3.43	.0006	true
***one-dimen***	***2.80***	**.** ***0051***	***multi-dim.***	impossible	3.78	.0002	possible
artificial	4.18	.0000	natural	inaccessib	3.54	.0000	accessible
imaginary	2.94	.0033	real	*irrational*	4.54	.0000	*rational*
*irregular*	*2.87*	.*0041*	*regular*	*unreliable*	4.07	.0000	*reliable*
*imprecise*	*3.79*	.*0001*	*precise*	faltering	3.41	.0007	steady
faltering	3.18	.0015	steady	WEAKER	Social	Endur.	STRONGER
fragile	3.98	.0001	solid	uninter-g	2.67	.0068	interesting
LOWER	Social	Emot.	HIGHER	abundant	3.33	.0009	popular
stimulat-g	3.13	.0017	draining	***difficult***	***3.10***	**.** ***0019***	***easy***
interesting	2.79	.0053	uninter-ng	artificial	3.04	.0024	natural
pleasant	3.36	.0008	irritating	impossible	3.15	.0016	possible
kind	3.30	.0010	severe	unusual	3.22	.0013	ordinary
progress	4.50	.0000	decline	*irregular*	2.84	.0045	*regular*
light	2.97	.0030	dark	*irrational*	3.42	.0006	*rational*
pure	3.26	.0011	dirty	passive	3.08	.0020	active
warm	2.81	.0050	cold	LOWER	Motor	Emot.	HIGHER
**smooth**	**2.83**	**.0047**	**rough**	***understand***	***3.80***	**.** ***0001***	***inexplicable***
natural	3.40	.0007	artificial	true	2.80	.0052	false
true	3.76	.0002	false	existent	2.99	.0028	imagined
known	2.86	.0042	unknown	real	3.91	.0001	imaginary
inevitable	3.61	.0003	improbab.	possible	3.66	.0003	impossible
*clear*	*3.91*	.*0001*	*blurred*	known	3.71	.0002	unknown
*regular*	*3.93*	.*0001*	*irregular*	inevitable	3.82	.0001	improbable
*rational*	*3.16*	.*0016*	*irrational*	ordinary	4.04	.0001	unusual
*justified*	*2.97*	.*0030*	*senseless*	*regular*	*2.84*	.*0045*	*irregular*
stable	3.28	.0010	unstable	*rational*	*2.73*	.*0063*	*irrational*
steady	3.78	.0002	faltering	*justified*	*2.94*	.*0033*	*senseless*
solid	3.66	.0003	fragile	*reliable*	*2.75*	.*0060*	*unreliable*
WEAKER	Social	Endur.	STRONGER	steady	3.01	.0026	faltering
indifferent	3.07	.0021	exciting	solid	3.41	.0006	fragile
irritating	3.88	.0001	pleasant	WEAKER	Social	Plastic	STRONGER
decline	3.66	.0002	progress	exciting	3.38	.0007	indifferent
harmful	2.92	.0035	useful	bright	2.90	.0037	pale
*irrational*	3.43	.0006	*rational*	interesting	3.18	.0015	uninter-ng
*steady*	2.72	.0065	*faltering*	pleasant	3.77	.0002	irritating
scattered	2.88	.0040	dense	smooth	2.71	.0067	rough
MEN with	Z	*p*-level		safe	3.07	.0021	dangerous
WEAKER	Motor	Endur.	STRONGER	useful	3.66	.0003	harmful
estimated		**Reality**	AS MORE:	**significant**	**4.02**	**.0001**	**insignific**
draining	3.31	.0009	stimulat-g	**powerful**	**3.17**	**.0015**	**weak**
abundant	2.89	.0039	popular	***irreplaceab***	***3.10***	**.** ***0020***	***replaceab.***
rough	3.21	.0013	smooth	WOMEN of	Z	*p*-level	
**weak**	**2.97**	**.0030**	**powerful**	WEAKER	Social	Endur.	STRONGER
impossible	2.84	.0045	possible	estimated	**‘Soc**	**At’**	estimated
unknown	2.69	.0072	known	***difficult***	***3.99***	**.** ***0001***	***easy***
*blurred*	*3.47*	.*0005*	*clear*	imaginary	3.59	.0003	real
*irrational*	*3.66*	.*0003*	*rational*	atypical	2.95	.0032	typical
WEAKER	Social	Endur.	STRONGER	rare	4.43	.0000	common
indifferent	3.00	.0027	exciting	unusual	3.48	.0005	ordinary
indifferent	3.00	.0027	exciting	*blurred*	*3.88*	.*0001*	*clear*
pale	3.11	.0019	bright	*irregular*	*3.32*	.*0009*	*regular*
draining	3.63	.0003	stimulat-g	*unreliable*	*3.60*	.*0003*	*reliable*
uninter-g	3.32	.0009	interesting	faltering	3.20	.0014	steady
severe	3.17	.0015	kind	LOWER	Social	Emot	HIGHER
harmful	2.83	.0047	useful	exciting	2.84	.0045	indifferent
rough	3.30	.0010	smooth	stimulat-g	3.26	.0011	draining
**following**	**3.21**	**.0013**	**leading**	useful	3.26	.0011	harmful
*irrational*	*3.22*	.*0013*	*rational*	**leading**	**2.73**	**.0063**	**following**
*senseless*	*3.13*	.*0018*	*justifiable*	natural	2.83	.0046	artificial
*unorgand*	*3.31*	.*0009*	*organized*	possible	2.75	.0060	impossible
finite	3.01	.0026	infinite	WOMEN of	Z	*p*-level	
LOWER	Motor	Emot	HIGHER	WEAKER	Social	Endur.	STRONGER
*ordered*	*2.66*	.*0079*	*chaotic*	estimated	**‘Real**	**ity’**	estimated
*clear*	*3.12*	.*0018*	*blurred*	severe	4.31	.0000	kind
*obvious*	*3.16*	.*0016*	*obscure*	dangerous	2.81	.0049	safe
*regular*	*3.41*	.*0007*	*irregular*	dark	2.81	.0049	light
existent	3.39	.0007	imagined	rough	3.35	.0008	smooth
common	2.70	.0070	rare	difficult	3.01	.0026	easy
stable	2.83	.0046	unstable	**insignific.**	**2.68**	**.0074**	**significant**
LOWER	Social	Emot	HIGHER	*blurred*	*3.62*	.*0003*	*clear*
stimulat-g	3.88	.0001	draining	*irregular*	*2.90*	.*0037*	*regular*
interesting	2.81	.0049	uninter-g	*irrational*	*3.00*	.*0027*	*rational*
pleasant	2.92	.0036	irritating	unstable	3.53	.0004	stable
light	2.66	.0078	dark	fragile	3.81	.0001	solid
useful	2.69	.0071	harmful	MEN with	Z	*p*-level	
smooth	2.90	.0038	rough	WEAKER	Motor	Endur.	STRONGER
**leading**	**3.50**	**.0005**	**following**	estimated	**Work**		AS MORE:
***ordered***	***3.50***	**.** ***0005***	***chaotic***	trivial	2.56	.0106	original
known	2.93	.0034	unknown	pale	4.61	.0000	bright
inevitable	2.77	.0057	improbab.	uninter-g	4.66	.0000	interesting
*clear*	*3.04*	.*0024*	*blurred*	irritating	2.85	.0043	pleasant
*justified*	*2.72*	.*0066*	*senseless*	decline	4.37	.0000	progress
*reliable*	*3.34*	.*0008*	*unreliable*	dark	3.46	.0005	light
stable	2.88	.0039	unstable	bad	3.11	.0019	good
steady	3.37	.0008	faltering	harmful	3.01	.0026	useful
solid	2.76	.0059	fragile	**weak**	**2.78**	**.0055**	**powerful**
MEN with	Z	*p*-level		***discrete***	***3.38***	**.** ***0007***	***continuous***
WEAKER	Motor	Endur.	STRONGER	***one-dim***	***3.83***	**.** ***0001***	***multi-dim***
estimated	**‘Timi**	**ng’**	AS MORE:	artificial	3.09	0.0020	natural
draining	3.40	.0007	stimulat-g	imaginary	3.69	.0002	real
dull	4.00	.0001	sharp	impossible	3.76	.0002	possible
severe	3.03	.0025	kind	unknown	2.70	.0069	known
***inexplicab***	***3.93***	**.** ***0001***	***understanda***	*blurred*	*3.54*	.*0004*	*clear*
artificial	2.88	.0039	natural	*senseless*	*3.33*	.*0009*	*justified*
false	3.38	.0007	true	*unreliable*	*3.92*	.*0001*	*reliable*
imaginary	3.27	.0011	real	*unorgan-d*	*3.00*	.*0027*	*organized*
unknown	4.79	.0000	known	unstable	2.71	.0068	stable
rare	3.34	.0008	common	faltering	4.54	.0000	steady
unusual	2.69	.0071	ordinary	WEAKER	Social	Endur.	STRONGER
*burred*	*3.13*	.*0017*	*clear*	trivial	2.72	.0065	original
*irregular*	*3.42*	.*0006*	*regular*	indifferent	3.57	.0004	exciting
*irrational*	*2.77*	.*0055*	*rational*	pale	3.46	.0005	bright
*imprecise*	*3.22*	.*0013*	*precise*	irritating	2.76	.0059	pleasant
faltering	3.34	.0008	steady	***inexplicab***	***3.21***	**.** ***0013***	***understandb***
SLOWER	Motor	Tempo	FASTER	artificial	4.09	0.0000	natural
draining	3.67	.0002	stimulat-g	false	3.35	.0008	true
uninter-g	3.03	.0025	interesting	*irrational*	*2.85*	.*0044*	*rational*
irritating	3.29	.0010	pleasant	slow	3.04	.0023	fast
severe	3.90	.0001	kind	LOWER	Social	Plastic	HIGHER
decline	2.96	.0031	progress	useful	4.04	.0001	harmful
bad	3.04	.0023	good	progress	4.52	.0000	decline
unknown	3.29	.0010	known	***continuous***	***2.78***	**.** ***0055***	***discrete***
improbabl	4.00	.0001	inevitable	***understan***	***2.75***	**.** ***0060***	***inexplicable***
rare	2.94	.0033	common	real	4.36	.0000	imagined
inaccessib	4.15	.0000	accessible	*clear*	*3.50*	.*0005*	*blurred*
*irregular*	*2.86*	.*0043*	*regular*	*rational*	*3.66*	.*0003*	*irrational*
*inexplicab*	*3.40*	.*0007*	*understanda*	*justified*	*2.93*	.*0033*	*senseless*
*imprecise*	*2.73*	.*0062*	*precise*	*organized*	*2.87*	.*0041*	*unorgan-d*
*unorgan-d*	*3.59*	.*0003*	*organized*	*reliable*	*3.14*	.*0017*	*unreliable*
slow	2.93	.0034	fast	stable	2.89	.0039	unstable
SLOWER	Motor	Tempo	FASTER	steady	2.91	0.0036	faltering
indifferent	3.59	.0003	exciting	WOMEN of	Z	*p*-level	
dull	3.50	.0005	sharp	WEAKER	Social	Emot.	STRONGER
severe	2.91	.0036	kind	estimated	**‘Timi**	**ng’**	AS MORE:
harmful	3.59	.0003	useful	kind	3.21	.0013	severe
superficial	3.11	.0019	deep	pleasant	2.90	.0038	irritating
impossible	2.70	.0069	possible	light	3.23	.0012	dark
*unreliable*	*3.19*	.*0014*	*reliable*	safe	3.52	.0004	dangerous
*unorgan-d*	*2.81*	.*0049*	*organized*	light	2.76	.0058	dark
soft	2.78	.0054	rigid	useful	3.63	.0003	harmful
				**significant**	**3.47**	**.0005**	**insignific.**
				***easy***	***2.78***	**.** ***0054***	***difficult***
				natural	3.31	.0009	artificial
				real	2.86	.0042	imagined
				common	3.79	.0001	rare
				*obvious*	*3.22*	.*0013*	*obscure*
				*rational*	*2.69*	.*0072*	*irrational*
				infinite	2.69	.0071	finite

The font alternates between the scales of the Stimulation factor (underlined), Evaluation (normal), **Power** (**bold**), Complexity (***bold italic***), Reality-Probability (normal), *Organization* (*italic*) and Stability-Limitation (normal). Groups of concepts: “People”(Person, Unknown person, My contemporary, Society), “SocialAt” (social attractors: (Prestige, Reputation, Beauty, Freedom), “Reality” (Reality, Present, Life), “Work” (Work, Task, Activity, Effort), “Time” (Time, Speed, Motion, Development), PastFut” (Past, Future). “SimpOr” (Simplicity, Order).

The results show that in terms of capacity-biases effects, a high number of statistically significant differences were found for male groups contrasted by physical (Motor) Endurance. Men with reported stronger physical endurance estimated work- and time-related concepts significantly more positively than men with reported weaker endurance. The same positive bias, but with a smaller number of statistically significant differences, was found in male estimations of people- and reality related concepts and social attractors. Temperament groups contrasted by Social-verbal Endurance (ERS) showed a positive bias in estimations of socially energetic participants, which was consistent across two sex groups and all groups of concepts.

In terms of lability-related temperamental traits, males with higher scores on the scale of Motor Tempo had significantly more positive estimations of timing-related concepts than the opposite temperament group, but this was not the case for female groups. At the same time males with a faster Tempo of Social-verbal activities had significantly more negative estimations of Power and Timing-related concepts than males with a slower Social Tempo. Men with higher Social Plasticity gave significantly more negative estimations to social attractors and work-related concepts than men with lower Social Plasticity scores.

The differences between social, physical and intellectual endurance were that 1) higher social-verbal endurance was associated with a more general positive bias in estimations, and that 2) intellectual endurance (ERI), i.e. the ability to stay focused on a mental task, was associated in females with a bias that was opposite to social and physical types of endurance. Women with a higher ERI estimated social attractors, work-related and timing-related concepts with more negative bias than women with the lower ERI scores. Men with higher ERI scores however gave more positive estimations of reality-related concepts than males with lower ERI.

In terms of emotionality effects, non-evaluative scales followed the polarity of evaluative scales in a very consistent manner (Fig. 1, Table 4). For example, whenever a temperament group assessed the concepts as more “good” and “interesting” (i.e. with more positive estimations on the scales of Evaluation and Stimulation factors than the contrast group), it would also assess these concepts as more “organized”, “probable”, “real” and “stable” (i.e. also with positive estimations on the scales of Organization, Probability-Reality and Stability factor.

The impact of Emotionality traits was not universal across objects (concepts). It was specific to possible areas of failures and was gender-specific. The most dramatic number of significant differences was observed in temperament groups contrasted by Social Emotionality and Social Endurance. Socially emotional men saw people-related, reality (life)-related concepts and social attractors as more draining, uninteresting, irritating, severe, dark, dirty, cold, false, unknown, improbable, irregular, irrational, senseless, and unstable than socially low-emotional men. Female groups contrasted by Social Emotionality differed in their estimations of timing-related (Time, Speed, Motion, Development), and work-related concepts (Work, Effort, Task, Activity). Socially emotional females saw these concepts more negatively than low-emotional females. Not shown in the Figures or Tables: females with higher Social Emotionality estimated people-related concepts as less interesting (*p* = 0.0017), and life/reality-related concepts as more negative along fours scales of the Evaluation and Stimulation factor) than low-emotional females (*p* = 0.0000–0.0048).

A differential impact of Emotionality related to physical-Motor activities (EMM) in male contrast temperament groups was observed for people-related concepts: men with higher scores in this type of emotionality estimated the concepts Person, Unknown person, Society and My contemporary as more simple and overall more positive than low-emotional men. There were sex differences in contrast EMM groups also in estimations of social attractors and reality-related concepts: while female contrast groups did not show many differences (one in each group of concepts). Men with higher EMM scores estimated social attractors as significantly more imaginary, impossible and unusual, and reality-related concepts as chaotic, irregular, rare, imagined and unstable than low-emotional men.

Overall, male groups had a much higher number of statistically significant differences between contrast temperament groups than females. Males also had more diverse scores on temperament scales than women (their standard deviation was higher than female *SD*s on 9 out of 12 scales (exceptions were Social Plasticity, Motor and Intellectual Emotionality scales) (Tables 2 and 3). An ANOVA comparison of the means revealed male superiority in six temperament scales: Motor-physical Endurance (ERM) (at *p*<0.0000), Motor Plasticity (PLM), Motor Tempo (TMM) (at *p*<0.0025-0.0029), Intellectual Endurance (ERI) (at *p*<0.0042), Intellectual Plasticity (PLI) and Intellectual Tempo (TMI) (at *p*<0.0000). Females had superiority in Social Endurance (ERS) (at *p*<0.003), Social-verbal Tempo (TMS) (at *p*<0.0000), and higher Emotionality in Intellectual (EMI) and Social (EMS) aspects of activities (at *p*<0.0000). The scales of Social Plasticity (PLS) and Emotionality in motor-physical (EMM) activities did not show significant sex differences (Table 3).

The data is deposited on the server of McMaster University, Faculty of Health Sciences at: http://fhs.mcmaster.ca/cilab/DataPLOS1.xls.

## Method, Study 2

This study was investigating the same hypothesis as the Study 1 using a partially different version of the temperament measure.

### Sample

378 undergraduate psychology students of McMaster University (Southern Ontario) (81% of the sample) and volunteers from the same area aged 17–53, M/F  = 140/238 participated in this study in 2006–2008. All participants were fluent in English. After validity procedures were applied the final sample included 131 men, *M_age_ ± SD*  = 21.2±6.97 and 219 women *M_age_ ± SD*  = 20.0±5.23.

### Procedure

All subjects received debriefing, signed an informed consent form, then completed the Compact Structure of Temperament Questionnaire (STQ-77) and participated in the Semantic Task experiment.

The Compact Structure of Temperament Questionnaire (STQ-77) [83], [94], [119]–[120] has 77 statements, assigned to 12 temperamental scales (6 items each) and the validity scale (5 items) listed below. Subjects respond according to a Likert scale format: “strongly disagree (1),” “disagree (2),” “agree (3),” “strongly agree (4)”. The scales are:

1–3: Endurance group, scales of Motor, Social and Intellectual Endurance: the ability of an individual to sustain prolonged physical (ERM, apha  =  0.85), social (ERS, 0.80) or mental (ERI, 0.71) activity.

4–5: Lability group, scales of Motor and Social Tempo: preferred speed of physical activity (TMM, 0.70), speed of speech and reading and of other verbal activities (TMS, 0.75) and Plasticity scale, assessing the ability to adapt quickly to changes in situations, to change the program of action, and to shift between different tasks (PL, 0.71).

6–9: Sensitivity group: Sensitivity to Sensations scale (SS, 0.73), assessing the sensitivity of an individual to basic physical sensations and pleasures, a tendency for sensation-seeking and risk-taking behaviour; Empathy scale (EMP, 0.70) assessing sensitivity of an individual to another person's emotional state, and Sensitivity to Probabilities (PRO, 0.72) scale assessing ability of an individual for adequate understanding and expectations of probable events, the efficient extraction and processing of new knowledge.

10–12: Emotionality group: Self-confidence scale (SLF, 0.70): the tendency to be optimistic and confident (sometimes overly optimistic) in own performance, to ignore other people's warnings and criticism; Impulsivity scale (IMP, 0.74): the lability of emotional reaction, a poor ability to control immediate impulses for actions; Neuroticism scale (NEU, 0.71): low tolerance of uncertainty with expectations of a negative outcome.

13. Validity scale - social desirability tendency in answers. Results within the range of 15–20 on the validity scale should be considered invalid as the respondents are likely to demonstrate positive impression bias in their responses.

The Semantic Task experiment and statistical procedures were similar to Study 1. The only difference was that participants assessed 24, and not 29 concepts, with the concepts Task, Effort, Unknown person, My contemporary and Reputation excluded.

### Results, Study 2

**Figure 2:**
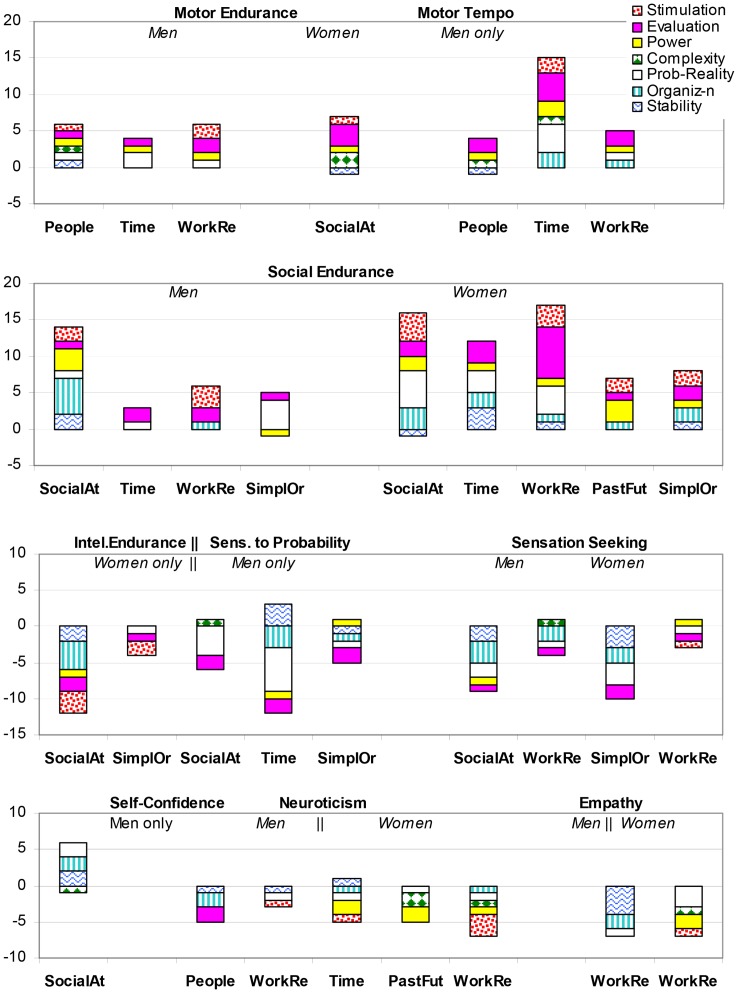
The number of statistically significant differences in estimations of contrast temperament groups, Study 2. Axis Y: the total number of significant differences; the sign indicates the pole of the scales chosen by the group with the higher scores on a given temperament trait. The colours represent the spectrum of these differences along seven factors to which the scales are associated. Axis X: groups of concepts (see text for details).

Figure 2, Tables 2, 5, 6 and S2 summarize the results in the same fashion as was done in Study 1. The concepts were organized into the following groups: “People” (Person, Society), “SocialAt” (social attractors: (Prestige, Beauty, Freedom), “WorkRe” (Work, Activity, Reality, Present, Life), “Time” (Time, Speed, Motion, Development), SimpOr (Simplicity, Order, Faith, Relaxation) and “PastFut” (Past, Future).

**Table 5:** Means (*M*), and standard deviations (*SD*) and ANOVA effects (*F, p*) of sex differences in means on STQ-77 scales (Study 2).

	Men,	N = 131	Women	N = 219	*F*	*p*
*STQ-77 scales*	*M*	*SD*	*M*	*SD*	*(1, 348)*	
Motor Endurance, ERM	17.04	4.29	15.02	4.20	18.59	.0000
Intellectual Endurance, ERI	15.08	3.10	14.22	2.95	6.58	.0107
Social Endurance, ERS	17.92	4.00	18.62	3.58	2.85	.0923
Plasticity, PL	16.08	2.43	15.10	2.50	12.90	.0004
Sensitivity to Probabilities, PRO	16.86	2.63	15.34	2.79	25.50	.0000
Empathy, EMP	18.08	2.82	17.23	2.98	6.84	.0093
Motor Tempo, TMM	17.06	3.54	15.35	3.37	19.80	.0000
Sensitivity to sensations, SS	16.60	2.74	16.31	2.85	0.84	.3590
Social Tempo, TMS	15.85	2.88	17.27	3.50	15.41	.0001
Self- confidence, SLF	17.05	2.58	16.02	2.56	13.12	.0003
Impulsivity, IMP	15.65	2.79	15.46	2.70	0.41	.5246
Neuroticism, NEU	15.71	3.01	16.27	2.97	2.88	.0907

**Table 6:** Selected list of the significant differences in estimations of contrast temperament groups, Study 2.

MEN with	Z	*p*-level		WOMEN of	Z	*p*-level	
WEAKER	Social	Endur.	STRONGER	WEAKER	Social	Endur.	STRONGER
estimated	**‘Soc-**	**ialAt’**	as more:	estimated	**‘Soc-**	**ialAt’**	as more
dull	3.01	.0026	sharp	indifferent	3.45	.0006	exciting
abundant	3.36	.0008	popular	pale	3.48	.0005	bright
decline	3.22	.0013	progress	uninterest-g	2.75	.0060	interesting
**insignific.**	**3.45**	**.0006**	**significant**	dull	3.64	.0003	sharp
**following**	**2.92**	**.0036**	**leading**	dirty	2.84	.0045	pure
**dependent**	**2.99**	**.0028**	**independ.**	harmful	2.86	.0043	useful
imagined	2.86	.0043	existent	**insignific.**	**3.03**	**.0025**	**significant**
*blurred*	*3.21*	.*0013*	*clear*	**small**	**2.93**	**.0034**	**large**
*irregular*	*3.47*	.*0005*	*regular*	imagined	2.74	.0062	existent
*irrational*	*2.96*	.*0031*	*rational*	imaginary	4.06	.0000	real
*imprecise*	*3.63*	.*0003*	*precise*	impossible	3.30	.0010	possible
*unorgan-d*	*4.48*	.*0000*	*organized*	atypical	4.63	.0000	typical
unstable	3.22	.0013	stable	rare	3.29	.0010	common
fragile	3.24	.0012	solid	*blurred*	*3.19*	.*0014*	*clear*
				*irregular*	*3.19*	.*0014*	*regular*
lower	Sensat	Seek.	higher	slow	2.88	.0040	fast
progress	3.23	.0012	decline	weaker	Intell.	Endur.	stronger
**leading**	**3.26**	**.0011**	**following**	exciting	3.43	.0006	indifferent
true	3.27	.0011	false	bright	3.19	.0014	pale
ordinary	4.70	.0000	unusual	stimulating	2.90	.0038	draining
*justified*	*3.59*	.*0003*	*senseless*	kind	2.82	.0048	severe
*organized*	*3.08*	.*0021*	*unorganized*	safe	2.73	.0063	dangerous
*understand*	*4.12*	.*0000*	*inexplicable*	**deep**	**3.22**	**.0013**	**superficial**
active	2.76	.0058	passive	*regular*	*3.38*	.*0007*	*irregular*
solid	4.31	.0000	fragile	*rational*	*2.92*	.*0035*	*irrational*
lower	Sensit.	toProb	higher	*precise*	*4.02*	.*0001*	*imprecise*
kind	3.46	.0005	severe	*organized*	*3.25*	.*0011*	*unorgan-d*
good	3.00	.0027	bad	stable	3.58	.0003	unstable
***simple***	**2.61**	**.0091**	***complex***	steady	4.19	.0000	faltering
known	3.17	.0015	unknown				
existent	3.96	.0001	imagined	WOMEN of	Z	*p*-level	
common	4.61	.0000	rare	weaker	Social	Endur.	stronger
typical	3.60	.0003	atypical	estimated	**‘Work**	**Real’**	as more
MEN of	Z	*p*-level		draining	3.10	.0019	stimulat-g
weaker	Motor	Endur.	stronger	uninterest-g	3.81	.0001	interesting
estimated	**‘Work**	**Real’**	as more	dull	2.88	.0039	sharp
draining	3.42	.0006	stimulat-g	irritating	3.07	.0021	pleasant
abundant	3.26	.0011	popular	severe	3.68	.0002	kind
dirty	2.72	.0065	pure	harmful	2.71	.0067	useful
bad	2.95	.0031	good	cold	2.84	.0043	warm
**weak**	**3.07**	**.0021**	**powerful**	decline	3.15	.0016	progress
impossible	2.76	.0057	possible	rough	2.62	.0068	smooth
slower	Motor	Tempo	faster	dark	3.03	.0025	light
indifferent	3.66	.0003	exciting	**insignific**	**4.13**	**.0000**	**significant**
bad	3.51	.0005	good	artificial	2.84	.0044	natural
**weak**	**4.06**	**.0000**	**powerful**	imagined	2.73	.0063	existent
imaginary	2.61	.0090	real	imaginary	3.05	.0022	real
unrestraind	3.03	.0025	restrained	impossible	3.68	.0002	possible
weaker	Social	Endur.	stronger	*senseless*	*2.94*	.*0032*	*justified*
indifferent	2.92	.0035	exciting	faltering	3.23	.0012	steady
uninterest-g	3.12	.0018	interesting	lower	Neuro-	ticism	higher
abundant	3.39	.0007	popular	exciting	3.19	.0014	indifferent
severe	3.42	.0006	kind	bright	3.08	.0020	pale
bad	3.12	.0018	good	interesting	4.54	.0000	uninteres-g
*irrational*	*3.08*	.*0021*	*rational*	**significant**	**3.63**	**.0003**	**insignific.**
MEN of	Z	*p*-level		***continuous***	***3.01***	**.** ***0027***	***discrete***
slower	Motor	Tempo	faster	possible	2.89	.0038	impossible
estimated	**‘Timi**	**ng’**	as more	*justified*	*2.83*	.*0045*	*senseless*
draining	4.17	.0000	stimulating	WOMEN of	Z	*p*-level	
slow	3.77	.0001	fast	weaker	Social	Endur.	stronger
irritating	2.80	.0051	pleasant	estimated	**‘Timi**	**ng’**	as more
severe	3.41	.0006	kind	irritating	3.17	.0015	pleasant
cold	2.87	.0040	warm	cold	3.03	.0024	warm
bad	3.34	.0008	good	severe	2.70	.0069	kind
**weak**	**2.93**	**.0034**	**powerful**	**weak**	**3.02**	**.0025**	**powerful**
**insignific.**	**2.76**	**.0058**	**significant**	false	2.74	.0060	true
***one-dim***	***3.12***	**.** ***0018***	***multi-dim***	imagined	3.25	.0012	existent
impossible	3.46	.0005	possible	impossible	3.18	.0014	possible
improbable	4.09	.0000	inevitable	*blurred*	*3.38*	.*0007*	*clear*
rare	3.30	.0009	common	*imprecise*	*3.06*	.*0022*	*precise*
imagined	2.91	.0036	existent	faltering	2.71	.0066	steady
*imprecise*	*2.80*	.*0051*	*precise*	unstable	3.18	.0014	stable
*unreliable*	*3.25*	.*0011*	*reliable*	fragile	3.51	.0004	solid
lower	Sensit.	toProb	higher	lower	Neuro-	ticism	higher
good	4.11	.0000	bad	exciting	3.56	.0004	exciting
warm	2.99	.0027	cold	**significant**	**2.77**	**.0057**	**significant**
**significant**	**3.41**	**.0006**	**insignific.**	**large**	**3.50**	**.0004**	**large**
existent	4.65	.0000	imagined	possible	2.77	.0057	possible
known	5.19	.0000	unknown	dense	3.56	.0004	dense
ordinary	2.76	.0058	ordinary	infinite	3.11	.0019	infinite
typical	5.04	.0000	atypical	WOMEN of	Z	*p*-level	
common	4.42	.0000	rare	weaker	Social	Endur.	stronger
real	2.83	.0047	imaginary	estimated	**‘Simpl**	**Or’**	as more
*clear*	*3.63*	.*0003*	*blurred*	stimulating	2.66	.0078	draining
*regular*	*3.07*	.*0021*	*irregular*	arouses	2.97	.0029	calms
*reliable*	*2.67*	.*0067*	*unreliable*	kind	3.16	.0016	severe
active	3.88	.0001	passive	cold	3.06	.0022	warm
fast	3.81	.0001	slow	**insignific-t**	**3.71**	**.0002**	**significant**
unrestraind	4.30	.0000	restrained	*irregular*	*3.09*	.*0020*	*regular*
MEN of	Z	*p*-level		*irrational*	*2.91*	.*0036*	*rational*
weaker	Social	Endur.	stronger	unstable	3.31	.0009	stable
estimated	**‘Simpl**	**Or’**	as more	MEN of	Z	*p*-level	
cold	3.10	.0019	warm	lower	Sensat	Seek.	higher
**massive**	**3.20**	**.0014**	**delicate**	estimated	**‘Simpl**	**Or’**	as more
unknown	3.61	.0003	known	good	2.98	.0029	bad
atypical	3.67	.0002	typical	light	3.03	.0024	dark
rare	2.96	.0031	common	ordinary	2.83	.0047	unusual
imagined	3.77	.0002	existent	known	2.82	.0048	unknown
lower	Sensit.	toProb	higher	imagined	2.93	.0034	existent
pleasant	2.74	.0062	irritating	common	2.98	.0029	rare
good	2.80	.0051	bad	*reliable*	*2.90*	.*0037*	*unreliable*
**smooth**	**2.68**	**.0074**	**rough**	*obvious*	*3.01*	.*0026*	*obscure*
true	3.09	.0020	false	solid	3.14	.0017	fragile
*rational*	*4.07*	.*0000*	*irrational*	steady	3.72	.0002	faltering
stable	2.82	.0048	unstable	stable	2.95	.0031	unstable

The font alternates between the scales of the Stimulation factor (underlined), Evaluation (normal), **Power** (**bold**), Complexity (***bold italic***), Reality-Probability (normal), *Organization* (*italic*) and Stability-Limitation (normal). The groups of concepts: “People” (Person, Society), “SocialAt” (social attractors: (Prestige, Beauty, Freedom), “WorkRe” (Work, Activity, Reality, Present, Life), “Time” (Time, Speed, Motion, Development), SimpOr (Simplicity, Order, Faith, Relaxation).

In regards to the “projection through capacities hypothesis”, males with stronger Motor Endurance (ERM) estimated people-, work/reality- and time-related concepts in more positive terms than males with a weaker endurance. Females with stronger ERM estimated social attractors in more positive terms than females with weaker ERM. Both male and female temperament groups with stronger Social-verbal Endurance showed a universal positive bias in their estimations, especially for social and work/reality-related concepts, in comparison to participants with lower sociability. A trait of self-confidence created a positive evaluative bias only in estimations of social attractors, and only in men.

In terms of tempo-related scales, the most significant positive bias in estimations of men with higher Motor Tempo was found in their evaluation of time-related concepts.

Similar to the findings of Study 1, social, physical and intellectual endurance were associated with different biases in estimations. When significant differences were found between the temperament groups contrasted by physical and social endurance, the people with stronger endurance had a more positive evaluative bias than people with weaker endurance. Intellectual endurance, however, was associated with a negative evaluative bias. Women with higher Intellectual Endurance estimated social attractors as more unstable, disorganized, negative and non-stimulating compared to women with the lower ERI. No differences were found in the male groups.

In terms of emotionality, the differences on non-evaluative scales (i.e. included in factors of Probability-Reality, Complexity, Stability and Organization) followed the polarity of differences on evaluative scales (included in factors Evaluation, Stimulation, Power). Men with higher Neuroticism scores on the STQ-77 estimated people-related concepts in more negative terms than low-neurotic men, but almost no differences were found for social attractors in both male and female groups contrasted by neuroticism. Both men and women with high Neuroticism and high Empathy estimated work- and reality-related concepts with negative bias in comparison to low-neurotic and low-empathic groups. Highly neurotic women also gave more negative evaluations to timing-related concepts and to the Past and Future than their contrast temperament group.

Temperament traits related to different types of sensitivity – sensitivity to sensations, sensitivity to probabilities, empathy and neuroticism – all had a polarity of bias, which was opposite to those of physical and social endurance traits (in the cases when significant differences were found). Both men and women with higher Sensitivity to Sensation (SS) scale of the STQ-77 gave more negative estimations of work/reality-related concepts than participants with lower sensation seeking. The Sensitivity to Probabilities scale, which measures a person's ability to learn and to derive causal relationships, showed effects in the male contrast group: men with high scores on this scale estimated social attractors and time-related concepts as significantly more bad, cold, insignificant, imaginary, unusual, blurred, irregular, unreliable and slow than men with lower scores.

Men with higher ranks on Sensation Seeking scale estimated the concepts Simplicity, Order, Relaxation and Faith as significantly less stable, organized (!), and real than low sensation-seekers. At the same time participants with high Social Endurance (sociability) in both sex groups gave more positive evaluations than their contrast group.

Overall male contrast temperament groups had a higher number of statistically significant differences between them than did the female groups (Table 6, Figure 2, Table S2). An ANOVA comparison of the means revealed male superiority in seven temperament scales: Motor-physical Endurance, Motor Tempo, Sensitivity to Probabilities (at *p*<0.0000), Plasticity, Self-Confidence (at *p*<0.0003), (EMP) (at *p*<0.009) and Intellectual Endurance (at *p*<0.01). Females had superiority in Social-verbal Tempo (at *p*<0.0001).

## Discussion

In theory, and from the lexical approach perspective, there should not be any temperament-related differences in the assessment of amodal common concepts using very common adjectives, especially in people with at least high school education in a developed Western country. The experimental material in our studies had a very general and non-biased nature. Yet, a complex pattern of significant temperament-related differences was found in semantic perception even for words with a very high level of generality. The results were more profound for the STQ-150, in comparison to the STQ-77, but this might be due to the size of the samples.

Our *projection through capacities* hypothesis was supported in comparisons of estimations of temperament groups contrasted by two (physical and social-verbal) types of endurance and tempo. When significant differences were found, participants with stronger physical or social endurance in both studies gave more positive ratings to concepts than participants with weaker endurance of these types (Figures 1–2). Men with weaker motor endurance but with faster social-verbal tempo had even more negative estimations of work- and reality-related concepts than women with the same traits (Figure S1, Table S3). This was consistent with the positive evaluation bias observed in extraverts in other studies [31]–[33]. Significantly fewer differences were found between the temperament groups contrasted by intellectual endurance, and when such differences were found they had a pattern opposite to the groups contrasted by the other two types of endurance (physical and social). In line with our hypothesis, men with higher Motor Tempo and Endurance gave more positive ratings to timing-related concepts than did their opposite groups. It was interesting that in both studies significant differences were found for the concepts Order and Simplicity (i.e. the concepts requiring an opposite to lability) between female temperament groups contrasted by lability traits (Motor Tempo in Study 1 and Impulsivity in Study 2). Faster and more impulsive females saw these objects in more negative terms.

In regards to our “*language-bias*” hypothesis, we found a strong pro-social bias in estimations of general concepts. The scales of Social-verbal Endurance, and Social emotionality/Neuroticism were associated with much more significant differences in estimations, in comparison to all other temperament scales, and this was observed in both studies. Social endurance had the most number of significant differences, and people with high social endurance had a tendency for more cheerful estimations, even when it came to non-social concepts (such as Work-Reality, Simplicity-Order or Timing groups). Social emotionality, i.e. sensitivity to failures in *social* activities, produces a much stronger negative affective bias in meaning attribution than sensitivity to failures in *physical* activities.

Moreover, the concepts related to social attractors and “people” had the highest number of significant differences between temperament groups. If the strongest effects, i.e. the largest variance in data are produced by the difference between estimations of social vs. non-social people, and to a lesser degree – by any other temperament types, then the factors resulting from public assessments of individuality would reflect mostly socialization aspects. The pro-social bias of language skews the frequency of common lexical descriptors related to socialization vs. other aspects of behavior and therefore makes these descriptors an unreliable source of information, especially in regards to psychological phenomena, which has a strong social component. Such a pro-social bias of lexical material in semantic processing supports our arguments about flaws (namely observer's bias) in the lexical approach as a method of investigation of the structure of some objects. If the strongest effects in lexical approach studies are induced by the estimations of either socially emotional or socially active people, this makes modeling within the lexical approach “a science of extraverts”, with limited benefits for general differential psychology.

Our third hypothesis was also supported, and this finding was in line with the neuropsychological reports of the interlocking of emotional processing with attention and perception [20]–[22]. Overall the pattern of our results showed the existence of an initial evaluative stage in human semantic perception, which uses two emotional poles even in estimations of abstract neutral concepts. A strong effect of *emotionality bias* was found in a universal tendency across objects for non-evaluative scales to follow the same polarity as the scales for the Evaluation and Stimulation factors (for example, whenever a contrast temperament group assessed a concept as more “good” and “interesting”, it would also assess this word as more “organized”, “probable”, and “real”). Such grouping (“gluing”) of non-evaluative scales with evaluative criteria was described in Kelly's theory and was likely the reason for Osgood's factors to have a strong evaluative content. It is likely that in the perception of the words related to more concrete objects, this primary stage of meaning attribution is followed by other stages of detailed, knowledge-, education-, experience- and intelligence-driven meaning attribution.

More importantly, the inherent bipolarity and evaluative nature of amodal semantic perception means that this bipolarity can be projected onto the properties of objects of estimation. It is likely that when the lexical approach asks people to estimate individual characteristics of other people using just verbal descriptors, bipolar evaluative bias will dominate over other criteria of assessment. This emotionality bias, combined with the social nature of language, would divide all non-evaluative features of an object into groups of features related to the interests of the society. In the example of the models of individuality offered by the lexical approach, these categories would relate to social approach and withdrawal behavior. Such a division would present the perspective of a socialized and emotional observer, judging the object's (i.e. personality's) features primarily from the point of view of socialization, but omitting other important (non-evaluative) features of the object. As a result, when the lexical approach or parental verbal observations are used to derive a model of personality or temperament “in the way how people see it”, it is natural to expect that the biggest dimensions of the model would be Extraversion and Neuroticism, or Approach and Withdrawal, or Positive/Negative emotional dispositions.

Even more problematic are the claims of the lexical approach that this method found the structure of all biologically based individual differences. In spite of the intensive promotion of the Big Five model, it does not correspond to the findings in experimental (i.e. more objective) and neurophysiological studies in differential psychophysiology. These findings indicated that there are important biologically based characteristics, that are unrelated to socialization or emotional evaluation, and therefore were unnoticed by a human observer of personality or temperament structure. For example, the lability of behavior (mobility-rigidity of generation of an action, impulsivity, preferred tempo of performance) and traits related to the types of preferred reinforcers (sensations seeking, empathy, causal thinking) were differentiated from endurance traits in experiments on the properties of nervous systems and in several temperament models [78]–[91], [94]–[95], [117]–[118], but were missed in lexical approach models of individuality. Moreover, extraversion, described as a dimension related to the energetic aspects of activity, missed a differentiation between several types of endurance: social-verbal (ability to sustain prolonged conversations), mental (ability to stay focused on mental tasks) and physical. Yet, the differentiation between these three types of endurance is in line with the functional specialization of temporal, frontal and sensory-motor cortex [83]. (Note that recently, to accommodate these findings Big Five researchers had to use additional techniques in factor analysis, presenting sociability, impulsivity, positive affect, empathy, self-confidence and (in some models) sensation seeking as “second order” traits, i.e. components of extraversion. This did not help to overcome a bipolar emotional division of traits: positive affect was identified as a part of Extraversion, and negative affect – as a part of Neuroticism, even though affective systems are based on different neurophysiological systems than other components of these traits).

There was an aspect of emotionality bias, which we did not expect in our hypothesis, but which suggests an interaction between emotionality and capacities-related biases. This aspect relates to a specificity of emotional sensitivity to lexical material, even when the most amodal and abstract material is used. In line with observations in clinical psychology, our results showed that people with higher sensitivity to failure and neuroticism had more negative estimations of neutral abstract concepts, i.e. emotional negativity bias. Such effects were, however, far from being universal across both gender groups and the objects of estimation. Negative affect had a tendency to color the perception of emotional people in relation to specific possible areas of their failures, and not blindly to all objects. For example, *socially* emotional and neurotic men in both studies perceived people-related and reality-related concepts more negatively than their contrast groups, but men who were sensitive just to their failures in *physical* activities saw people-related concepts significantly more positively than low-emotional men. Interestingly, when temperament was not considered, males estimated social attractors (Beauty, Prestige, Reputation, Power) more positively than women while few sex differences in estimations were found for the concepts describing “people” [101].

Further evidence of the impact of emotionality in an object-specific manner was that socially emotional and neurotic women perceived time-related concepts in more negative terms than other concepts. This is consistent with the more negative estimations of women on timing concepts when temperament differences were not taken into account [76], [121] and significantly lower scores on the temperament scales of Motor Tempo in women, in comparison to men in both studies (Tables 3–4). To integrate the results from Trofimova previous [101] and the present studies it can be proposed that, judging by lower emotionality scores on temperament scales in men, it almost looks like men usually care less about their social failures than do women, but those who do care (i.e. men with Social Emotionality) have really big issues, namely, with people, and much less so with social values. Women's emotionality, however, simply affects their meaning attribution when it relates to the perception of concepts of speed and timing, in which they feel inferior.

Similarly to the results of the studies of sex differences in the semantic perception of lexical material [101], [121], men had more negative evaluations for work- and reality-related concepts than women (Figure S1 and Table S3). This coincides with the findings of Study 2 that both men and women with high sensation seeking evaluated these concepts more negatively than participants with lower sensation seeking, and that men with higher sensation seeking estimated the concepts Simplicity, Order, Relaxation and Faith as significantly less stable, organized, and real than low sensation-seekers. Men are reported to have higher risk- and sensation-seeking behavior, especially in youth [91], and it is natural to see that concepts related to routines are perceived more negatively by men than by women [101], [121]. This study did not find significant differences between men and women on sensation seeking per se, but it is possible that it is extreme temperament traits that induce the biases in semantic perception. For example, men with both lower and higher social endurance gave significantly lower evaluations for work- and reality-related concepts than women with such traits (Figure S1).

Different types of sensitivity (sensitivity to probabilities, to other people's state (empathy), to physical sensations (sensation seeking), neuroticism) were associated in Study 2 with either no differences or negative estimation biases by people with high scores on such sensitivities. Here we see how easy it is for humans to be confused about the structure of individual differences when different traits “look the same” on a bipolar dimension related to emotional or social behavior. People with high sensitivity of very different types (sensation seeking, empathy, probabilistic thinking), people with high attentive abilities and people with lower physical and social endurance are likely all to have negative evaluative biases and could be classified as one group (previously known as “introverts”). People with high endurance and tempo, but of different kinds, as well as people with poor attention (intellectual endurance) are likely to exhibit positive evaluative biases, and from the emotionality and socialization point of view they would all be in a group of “extraverts”. Such emotional biases in semantic perception, endorsing socialization-based categories over other structural analysis was likely the reason why early differential psychologists came up with two-dimensional models based on either two poles of emotionality or dimensions of Energy-Strength-Activity-Arousal-Extraversion and Emotionality-Neuroticism.

These findings and arguments question the validity of the lexical approach in differential psychology, which derives the structure of human individuality based upon people's estimations of verbal material (see Discussion of the Controversial Issues for further discussion on related controversial issues). The coupling of several types of biases in the semantic perception of lexical material likely affects the way that scales group into factors in the factor-analytic models of the lexical approach, and masks important objective features of the assessed objects. Repeating the application of this approach in dozens of languages brings consistent results because it doesn't change the social-evaluative nature of lexical material and doesn't improve the flaws of this approach. Besides, the use of linear factor analysis, which looks for independent dimensions, is hardly appropriate in psychological investigations due to nonlinearity, feedback and contingent relationships (i.e. interdependence) between psychological characteristics. Similar to deriving a structure of an object by measuring its shadows on the walls, deriving the structure of biologically based individual differences from people's lexical appraisals of observable behavior is likely not a very informative scientific method.

In summary, our studies showed that capacities-related, language-related and emotionality biases in semantic perception make people very unreliable observers, especially when lexical material is used and when it comes to assessment of social or people-related concepts.

The limitation of the study was related to the use of self-evaluation tests to assess the temperament traits. Considering that 12 temperament traits had to be measured in the same sample, only one method could be realistically implemented: the use of self-report test with the calculation of the rank of capacity to which an individual feels a given trait was developed. The use of a rank-based instead of a value-based system for classification of subjects into the contrast temperament groups (as described in Method section) hopefully addressed this limitation.

## Conclusions

Our studies investigated an impact of biases related to biologically-based capacities, language and emotionality on the semantic perception of lexical material. All three types of biases were found, even though the lexical material was of the most neutral, abstract and amodal nature.

In line with the “projection through capacities” hypothesis and previous findings, we found that people with higher physical and social endurance gave more positive evaluations to neutral concepts than people who felt that their endurance was rather weak. Moreover, participants with faster physical tempo gave more positive estimations to time-related concepts than participants with slower tempo. These findings reflect on another aspect of embodiment in cognition: such embodiment emerges not only as an impact of the situational physical state of a body, but also as a contribution from the consistent dynamical (potential rather than situational) capacities of the body in the semantic perception of lexical material.

A language bias in lexical material was identified when experimental groups contrasted by social emotionality, social endurance and social tempo showed the highest number of effects than the groups contrasted by other abilities. The concepts related to social objects had more significant differences between these contrast temperament groups than the non-social concepts.

A strong impact of emotionality appeared in findings that non-evaluative criteria for categorization (related to complexity, organization, stability and probability of occurrence of objects) followed the polarity of evaluative criteria, and did not show even a weak independence from this polarity. Moreover, neurotic people did not have a universal negative bias in their perception, and negativity bias was rather specific to words describing potential areas of failure in various contrast groups. This was an indication that emotionality bias overpowers semantic perception and collapses differentiation of all other possible descriptors to bipolar emotional criteria.

Overall these findings suggest that people's estimations of lexical material related to human behavior are 1) capacities-biased; 2) unreliable, judging by the opposite patterns in estimation of different temperament groups; 3) influenced by the social nature of language, designed to improve processes of socialization and social interaction, and 4) driven by emotionality, which shrinks the dimensionality of possible criteria into bipolar evaluative constructs. The described biases in estimations of lexical material lead to a collapse the complexity of perceived objects into two or even one dimension based on emotional valence. The study shows that such a collapse happens even for the most neutral and abstract lexical constructs.

This questions the validity of the lexical approach as a method for the objective study of psychological phenomena (including the example of biologically-based individual differences, which was discussed in this article).

## Discussion of the Controversial Issues

This paper suggests that the lexical approach is not an appropriate tool for the investigation of biologically-based traits (called in differential psychology “temperament”). The lexical approach might still be a valid tool for the investigation of social-verbal phenomena, including the influence of lexical processing on the perception of personality differences. The limitations of the lexical approach discussed here relate to its weakness in representation of biologically-based systems of individuality and not to the way in which socialization shapes our perception of personality types. These comments will likely meet objections from personality psychologists using the Big Five model. During the process of review and revision of this article several issues were discussed that are relevant in addressing such objections. The author is grateful to the reviewers and to the editor for suggestions for clarifying the author's position on the following issues.

1 Lexical approach is an analysis of relationships between lexical descriptors of behavior, and not actual behaviour. For those who defend lexical approach models it is useful to keep in mind the nature of the material that these models are based on. The dates of publication are given here to underline the time line of this research. This approach started when Allport (1937) suggested that since most relevant personality characteristics are encoded in natural language we can derive all aspects of individuality just using language descriptors. It was assumed that even biologically based characteristics will be fairly reflected in language. Now we know that this is not true, and that language is by nature a social invention, designed to reflect primarily socialization aspects of human life, and not biological factors of individuality. Moreover, there is a strong positive emotionality bias associated with socialization processes and a negative emotionality bias in people with high emotional arousal. A more detailed review of lexical approach research can be found in John and Srivastava (2001), but in brief the Allport-Odbert collection consisted of about 18000 personality descriptors, which several American psychologists tried to sort out, including Cattell (1945). Cattell selected a subset of 4,500 descriptors, converged it to 35 variables and 16 factors, and developed his famous 16-PF personality test. By the way, similarly to the modern Big Five authors, Cattell also claimed that his 16 factors showed excellent correspondence across methods, such as self-reports, ratings by others, and objective tests; Similarly to the Big Five, Cattell's 16-PF test was translated into many languages and widely distributed in the psychological market. The main criticism of the test was related to its poor psychometric properties, but this is likely due to flaws in the research methodology, and not the psychometric product reflecting the dimensions obtained at the 1st stage.

Several other American researchers used factor analysis of other lists of descriptors: Fiske (1949) used 22 descriptors from Cattell's list and received 5 factors; Norman (1967) compiled a new list of personality descriptors and sorted them into 75 variables, which Goldberg (1990) used as scales in his FA. Goldberg had a series of studies using 1710, 475 or 435 trait adjectives with various groupings into clusters and factors. In several studies he used self and peer ratings using the selected adjectives, and then conducted FA on his data. He consistently received a 5-factor solution, similar to the 5-factor solution received by Tupes and Christal (1961), Norman (1963), Borgatta (1964), Digman and Takemoto-Chock (1981) and then promoted by McCrae and Costa (1992). Moreover, in cross-cultural studies the same approach was used: it started from the collecting of lexical descriptors in other languages, with application of FA to group these descriptors. After difficulties replicating the same 5-factor structure in several languages, cross-cultural studies shifted to simple adaptation of the NEO-FF to other languages and verification of the psychometric properties of new versions of the test.

In other words, the Big Five was developed based on research that used subjective selection of lexical descriptors, and self- and peer assessment of correspondence between (only these) descriptors and observable behavior. And that is what the Big Five represents: a consistent model of how humans reflect individuality using language, no more. There were no considerations of findings in neuroanatomy, neurochemistry, experimental psychology, observations of behavior of people or animals in real situations – none of this was used at the research stage leading to the development of the Big Five. In this sense we can say that the Big Five does not represent the structure of temperament or the structure of biologically based traits, even though lexical perception reflects some elements of it.

2 Why the results of this study do not confirm the validity of Big Five model of personality but rather show its deficits. This article suggests that models of lexical approach reflect only relationships between the lexical personality descriptors that affected by three types of biases, and do not present biologically based systems of human individuality. We noted that there are at least three biases that are present in human lexicon and that can compromise the results of factor analysis in lexical approach: too many words related to socialization, in comparison to other words (socialization-language bias); negativity bias in words related to emotionality; and more words related to energetic rather plasticity aspects of activities (capacities bias). Plus the descriptors of individual behaviour that the lexical approach is using are borrowed from common language and not from scientific language (experimental studies, clinical observations, modeling, theoretical research). As the result, what the lexical approach found is not the structure of biologically-based regulatory systems (temperament), but the socialization and emotionality biases in perception of lexical personality descriptors. Our study investigated whether temperament traits describing socialization and emotionality will show any interaction, or show different interactions with the perception of verbal material than traits that do not relate to socialization and emotionality. After all, as was described in the article, the impact of emotionality on cognition was shown to be strong and not under the control of the individual. The study showed is that in spite of multiple components within temperament structure, temperament traits influence semantic cognition in concert with the described biases, at least with the negativity bias of emotionality and positivity bias of socialization. The way that they interact (more emotional people indeed have more negative estimations and more social or energetic people have indeed more positive estimations) confirms the interlocking between emotionality, capacities and sociability, on the one hand and cognition, on the other hand. Suggestions of emotionality- and sociability-related biases in the assessment of lexical material are bad news for the lexical approach, and having confirmation of such biases using contrast temperament groups is even worse news.

These biases overshadow temperament traits (neurophysiological systems of regulation) which are not related to socialization or emotionality: plasticity, tempo, impulsivity, differentiation between regulatory systems of mental, socio-verbal and physical aspects of behavior. This means that estimations of lexical material collapse the complexity of perceived objects into two or even one dimension based on emotional valence. The study shows that such a collapse happens even for the most neutral and abstract lexical constructs.

For example, if the descriptors referred to elementary particles and only reflected positive-negative charge-related observations (but not spin, mass, strangeness etc.) we would not be able to differentiate between the particles comprising matter and fields (fermions vs. bosons), or stable and unstable particles. Moreover, neutral particles would not be described at all. Similarly, emotionality bias divides perceived properties into positive and negative valence grouping very different traits into the one category and missing emotionally neutral properties out of its analysis. The lexical approach completely misses the regulatory characteristics related to lability of behaviour (plasticity-rigidity, tempo of verbal or physical activities). Yet, these characteristics are well-known for being based on biological systems. Pathologically low plasticity (perseverance) has been described in clinical cases of frontal lobe damage for over 70 years. Contrary to unification of endurance and tempo under one dimension of Extraversion in the Big Five model, these characteristics are based on different biological systems: just consider the difference between capacities used by marathoners and sprinters.

In summary, the study showed how the emotional cognition of lexical material collapses the dimensionality of perceived phenomenon, and this is not necessarily good news for the Big Five theory. Findings within the lexical approach are useful for investigations of verbal processes within cognitive psychology, but we should not mix common human beliefs with scientific findings in neurophysiology and differential psychology.

3 What the lexical approach missed. As noted above, the three biases in the perception of lexical material collapse the complexity of regulatory systems into two dimensions based on emotional valence. Let us briefly list the aspects of biological systems of regulation of human behavior that are being hidden during such a collapse. It is almost impossible to summarize all important findings in temperament research, neuroanatomy and neurochemistry which relate to these aspects, but here are just several examples:

- The findings in neurochemistry indicate that our behaviour is regulated by systems related to the functional aspects of construction of an action: orientation, programming and energetic maintenance (endurance) of an action [92]–[93], [106], [122]–[129]. These functional aspects have leading neurotransmitters regulating them (in concert with other neurotransmitters): NE-based orientation system, DA-based programming and integration system and 5-HT maintenance/performance system [28]–[29], [92], [130].
- These three aspects are regulated differently during routines, habit/skills formation and habit use – such a habit management system is based on GABA/Glutamate exchange with the three other neurotransmitter systems. Moreover, there are several additional levels (hypothalamic hormones, neuropeptides including opioid receptors) regulating the same functional aspects of behaviour, especially in the deterministic aspects of behaviour [24], [28]–[29]. Neuroanatomically cortical vs. striatum integration of an action relates to the differences between regulation of novel/complex vs. routine aspects of the action (since [104]–[105] literature is extensive). In this sense a separation between temperament traits related to probabilistic vs. deterministic aspects of behaviour was useful in temperament research.
- There are multiple reports indicating that nonlinearity, contingency and feedback processes are essential properties of literally every single neurochemical system of behavioral regulation: serotonin [130]–[131], monoamines [92], [132]–[135], prolactin [136], hypocretins [137]–[140], and HPA hormones [141]. What nonlinearity and feedback properties mean is that a linear increase of one parameter (call it a factor if you wish) does not give a proportional response in behaviour (increase or decrease on some observable trait), but instead can have several, often opposite responses in observations. What contingency means is the presence of “if this… then..” in 2-, 3- and multi-way relationships between chemical agents regulating our behaviour. Examine any decent handbook of Neurochemistry (for example, [135]) to appreciate the complexity of our regulatory system. There is no way that common adjectives related to human behaviour would carefully reflect this complexity if common people, and even a majority of psychologists do not know how their behaviour is regulated (neurochemically) inside their own body.
- In spite of the contributions of oxytocin and dopamine systems to th regulation of behaviour they are only a small part of the neurochemistry of behavioural regulation. Moreover, numerous neuropeptides and hormones likely contribute more to biological systems of behavioral regulation (i.e. temperament) than monoamine neurotransmitters.
- In clinical psychology and psychiatry there were several observations as well as analyses of the effect of neurotransmitters that resulted in multiple models of temperament (or character) [81]–[82], [85].
- Developmental psychologists were using parent observations of their children in the study of temperament for more than half of the last century. Even though parents are not the most reliable raters, the fact that these studies used standardized observations of behavioral elements of real individuals (and not verbal descriptors of such elements) makes these studies more valuable contributors to differential psychology than the lexical approach [78]–[80], [90].

4 “A cat knows whose meat she ate.” Temperament research has continued already for more than a century, considering that experiments on properties and types of nervous systems started in Europe in 1906, and a set of temperament models were developed within European psychiatry and American developmental psychology since then. It is a rather parochial position of personality theorists to avoid citation of the findings of these traditions, especially considering that the first models including Emotionality and Activity/Energy dimensions of individuality were developed long before the appearance of the lexical approach (look at the dates: Kant, 1798; Heymanns, 1910–1923). It worth noting that those first models describing these two dimensions targeted an explanation of the nature of Hippocrates' four temperaments. Pavlov's research on temperament, and clinical models of several European psychiatrists were also an attempt to explain the four ancient temperament types. In this sense it would be at least fair to acknowledge that the two main dimensions in the Big Five model (and the only two that showed more or less stability across cultures) were previously described in temperament research. This acknowledgment would of course devalue the novelty of the Big Five model and would suggest that it is in fact temperament and not personality structure that this model describes.

To avoid such devaluation, Big Five papers a) never mention the early two-dimensional models describing four temperament types; b) Eysenk and Gray models are named as models of personality, and the word “temperament” in personality papers seems to be prohibited; c) there are no citations of temperament research in personality journals, whether past or present; d) the word “temperament” is never used for their factors describing biologically based traits; e) whenever an article on temperament comes to a journal of personality it is rejected without review, especially if it does not cite studies using Big Five. You can contact the author for examples.

Personality researchers working within the Big Five seem to have difficulties admitting that they started digging in somebody else's garden and abandoned their own: their subject is the socialization and acculturation processes shaping human individuality, and not biologically based systems of behavioral regulation (temperament). Temperament, similar to sex and age, has nothing to do with socialization, even though, like any other psychological property, temperament interacts with social factors. Also similar to sex and age, temperament relates to neurochemical systems, and social-cultural factors have a rather minimal influence on it. It is wrong therefore to believe that “temperament is almost the same as personality”, or that “temperament is under the umbrella of personality”. We don't put concepts of sex and age under the umbrella of personality, even though studies show personality changes with age, and differences in extraversion between men and women. If we don't mix sex and age with personality, we should not do it with temperament. The differences between personality and temperament lie in the differences between biological factors of individuality and products of socialization. Besides, it we want to pick an umbrella, it is temperament that is the more strong and consistent factor determining the behaviour of an individual, with personality-related socialization developing on the basis of temperament during the life time (i.e. it is personality that should be under the umbrella of temperament). The expression about the “umbrella”, however, is scientifically contra-productive. It mixes two different concepts in one pile, and leads to more confusion. It would be more productive if personality theorists would stop downplaying the concept of temperament and the findings within temperament research and not project their lexical models to biological systems of behavioral regulation.

5 We usually do not substitute the theoretical physics with the maintenance reports of technicians serving the equipment. For the same reason we should not judge the validity of a model of individual differences based on the reports of psychometric properties of self-report tests. After all, biological sciences have more weight in determining what biologically-based traits of individuality exist than psychometric measures of self-reports in regards to these traits. If we want to measure biologically-based systems that make people consistently different, a traditional scientific approach consists of two stages The first stage is to identify the biological systems of individuality, i.e. to partition all the behavioral variance into dimensions that later should determine the scales of our “individuality” test. That is what differential psychology and differential neurophysiology do. At this stage of research it would be natural to derive our partitions based on: a) neuroanatomic studies; b) studies of neurochemical systems regulating human behaviour; c) studies of similar biologically-based traits in other mammals, and d) studies of consistent differences between young children (after all, if we study biologically based characteristics of individuality we need to be sure that they are not a product of social expectations and cultural training).

Only when we have an idea of what can be found in biology that makes people different, can we switch to the second, psychometric, stage in the development of our Individuality test. Luckily the findings in research of types and properties of nervous systems, neurochemistry, neuroanatomy and psychiatry have a lot to offer. The psychometric stage has very little to do with biological investigations and should not substitute for the first stage. Psychometrics is a small area of applied psychology, developing techniques for monitoring the quality of psychological tests. The psychometrist's job is to verify that the items in our test reflect whatever researchers have identified as an important regulatory system; that all items along one scale measure the same trait (without asking the same question all the time); that scales are relatively independent and do not influence each other. For whatever properties we identify at the Research stage we can ask test developers to design a device measuring these properties. If we didn't order the test developers to have items reflecting some important property identified at the Research stage – a scale measuring this property would not appear by itself, and existing items belonging to other scales would reflect the void. If we did put too many items related to the same property – we will receive the strongest and most consistent factor, but it might have nothing to do with how important this property is in real biological systems of individuality. In this sense, if the Research stage is not done properly, the resulting test will measure our object only partially and likely from the wrong angle. For this reason the devices used in medicine are based on principles discovered in biology, clinical and chemical studies of actual life systems, and not on the technical notes of the staff tuning these devices.

In summary, the Research stage results in the description of an object to be measured and the nature of this object, which is relevant to obtain for our goals. The Psychometric stage works on the development and perfection of a measurement device to measure only those aspects of our object that the Research stage pointed to. Yet, I often hear often arguments in favour of lexical approach models of biologically based traits based on psychometric studies, including CFA and EFA of their tests. It is the same difference as the difference between theoretical/experimental physics and engineering. Engineers are the wrong people to ask about what the Universe is made from. Similarly, psychometrists are the wrong people to ask about what fundamental, biologically-based regulatory systems humans have: they can not answer questions on the nature of the object that we measure. They can only answer questions about the measurement device.

6 There is a fundamental conflict of interest between psychometrics and differential psychology: dimensionality vs. functionality. This conflict of interest emerges when conclusions are drawn as to the structure of individuality. Psychometrics seeks out independence of a test's dimensions (scales) and (in the best case scenario) correspondence of test items to some actual elements of behaviour. Psychometrics does not concern itself with the nature of these behavioral elements, only about the correspondence between what is observed and the reading on the measuring device. That's where psychometrics and all techniques based on factor analysis (FA) really lose out: they are incapable of dealing with feedback, and nonlinear and contingent relationships between systems. Yet all life systems (including human individuality) are based on such relationships between their components. These relationships mean interdependency between dimensions, and feedback, nonlinear and contingent mechanisms are described in numerous reports within neuroanatomy and neurochemistry. For this reason functionality (the functional role of regulatory systems) rather than dimensionality should provide the main criteria for describing the structure of natural systems.

If we apply a method designed to search for independent dimensions to systems that have strong interdependency – by definition we will have the wrong picture, even though we are guaranteed, like in gypsy fortune telling, to have some sort of picture. For this reason factor analysis is never considered to be applicable in neurochemical or neuroanatomic models and research. Even outside of psychometrics and te lexical approach, FA is almost useless in the derivation of the taxonomy of objects whose elements have contingent and feedback relationships. By my estimation, I have conducted about 2000 FA protocols just over the past 10 years, within my research on the dimensionality of semantic spaces. Thus I am not as scared of criticising FA as other psychologists might be and to say that it is a very weak investigative tool for research on the structural relationships within natural systems. FA is very dependent on what items you put into its centrifuge, and the more degrees of freedom (diversity, variability) our research object has, the weaker are the resulting factors.

This is the position of a differential psychologist. For psychometrists, however, it is very important that the scales are independent and do not overlap. The quality of the test is measured by the independence of the scales. This is problematic as NONE of the traits or properties ever measured by any psychological test are completely independent from any other psychological property. For this reason there is no such a thing as a perfect test, and psychometrists (i.e. people developing tests) try very hard to brush out this interdependence by creating substructures, smaller facets, hierarchy of factors, etc. It is a hard work, but this does not help to change the fact that FA is completely incapable of reflecting feedback and contingent relationships within the systems under study.

## Supporting Information

Figure S1 **The number of statistically significant sex differences in estimation of given concepts within specific temperament groups: low and high Motor (ERM), Social Endurance (ERS), and high Social Tempo (TMS).** The stacked columns represent the total number of significant differences in the groups of concepts. The colours represent the spectrum of these differences along seven factors to which the scales are associated. The sign indicates the pole of the scales chosen by the male group with the higher scores on a given temperament trait for the given concepts (for example, a positive pole of the scales of Complexity factor is “complex” and a negative pole is “simple”). Female groups with these traits had therefore the opposite patterns of estimations. ERM: Motor Endurance, ERS: Social Endurance, TMS: Social Tempo.
(TIF)

Table S1 **The complete list of the significant** **differences** **in estimations of contrast** **temperament groups, Study 1.** The font alternates between the scales of the Stimulation factor (underlined), Evaluation (normal), **Power** (**bold**), Complexity (***bold italic***), Reality-Probability (normal), *Organization* (*italic*) and Stability-Limitation (normal). Only groups with more than 5 significant differences are shown. Groups of concepts: “People”(Person, Unknown person, My contemporary, Society), “SocialAt” (social attractors: (Prestige, Reputation, Beauty, Freedom), “Reality” (Reality, Present, Life), “Work” (Work, Task, Activity, Effort), “Time” (Time, Speed, Motion, Development), PastFut” (Past, Future). “SimpOr” (Simplicity, Order).
(DOC)

Table S2 **The complete list of the significant differences in estimations of contrast temperament groups, Study 2.** The font alternates between the scales of the Stimulation factor (underlined), Evaluation (normal), **Power** (**bold**), Complexity (***bold italic***), Reality-Probability (normal), *Organization* (*italic*) and Stability-Limitation (normal). The groups of concepts: “People” (Person, Society), “SocialAt” (social attractors: (Prestige, Beauty, Freedom), “WorkRe” (Work, Activity, Reality, Present, Life), “Time” (Time, Speed, Motion, Development), SimpOr (Simplicity, Order, Faith, Relaxation) and “PastFut” (Past, Future).
(DOC)

Table S3 **Significant sex differences in estimations of selected temperament groups.** The font alternates between the scales of the Stimulation factor (underlined), Evaluation (normal), **Power** (**bold**), Complexity (***bold italic***), Reality-Probability (normal), *Organization* (*italic*) and Stability-Limitation (normal). “Work” concepts: Work, Effort, Task, Activity.
(DOC)

Text S1 **Validation summary of the Structure of Temperament Questionnaire.**
(DOC)
