# Supplementary figures and images for: Observer Bias: An Interaction of Temperament Traits with Biases in the Semantic Perception of Lexical Material
Source: PLoS One. 2014 Jan 27;9(1):e85677. doi: 10.1371/journal.pone.0085677 (PMC3903487; doi:10.1371/journal.pone.0085677)

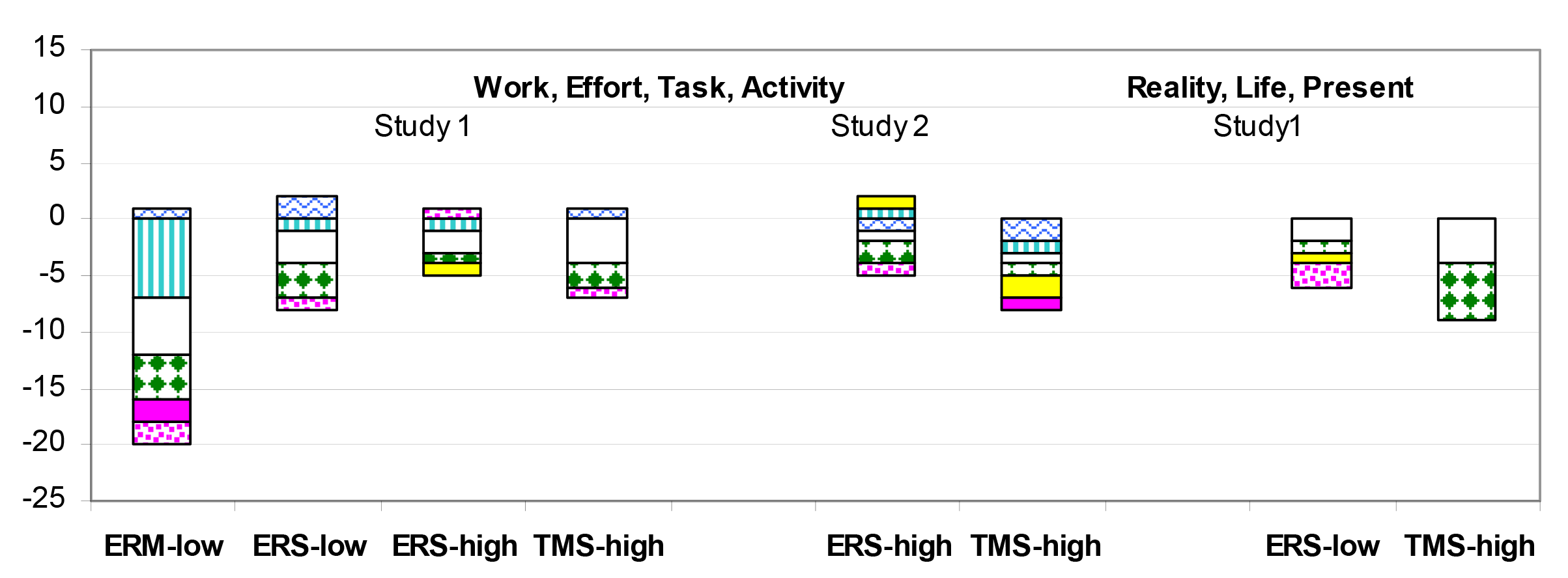

Supplement: Figure S1 — The number of statistically significant sex differences in estimation of given concepts within specific temperament groups: low and high Motor (ERM), Social Endurance (ERS), and high Social Tempo (TMS). The stacked columns represent the total number of significant differences in the groups of concepts. The colours represent the spectrum of these differences along seven factors to which the scales are associated. The sign indicates the pole of the scales chosen by the male group with the higher scores on a given temperament trait for the given concepts (for example, a positive pole of the scales of Complexity factor is “complex” and a negative pole is “simple”). Female groups with these traits had therefore the opposite patterns of estimations. ERM: Motor Endurance, ERS: Social Endurance, TMS: Social Tempo. (TIF) [file pone.0085677.s001.tif]
