# Supplementary material for: Observer Bias: An Interaction of Temperament Traits with Biases in the Semantic Perception of Lexical Material
Source: PLoS One. 2014 Jan 27;9(1):e85677. doi: 10.1371/journal.pone.0085677 (PMC3903487; doi:10.1371/journal.pone.0085677)
Supplement: Table S1 — The complete list of the significant differences in estimations of contrast temperament groups, Study 1. The font alternates between the scales of the Stimulation factor (underlined), Evaluation (normal), Power (bold), Complexity (bold italic), Reality-Probability (normal), Organization (italic) and Stability-Limitation (normal). Only groups with more than 5 significant differences are shown. Groups of concepts: “People”(Person, Unknown person, My contemporary, Society), “SocialAt” (social attractors: (Prestige, Reputation, Beauty, Freedom), “Reality” (Reality, Present, Life), “Work” (Work, Task, Activity, Effort), “Time” (Time, Speed, Motion, Development), PastFut” (Past, Future). “SimpOr” (Simplicity, Order). (DOC) [file pone.0085677.s002.doc]

**Supporting Information, Table S1.** The complete list of the significant differences in estimations of contrast temperament groups, Study 1. The font alternates between the scales of the Stimulation factor (underlined), Evaluation (normal), **Power** (**bold**), Complexity (***bold italic***), Reality-Probability (normal), *Organization* (*italic*) and Stability-Limitation (normal). Only groups with more than 5 significant differences are shown. Groups of concepts: “People”(Person, Unknown person, My contemporary, Society), “SocialAt” (social attractors: (Prestige, Reputation, Beauty, Freedom), “Reality” (Reality, Present, Life), “Work” (Work, Task, Activity, Effort), “Time” (Time, Speed, Motion, Development), PastFut” (Past, Future). “SimpOr” (Simplicity, Order).

| **MEN** with | Z | | | *p*-level | | |  | | **MEN** with | | | Z | | *p*-level | |  | |  |
| --- | --- | --- | --- | --- | --- | --- | --- | --- | --- | --- | --- | --- | --- | --- | --- | --- | --- | --- |
| weaker Motor endurance stronger | | | | | | | | | weaker Motor endurance stronger | | | | | | | | |  |
| estimated **“People”** as more: | | | | | | | | | estimated **“Social attractors”** as more: | | | | | | | | |  |
| draining | 3.98 | | | .0001 | | | stimulat-g | | harmful | | 2.88 | | | .0040 | | useful | |  |
| uninterg | 3.06 | | | .0022 | | | interesting | | ***simple*** | | 3.16 | | | .0016 | | ***complex*** | |  |
| decline | 2.79 | | | .0053 | | | progress | | imaginary | | 2.83 | | | .0047 | | real | |  |
| **weak** | **3.68** | | | **.0002** | | | **powerful** | | impossible | | 2.81 | | | .0050 | | possible | |  |
| ***one-dimen*** | ***2.80*** | | | ***.0051*** | | | ***multi-dim.*** | | *obscure* | | *2.72* | | | *.0065* | | *obvious* | |  |
| artificial | 4.18 | | | .0000 | | | natural | | limited | | 4.33 | | | .0000 | | boundless | |  |
| imaginary | 2.94 | | | .0033 | | | real | | slower Motor tempo faster | | | | | | | | |  |
| *irregular* | *2.87* | | | *.0041* | | | *regular* | | draining | | 2.84 | | | .0046 | | stimulat-g | |  |
| *imprecise* | *3.79* | | | *.0001* | | | *precise* | | **weak** | | 2.89 | | | .0039 | | **powerful** | |  |
| faltering | 3.18 | | | .0015 | | | steady | | artificial | | 2.71 | | | .0067 | | natural | |  |
| fragile | 3.98 | | | .0001 | | | solid | | false | | 3.43 | | | .0006 | | true | |  |
| slower Motor tempo faster | | | | | | | | | impossible | | 3.78 | | | .0002 | | possible | |  |
| unintern-g | 2.87 | | | .0041 | | | interest-g | | inaccessib | | 3.54 | | | .0000 | | accessible | |  |
| irritating | 3.48 | | | .0005 | | | pleasant | | *irrational* | | 4.54 | | | .0000 | | *rational* | |  |
| **following** | **2.77** | | | **.0056** | | | **leading** | | *unreliable* | | 4.07 | | | .0000 | | *reliable* | |  |
| artificial | 3.20 | | | .0014 | | | natural | | faltering | | 3.41 | | | .0007 | | steady | |  |
| false | 3.22 | | | .0013 | | | true | | weaker Social endurance stronger | | | | | | | | |  |
| *unorgan-d* | *2.79* | | | *.0052* | | | *organized* | | uninter-g | | 2.67 | | | .0068 | | interesting | |  |
| faltering | 3.61 | | | .0003 | | | steady | | abundant | | 3.33 | | | .0009 | | popular | |  |
| weaker Social endurance stronger | | | | | | | | | ***difficult*** | | ***3.10*** | | | ***.0019*** | | ***easy*** | |  |
| indifferent | 3.07 | | | .0021 | | | exciting | | artificial | | 3.04 | | | .0024 | | natural | |  |
| irritating | 3.88 | | | .0001 | | | pleasant | | impossible | | 3.15 | | | .0016 | | possible | |  |
| decline | 3.66 | | | .0002 | | | progress | | unusual | | 3.22 | | | .0013 | | ordinary | |  |
| harmful | 2.92 | | | .0035 | | | useful | | *irregular* | | 2.84 | | | .0045 | | *regular* | |  |
| *irrational* | 3.43 | | | .0006 | | | *rational* | | *irrational* | | 3.42 | | | .0006 | | *rational* | |  |
| *steady* | 2.72 | | | .0065 | | | *faltering* | | passive | | 3.08 | | | .0020 | | active | |  |
| scattered | 2.88 | | | .0040 | | | dense | |  | |  | | |  | |  | |  |
| lower Motor emotionality higher | | | | | | | | | lower Motor emotionality higher | | | | | | | | |  |
| *imprecise* | | *3.01* | | | *.0026* | | *precise* | | ***understand*** | | | ***3.80*** | | ***.0001*** | | ***inexplicable*** | |  |
| faltering | | 2.88 | | | .0040 | | steady | | true | | | 2.80 | | .0052 | | false | |  |
| unrestrained | | 3.64 | | | .0003 | | restrained | | existent | | | 2.99 | | .0028 | | imagined | |  |
| lower Social emotionality higher | | | | | | | | | real | | 3.91 | | | .0001 | | imaginary | |  |
| stimulatg | | 3.24 | | | .0012 | | draining | | possible | | | 3.66 | | .0003 | | impossible | |  |
| interesting | | 2.77 | | | .0057 | | uninterng | | known | | | 3.71 | | .0002 | | unknown | |  |
| pleasant | | 3.47 | | | .0005 | | irritating | | inevitable | | | 3.82 | | .0001 | | improbable | |  |
| kind | | 3.41 | | | .0007 | | severe | | ordinary | | | 4.04 | | .0001 | | unusual | |  |
| progress | | 4.42 | | | .0000 | | decline | | *regular* | | | *2.84* | | *.0045* | | *irregular* | |  |
| light | | 3.07 | | | .0021 | | dark | | *rational* | | | *2.73* | | *.0063* | | *irrational* | |  |
| pure | | 3.34 | | | .0008 | | dirty | | *justified* | | | *2.94* | | *.0033* | | *senseless* | |  |
| warm | | 2.81 | | | .0050 | | cold | | *reliable* | | | *2.75* | | *.0060* | | *unreliable* | |  |
| **smooth** | | **2.88** | | | **.0040** | | **rough** | | steady | | | 3.01 | | .0026 | | faltering | |  |
| natural | | 3.28 | | | .0010 | | artificial | | solid | | | 3.41 | | .0006 | | fragile | |  |
| true | 3.67 | | | .0002 | | | false | | lower Social emotionality higher | | | | | | | | |  |
| known | 3.04 | | | .0023 | | | unknown | | known | | 3.95 | | | .0001 | | unknown | |  |
| inevitable | 3.67 | | | .0002 | | | improbab. | | real | | 3.82 | | | .0001 | | imagined | |  |
| *clear* | 3.79 | | | .0002 | | | *blurred* | | possible | | 3.46 | | | .0005 | | impossible | |  |
| *regular* | *3.13* | | | *.0018* | | | *irregular* | | existent | | 3.30 | | | .0010 | | imagined | |  |
| *rational* | *2.86* | | | *.0042* | | | *irrational* | | ***easy*** | | ***3.02*** | | | ***.0025*** | | ***difficult*** | |  |
| *justified* | *3.40* | | | *.0007* | | | *senseless* | | *justified* | | *3.02* | | | *.0026* | | *senseless* | |  |
| stable | 4.02 | | | .0001 | | | unstable | | weaker Social plasticity stronger | | | | | | | | |  |
| steady | 3.61 | | | .0003 | | | faltering | | exciting | | 3.38 | | | .0007 | | indifferent | |  |
| solid | 2.88 | | | .0040 | | | fragile | | pleasant | | 2.71 | | | .0065 | | irritating | |  |
| **WOMEN** of | | | Z | | | *p*level | |  | safe | | | 3.28 | | .0001 | | dangerous | |  |
| weaker Intellectual endurance stronger | | | | | | | | | useful | | 3.66 | | | .0003 | | harmful | |  |
| estimated **“Social attractors”** as more: | | | | | | | | | **significant** | | **4.02** | | | **.0001** | | **insignific** | |  |
| trivial | 2.96 | | | .0030 | | | original | | ***irreplaceab*** | | ***2.61*** | | | ***.0070*** | | ***replaceab.*** | |  |
| ***multidim.*** | ***2.71*** | | | ***.0068*** | | | ***onedim*** | | active | | 2.76 | | | .0005 | | passive | |  |
| typical | 2.98 | | | .0029 | | | atypical | | **WOMEN with** | | | | Z | | *p*level | |  | |
| ordinary | 2.73 | | | .0063 | | | unusual | | weaker Social endurance stronger | | | | | | | | |  |
| *reliable* | *3.03* | | | *.0024* | | | *unreliable* | | estimated **“Social attractors”** as more: | | | | | | | | |  |
| *steady* | *3.73* | | | *.0002* | | | *faltering* | | ***difficult*** | | ***3.99*** | | | ***.0001*** | | ***easy*** | |  |
| fast | 3.08 | | | .0021 | | | slow | | imaginary | | 3.59 | | | .0003 | | real | |  |
|  |  | | |  | | |  | | atypical | | 2.95 | | | .0032 | | typical | |  |
| **MEN** with | | | Z | | | *p*level | |  | real | | | 3.91 | | .0001 | | imaginary | |  |
| weaker Motor endurance stronger | | | | | | | | | atypical | | 2.95 | | | .0032 | | typical | |  |
| estimated **“Reality”** as more: | | | | | | | | | rare | | 4.43 | | | .0000 | | common | |  |
| draining | 3.31 | | | .0009 | | | stimulatg | | unusual | | 3.48 | | | .0005 | | ordinary | |  |
| abundant | 2.89 | | | .0039 | | | popular | | *blurred* | | *3.88* | | | *.0001* | | *clear* | |  |
| rough | 3.21 | | | .0013 | | | smooth | | *irregular* | | *3.32* | | | *.0009* | | *regular* | |  |
| **weak** | **2.97** | | | **.0030** | | | **powerful** | | *unreliable* | | *3.60* | | | *.0003* | | *reliable* | |  |
| impossible | 2.84 | | | .0045 | | | possible | | faltering | | 3.20 | | | .0014 | | steady | |  |
| unknown | 2.69 | | | .0072 | | | known | | lower Social emotionality higher | | | | | | | | |  |
| *blurred* | *3.47* | | | *.0005* | | | *clear* | | exciting | | 2.84 | | | .0045 | | indifferent | |  |
| *irrational* | *3.66* | | | *.0003* | | | *rational* | | stimulatg | | 3.26 | | | .0011 | | draining | |  |
|  |  | | |  | | |  | | useful | | 3.26 | | | .0011 | | harmful | |  |
|  |  | | |  | | |  | | **leading** | | **2.73** | | | **.0063** | | **following** | |  |
|  |  | | |  | | |  | | natural | | 2.83 | | | .0046 | | artificial | |  |
|  |  | | |  | | |  | | possible | | 2.75 | | | .0060 | | impossible | |  |
| weaker Social endurance stronger | | | | | | | | | **WOMEN** of | | | Z | | | *p*level | |  | |
| indifferent | 3.00 | | | .0027 | | | exciting | | weaker Social endurance stronger | | | | | | | | |  |
| indifferent | 3.00 | | | .0027 | | | exciting | | estimated **“Reality”** as more: | | | | | | | | |  |
| pale | 3.11 | | | .0019 | | | bright | | severe | | 4.31 | | | .0000 | | kind | |  |
| draining | 3.63 | | | .0003 | | | stimulatg | | dangerous | | 2.81 | | | .0049 | | safe | |  |
| uninterg | 3.32 | | | .0009 | | | interesting | | dark | | 2.81 | | | .0049 | | light | |  |
| severe | 3.17 | | | .0015 | | | kind | | rough | | 3.35 | | | .0008 | | smooth | |  |
| harmful | 2.83 | | | .0047 | | | useful | | difficult | | 3.01 | | | .0026 | | easy | |  |
| rough | 3.30 | | | .0010 | | | smooth | | **insignific.** | | **2.68** | | | **.0074** | | **significant** | |  |
| **following** | **3.21** | | | **.0013** | | | **leading** | | *blurred* | | *3.62* | | | *.0003* | | *clear* | |  |
| *irrational* | *3.22* | | | *.0013* | | | *rational* | | *irregular* | | *2.90* | | | *.0037* | | *regular* | |  |
| *senseless* | *3.13* | | | *.0018* | | | *justifiable* | | *irrational* | | *3.00* | | | *.0027* | | *rational* | |  |
| *unorgand* | *3.31* | | | *.0009* | | | *organized* | | unstable | | 3.53 | | | .0004 | | stable | |  |
| finite | 3.01 | | | .0026 | | | infinite | | fragile | | 3.81 | | | .0001 | | solid | |  |
| weaker Motor emotionality stronger | | | | | | | | | **MEN of** | | | Z | | | *p*level | |  | |
| *ordered* | *2.66* | | | *.0079* | | | *chaotic* | | weaker Motor endurance stronger | | | | | | | | |  |
| *clear* | *3.12* | | | *.0018* | | | *blurred* | | estimated **“Work”** as more: | | | | | | | | |  |
| *obvious* | *3.16* | | | *.0016* | | | *obscure* | | trivial | | 2.56 | | | .0106 | | original | |  |
| *regular* | *3.41* | | | *.0007* | | | *irregular* | | pale | | 4.61 | | | .0000 | | bright | |  |
| existent | 3.39 | | | .0007 | | | imagined | | uninterg | | 4.66 | | | .0000 | | interesting | |  |
| common | 2.70 | | | .0070 | | | rare | | irritating | | 2.85 | | | .0043 | | pleasant | |  |
| stable | 2.83 | | | .0046 | | | unstable | | decline | | 4.37 | | | .0000 | | progress | |  |
| lower Social emotionality higher | | | | | | | | | dark | | 3.46 | | | .0005 | | light | |  |
| stimulatg | 3.88 | | | .0001 | | | draining | | bad | | 3.11 | | | .0019 | | good | |  |
| interesting | 2.81 | | | .0049 | | | uninterg | | harmful | | 3.01 | | | .0026 | | useful | |  |
| pleasant | 2.92 | | | .0036 | | | irritating | | **weak** | | **2.78** | | | **.0055** | | **powerful** | |  |
| light | 2.66 | | | .0078 | | | dark | | ***discrete*** | | ***3.38*** | | | ***.0007*** | | ***continuous*** | |  |
| useful | 2.69 | | | .0071 | | | harmful | | ***onedim*** | | ***3.83*** | | | ***.0001*** | | ***multidim*** | |  |
| smooth | 2.90 | | | .0038 | | | rough | | artificial | | 3.09 | | | .0020 | | natural | |  |
| **leading** | **3.50** | | | **.0005** | | | **following** | | imaginary | | 3.69 | | | .0002 | | real | |  |
| ***prdered*** | ***3.50*** | | | ***.0005*** | | | ***chaotic*** | | impossible | | 3.76 | | | .0002 | | possible | |  |
| known | 2.93 | | | .0034 | | | unknown | | unknown | | 2.70 | | | .0069 | | known | |  |
| inevitable | 2.77 | | | .0057 | | | improbab. | | *blurred* | | *3.54* | | | *.0004* | | *clear* | |  |
| *clear* | *3.04* | | | *.0024* | | | *blurred* | | *senseless* | | *3.33* | | | *.0009* | | *justified* | |  |
| *justified* | *2.72* | | | *.0066* | | | *senseless* | | *unreliable* | | *3.92* | | | *.0001* | | *reliable* | |  |
| *reliable* | *3.34* | | | *.0008* | | | *unreliable* | | *unorgand* | | *3.00* | | | *.0027* | | *organized* | |  |
| stable | 2.88 | | | .0039 | | | unstable | | unstable | | 2.71 | | | .0068 | | stable | |  |
| steady | 3.37 | | | .0008 | | | faltering | | faltering | | 4.54 | | | .0000 | | steady | |  |
| solid | 2.76 | | | .0059 | | | fragile | | weaker Social endurance stronger | | | | | | | | |  |
| **WOMEN** of | | | Z | | | *p*level | |  | trivial | 2.72 | | | | .0065 | | original | |  |
| weaker Intellectual endurance stronger | | | | | | | | | indifferent | | 3.57 | | | .0004 | | exciting | |  |
| estimated **“Work”** as more: | | | | | | | | | pale | | 3.46 | | | .0005 | | bright | |  |
| original | 3.22 | | | .0013 | | | trivial | | irritating | | 2.76 | | | .0059 | | pleasant | |  |
| sharp | 4.58 | | | .0000 | | | dull | | ***inexplicab*** | | ***3.21*** | | | ***.0013*** | | ***understandb*** | |  |
| **large** | **3.46** | | | **.0005** | | | **small** | | artificial | | 4.09 | | | .0000 | | natural | |  |
| ***chaotic*** | ***4.02*** | | | ***.0001*** | | | ***ordered*** | | false | | 3.35 | | | .0008 | | true | |  |
| ***complex*** | ***4.18*** | | | ***.0000*** | | | ***simple*** | | *irrational* | | *2.85* | | | *.0044* | | *rational* | |  |
| ***difficult*** | ***3.50*** | | | ***.0005*** | | | ***easy*** | | slow | | 3.04 | | | .0023 | | fast | |  |
| improbab. | 3.60 | | | .0003 | | | inevitable | | lower Social plasticity higher | | | | | | | | |  |
| lower Social emotionality higher | | | | | | | | | progress | | 4.52 | | | .0000 | | decline | |  |
| kind | 3.49 | | | .0005 | | | severe | | useful | | 4.04 | | | .0001 | | harmful | |  |
| pure | 3.14 | | | .0017 | | | dirty | | ***continuous*** | | ***2.78*** | | | ***.0055*** | | ***discrete*** | |  |
| **significant** | **3.41** | | | **.0007** | | | **insignific** | | ***understan*** | | ***2.75*** | | | ***.0060*** | | ***inexplicable*** | |  |
| **smooth** | **2.84** | | | **.0045** | | | **rough** | | real | | 4.36 | | | .0000 | | imagined | |  |
| possible | 3.14 | | | .0017 | | | impossible | | *clear* | | *3.50* | | | *.0005* | | *blurred* | |  |
| unrestrd | 3.25 | | | .0012 | | | restrained | | *rational* | | *3.66* | | | *.0003* | | *irrational* | |  |
| **WOMEN** of | | | Z | | | *p*level | |  | *justified* | *2.93* | | | | *.0033* | | *senseless* | |  |
| weaker Intellectual endurance stronger | | | | | | | | | *reliable* | | *3.14* | | | *.0017* | | *unreliable* | |  |
| estimated **“Timing”** as more: | | | | | | | | | *organized* | | *2.87* | | | *.0041* | | *unorgand* | |  |
| original | 3.47 | | | .0005 | | | trivial | | stable | | 2.89 | | | .0039 | | unstable | |  |
| exciting | 2.87 | | | .0041 | | | indifferent | | steady | | 2.91 | | | .0036 | | faltering | |  |
| sharp | 4.20 | | | .0000 | | | dull | | **MEN with** | | | | Z | | *p*level | |  | |
| **powerful** | **3.55** | | | **.0004** | | | **weak** | | weaker Motor endurance stronger | | | | | | | | |  |
| ***chaotic*** | ***3.79*** | | | ***.0002*** | | | ***ordered*** | | estimated **“Timing”** as more: | | | | | | | | |  |
| ***complex*** | ***3.68*** | | | ***.0002*** | | | ***simple*** | | draining | | 3.40 | | | .0007 | | stimulatg | |  |
| *obscure* | *2.73* | | | *.0063* | | | *obvious* | | dull | | 4.00 | | | .0001 | | sharp | |  |
| scattered | 3.28 | | | .0010 | | | dense | | severe | | 3.03 | | | .0025 | | kind | |  |
| lower Social emotionality higher | | | | | | | | | ***inexplicab*** | | ***3.93*** | | | ***.0001*** | | ***understanda*** | |  |
| kind | 3.21 | | | .0013 | | | severe | | artificial | | 2.88 | | | .0039 | | natural | |  |
| pleasant | 2.90 | | | .0038 | | | irritating | | false | | 3.38 | | | .0007 | | true | |  |
| light | 3.23 | | | .0012 | | | dark | | imaginary | | 3.27 | | | .0011 | | real | |  |
| safe | 3.52 | | | .0004 | | | dangerous | | unknown | | 4.79 | | | .0000 | | known | |  |
| light | 2.76 | | | .0058 | | | dark | | rare | | 3.34 | | | .0008 | | common | |  |
| useful | 3.63 | | | .0003 | | | harmful | | unusual | | 2.69 | | | .0071 | | ordinary | |  |
| **significant** | **3.47** | | | **.0005** | | | **insignific.** | | *burred* | | *3.13* | | | *.0017* | | *clear* | |  |
| ***easy*** | ***2.78*** | | | ***.0054*** | | | ***difficult*** | | *irregular* | | *3.42* | | | *.0006* | | *regular* | |  |
| natural | 3.31 | | | .0009 | | | artificial | | *irrational* | | *2.77* | | | *.0055* | | *rational* | |  |
| real | 2.86 | | | .0042 | | | imagined | | *imprecise* | | *3.22* | | | *.0013* | | *precise* | |  |
| common | 3.79 | | | .0001 | | | rare | | faltering | | 3.34 | | | .0008 | | steady | |  |
| *obvious* | *3.22* | | | *.0013* | | | *obscure* | | slower Motor tempo faster | | | | | | | | |  |
| *rational* | *2.69* | | | *.0072* | | | *irrational* | | draining | | | 3.61 | | .0003 | | stimulating | |  |
| infinite | 2.69 | | | .0071 | | | finite | | uninteresg | | | 2.87 | | .0041 | | interesting | |  |
| **WOMEN** of | | | Z | | | *p*level | |  | irritating | | | 3.17 | | .0015 | | pleasant | |  |
| slower Motor tempo faster | | | | | | | | | severe | | | 3.86 | | .0001 | | kind | |  |
| estimated **“Order, Simplicity”** as more: | | | | | | | | | decline | | | 2.83 | | .0046 | | progress | |  |
| calms | 3.31 | | | .0009 | | | arouses | | inexplicable | | | 3.23 | | .0012 | | understandbl | |  |
| pure | 2.75 | | | .0060 | | | dirty | | unknown | | | 3.31 | | .0009 | | known | |  |
| original | 3.45 | | | .0006 | | | trivial | | improbab | | | 3.84 | | .0001 | | inevitable | |  |
| safe | 4.41 | | | .0000 | | | dangerous | | rare | | | 2.78 | | .0055 | | common | |  |
| useful | 3.27 | | | .0011 | | | harmful | | irregular | | | 2.70 | | .0069 | | regular | |  |
| ***ordered*** | ***3.29*** | | | ***.0010*** | | | ***chaotic*** | | *imprecise* | | | *2.70* | | *.0069* | | *precise* | |  |
| ***understan*** | ***3.22*** | | | ***.0013*** | | | ***inexplicb.*** | | *unorgan-d* | | | *3.59* | | *.0003* | | *organized* | |  |
| *rational* | *3.07* | | | *.0021* | | | *irrational* | | slow | | | 2.92 | | .0035 | | fast | |  |
| *justified* | *3.90* | | | *.0001* | | | *senseless* | |  | | |  | |  | |  | |  |
| *organized* | *2.79* | | | *.0053* | | | *unorgand* | |  | | |  | |  | |  | |  |
| **MEN** with | Z | | | *p*level | | |  | | slower Social tempo faster | | | | | | | | |  |
| slower Social tempo faster | | | | | | | | | indifferent | | 3.59 | | | .0003 | | exciting | |  |
| estimated **“Power”** as more: | | | | | | | | | dull | | 3.50 | | | .0005 | | sharp | |  |
| exciting | 3.42 | | | .0006 | | | indifferent | | severe | | 2.91 | | | .0036 | | kind | |  |
| interesting | 3.14 | | | .0017 | | | uninterg | | harmful | | 3.59 | | | .0003 | | useful | |  |
| **deep** | **3.62** | | | **.0003** | | | **superficial** | | superficial | | 3.11 | | | .0019 | | deep | |  |
| **leading** | **3.18** | | | **.0015** | | | **following** | | impossible | | 2.70 | | | .0069 | | possible | |  |
| ***replaceab*** | ***2.90*** | | | ***.003*** | | | ***irreplaceab*** | | *unreliable* | | *3.19* | | | *.0014* | | *reliable* | |  |
| *rational* | *3.11* | | | *.0019* | | | *irrational* | | *unorgand* | | *2.81* | | | *.0049* | | *organized* | |  |
| fast | 3.64 | | | .0003 | | | slow | | soft | | 2.78 | | | .0054 | | rigid | |  |
