# Supplementary material for: Observer Bias: An Interaction of Temperament Traits with Biases in the Semantic Perception of Lexical Material
Source: PLoS One. 2014 Jan 27;9(1):e85677. doi: 10.1371/journal.pone.0085677 (PMC3903487; doi:10.1371/journal.pone.0085677)
Supplement: Table S2 — The complete list of the significant differences in estimations of contrast temperament groups, Study 2. The font alternates between the scales of the Stimulation factor (underlined), Evaluation (normal), Power (bold), Complexity (bold italic), Reality-Probability (normal), Organization (italic) and Stability-Limitation (normal). The groups of concepts: “People” (Person, Society), “SocialAt” (social attractors: (Prestige, Beauty, Freedom), “WorkRe” (Work, Activity, Reality, Present, Life), “Time” (Time, Speed, Motion, Development), SimpOr (Simplicity, Order, Faith, Relaxation) and “PastFut” (Past, Future). (DOC) [file pone.0085677.s003.doc]

**Supporting Information, Table S2.** The complete list of the significant differences in estimations of contrast temperament groups, Study 2. The font alternates between the scales of the Stimulation factor (underlined), Evaluation (normal), **Power** (**bold**), Complexity (***bold italic***), Reality-Probability (normal), *Organization* (*italic*) and Stability-Limitation (normal). The groups of concepts: “People” (Person, Society), “SocialAt” (social attractors: (Prestige, Beauty, Freedom), “WorkRe” (Work, Activity, Reality, Present, Life), “Time” (Time, Speed, Motion, Development), SimpOr (Simplicity, Order, Faith, Relaxation) and “PastFut” (Past, Future).

| **MEN of** | | | | | Z | | | | | | | *p*-level | | | | | | | | | |  | | | **WOMEN of** | | | | | | | | | | | | Z | | | | | *p*-level | | | | | | | | | | | | | | | |  | | | | | | | | | | |  | | | | | |
| --- | --- | --- | --- | --- | --- | --- | --- | --- | --- | --- | --- | --- | --- | --- | --- | --- | --- | --- | --- | --- | --- | --- | --- | --- | --- | --- | --- | --- | --- | --- | --- | --- | --- | --- | --- | --- | --- | --- | --- | --- | --- | --- | --- | --- | --- | --- | --- | --- | --- | --- | --- | --- | --- | --- | --- | --- | --- | --- | --- | --- | --- | --- | --- | --- | --- | --- | --- | --- | --- | --- | --- | --- | --- | --- |
| weaker Motor endurance stronger | | | | | | | | | | | | | | | | | | | | | | | | | weaker Motor endurance stronger | | | | | | | | | | | | | | | | | | | | | | | | | | | | | | | | | | | | | | | | | | | |  | | | | | |
| estimated **“People”** as more: | | | | | | | | | | | | | | | | | | | | | | | | | estimated **“Social attractors”** as more: | | | | | | | | | | | | | | | | | | | | | | | | | | | | | | | | | | | | | | | | | | | |  | | | | | |
| uninteresting | | | | | 3.08 | | | | | | | .0021 | | | | | interesting | | | | | | | | pale | | | | 3.22 | | | | | | | | | | | | | .0013 | | | | | | | | | | | | bright | | | | | | | | | | | | | | |  | | | | | |
| irritating | | | | | 2.74 | | | | | | | .0061 | | | | | pleasant | | | | | | | | irritating | | | | 2.85 | | | | | | | | | | | | | .0043 | | | | | | | | | | | | pleasant | | | | | | | | | | | | | | |  | | | | | |
| **weak** | | | | | **3.17** | | | | | | | **.0015** | | | | | **powerful** | | | | | | | | severe | | | | 2.87 | | | | | | | | | | | | | .0041 | | | | | | | | | | | | kind | | | | | | | | | | | | | | |  | | | | | |
| uniform | | | | | 3.11 | | | | | | | .0018 | | | | | diverse | | | | | | | | dangerous | | | | 3.00 | | | | | | | | | | | | | .0027 | | | | | | | | | | | | safe | | | | | | | | | | | | | | |  | | | | | |
| artificial | | | | | 2.93 | | | | | | | .0033 | | | | | natural | | | | | | | | **following** | | | | **3.68** | | | | | | | | | | | | | **.0002** | | | | | | | | | | | | **leading** | | | | | | | | | | | | | | |  | | | | | |
| faltering | | | | | 3.94 | | | | | | | .0001 | | | | | steady | | | | | | | | ***one-dimen*** | | | | ***4.45*** | | | | | | | | | | | | | ***.0000*** | | | | | | | | | | | | ***multi-dim*** | | | | | | | | | | | | | | |  | | | | | |
| slower Motor Tempo faster | | | | | | | | | | | | | | | | | | | | | | | | | ***replaceable*** | | | | ***2.69*** | | | | | | | | | | | | | ***.0071*** | | | | | | | | | | | | ***irreplaceab*** | | | | | | | | | | | | | | |  | | | | | |
| irritating | | | | | 3.07 | | | | | | | .0021 | | | | | pleasant | | | | | | | | limited | | | | 3.17 | | | | | | | | | | | | | .0015 | | | | | | | | | | | | boundless | | | | | | | | | | | | | | |  | | | | | |
| false | | 2.82 | | | | | | | .0048 | | | | | | | | | | | true | | | | | weaker Social endurance stronger | | | | | | | | | | | | | | | | | | | | | | | | | | | | | | | | | | | | | | | | | | | |  | | | | | |
| **weak** | | | | | **2.69** | | | | | | | **.0072** | | | | | **powerful** | | | | | | | | indifferent | | | | 3.45 | | | | | | | | | | | | | .0006 | | | | | | | | | | | | exciting | | | | | | | | | | | | | | |  | | | | | |
| ***one-dimen*** | | | | | ***2.71*** | | | | | | | ***.0067*** | | | | | ***multi-dim*** | | | | | | | | pale | | | | 3.48 | | | | | | | | | | | | | .0005 | | | | | | | | | | | | bright | | | | | | | | | | | | | | |  | | | | | |
| finite | | | | | 3.09 | | | | | | | .0019 | | | | | infinite | | | | | | | | uninterest-g | | | | 2.75 | | | | | | | | | | | | | .0060 | | | | | | | | | | | | interesting | | | | | | | | | | | | | | |  | | | | | |
| lower Neuroticism higher | | | | | | | | | | | | | | | | | | | | | | | | | dull | | | | | | | 3.64 | | | | | | | | | | | | | .0003 | | | | | | | | | | | sharp | | | | | | | | | | | | |  | | | | | |
| progress | | | | | 2.89 | | | | | | | .0038 | | | | | decline | | | | | | | | dirty | | | | 2.84 | | | | | | | | | | | | | .0045 | | | | | | | | | | | | pure | | | | | | | | | | | | | | |  | | | | | |
| light | | | | | 2.78 | | | | | | | .0054 | | | | | dark | | | | | | | | harmful | | | | -2.86 | | | | | | | | | | | | | .0043 | | | | | | | | | | | | useful | | | | | | | | | | | | | | |  | | | | | |
| *clear* | *3.30* | | | | | | | | | | | *.0010* | | | | | *blurred* | | | | | | | | **insignific.** | | | | **3.03** | | | | | | | | | | | | | **.0025** | | | | | | | | | | | | **signific-t** | | | | | | | | | | | |  | | | | | | | | |
| *obvious* | *3.08* | | | | | | | | | | | *.0021* | | | | | *obscure* | | | | | | | | **small** | | | | **2.93** | | | | | | | | | | | | | **.0034** | | | | | | | | | | | | **large** | | | | | | | | | | | |  | | | | | | | | |
| stable | 3.65 | | | | | | | | | | | .0003 | | | | | unstable | | | | | | | | imagined | | | | 2.74 | | | | | | | | | | | | | .0062 | | | | | | | | | | | | existent | | | | | | | | | | | | | | | |  | | | | |
| **MEN of** | | | | | | | | Z | | | | | | | *p*-level | | | | | | | | |  | imaginary | | | | | | | | | | 4.06 | | | | | | | | | | .0000 | | | | | | | | | | | real | | | | | | | | | | |  | | | | | | | |
| weaker Social endurance stronger | | | | | | | | | | | | | | | | | | | | | | | | | impossible | | | | 3.30 | | | | | | | | | | | | | .0010 | | | | | | | | | | | | possible | | | | | | | | | | | |  | | | | | | | | |
| estimated **“Social attractors”** as more: | | | | | | | | | | | | | | | | | | | | | | | | | atypical | | | | 4.63 | | | | | | | | | | | | | .0000 | | | | | | | | | | | | typical | | | | | | | | | | | | |  | | | | | | | |
| dull | | | | | 3.01 | | | | | | | .0026 | | | | | sharp | | | | | | | | rare | | | | 3.29 | | | | | | | | | | | | | .0010 | | | | | | | | | | | | common | | | | | | | | | | | | |  | | | | | | | |
| abundant | | | | | 3.36 | | | | | | | .0008 | | | | | popular | | | | | | | | *blurred* | | | | *3.19* | | | | | | | | | | | | | *.0014* | | | | | | | | | | | | *clear* | | | | | | | | | | | | |  | | | | | | | |
| decline | | | | | 3.22 | | | | | | | .0013 | | | | | progress | | | | | | | | *irregular* | | | | *3.19* | | | | | | | | | | | | | *.0014* | | | | | | | | | | | | *regular* | | | | | | | | | | | | |  | | | | | | | |
| **insignific.** | | | | | **3.45** | | | | | | | **.0006** | | | | | **significant** | | | | | | | | slow | | | | 2.88 | | | | | | | | | | | | | .0040 | | | | | | | | | | | | fast | | | | | | | | | | | | |  | | | | | | | |
| **following** | | | | | **2.92** | | | | | | | **.0036** | | | | | **leading** | | | | | | | | weaker Intellectual endurance stronger | | | | | | | | | | | | | | | | | | | | | | | | | | | | | | | | | | | | | | | | | | | |  | | | | | |
| **dependent** | | | | | **2.99** | | | | | | | **.0028** | | | | | **independ.** | | | | | | | | exciting | | | | 3.43 | | | | | | | | | | | | | .0006 | | | | | | | | | | | | indifferent | | | | | | | | | | | | |  | | | | | | | |
| imagined | | | | | 2.86 | | | | | | | .0043 | | | | | existent | | | | | | | | bright | | | | 3.19 | | | | | | | | | | | | | .0014 | | | | | | | | | | | | pale | | | | | | | | | | | | |  | | | | | | | |
| *blurred* | | | | | *3.21* | | | | | | | *.0013* | | | | | *clear* | | | | | | | | stimulating | | | | 2.90 | | | | | | | | | | | | | .0038 | | | | | | | | | | | | draining | | | | | | | | | | | | |  | | | | | | | |
| *irregular* | | | | | *3.47* | | | | | | | *.0005* | | | | | *regular* | | | | | | | | kind | | | | -2.82 | | | | | | | | | | | | | .0048 | | | | | | | | | | | | severe | | | | | | | | | | | | |  | | | | | | | |
| *irrational* | | | | | *2.96* | | | | | | | *.0031* | | | | | *rational* | | | | | | | | safe | | | | 2.73 | | | | | | | | | | | | | .0063 | | | | | | | | | | | | dangerous | | | | | | | | | | | | |  | | | | | | | |
| *imprecise* | | | | | *3.63* | | | | | | | *.0003* | | | | | *precise* | | | | | | | | **deep** | | | | **3.22** | | | | | | | | | | | | | **.0013** | | | | | | | | | | | | **superficial** | | | | | | | | | | | | |  | | | | | | | |
| *unorganized* | | | | | *4.48* | | | | | | | *.0000* | | | | | *organized* | | | | | | | | *regular* | | | | *3.38* | | | | | | | | | | | | | *.0007* | | | | | | | | | | | | | | | | *irregular* | | | | | | | | | | |  | | | | | |
| unstable | | | | | 3.22 | | | | | | | .0013 | | | | | stable | | | | | | | | *rational* | | | | *2.92* | | | | | | | | | | | | | *.0035* | | | | | | | | | | | | | | | | *irrational* | | | | | | | | | | |  | | | | | |
| fragile | | | | | 3.24 | | | | | | | .0012 | | | | | solid | | | | | | | | *precise* | | | | *4.02* | | | | | | | | | | | | | *.0001* | | | | | | | | | | | | | | | | *imprecise* | | | | | | | | | | |  | | | | | |
| lower Self-confidence higher | | | | | | | | | | | | | | | | | | | | | | | | | *organized* | | | | *3.25* | | | | | | | | | | | | | *.0011* | | | | | | | | | | | | | | | | *unorgan-d* | | | | | | | | | | |  | | | | | |
| ***multi-dimen*** | | | | | ***2.74*** | | | | | | | ***.0061*** | | | | | ***one-dimen*** | | | | | | | | stable | | | | 3.58 | | | | | | | | | | | | | .0003 | | | | | | | | | | | | | | | | unstable | | | | | | | | | | |  | | | | | |
| false | | | | | 2.83 | | | | | | | .0047 | | | | | true | | | | | | | | **WOMEN of** | | | | | | | | | | | | Z | | | | | | | | | | *p*-level | | | | | | | | | | | | | | |  | | | | | | | | | | | | |
| unusual | | | | | 2.84 | | | | | | | .0045 | | | | | ordinary | | | | | | | | weaker Social endurance stronger | | | | | | | | | | | | | | | | | | | | | | | | | | | | | | | | | | | | | | | | | | | |  | | | | | |
| *obscure* | | | | | *3.20* | | | | | | | *.0014* | | | | | *obvious* | | | | | | | | estimated **Past, History** as more: | | | | | | | | | | | | | | | | | | | | | | | | | | | | | | | | | | | | | | | | | | | |  | | | | | |
| *unorganized* | | | | | *2.88* | | | | | | | *.0040* | | | | | *organized* | | | | | | | | indifferent | | | | 3.96 | | | | | | | | | | | | | .0001 | | | | | | | | | | | | | | | | exciting | | | | | | | | | | |  | | | | | |
| boundless | | | | | 2.99 | | | | | | | .0028 | | | | | limited | | | | | | | | dull | | | | 3.42 | | | | | | | | | | | | | .0006 | | | | | | | | | | | | | | | | sharp | | | | | | | | | | |  | | | | | |
| infinite | | | | | 3.04 | | | | | | | .0023 | | | | | finite | | | | | | | | harmful | | | | 2.83 | | | | | | | | | | | | | .0047 | | | | | | | | | | | | | | | | useful | | | | | | | | | | |  | | | | | |
| lower Sensation Seeking higher | | | | | | | | | | | | | | | | | | | | | | | | | **insignificant** | | | | **3.77** | | | | | | | | | | | | | **.0002** | | | | | | | | | | | | | | | | **significant** | | | | | | | | | | |  | | | | | |
| progress | | | | | 3.23 | | | | | | | .0012 | | | | | decline | | | | | | | | **small** | | | | **3.12** | | | | | | | | | | | | | **.0000** | | | | | | | | | | | | | | | | **large** | | | | | | | | | | |  | | | | | |
| **leading** | | | | | **3.26** | | | | | | | **.0011** | | | | | **following** | | | | | | | | **weak** | | | | **3.73** | | | | | | | | | | | | | **.0002** | | | | | | | | | | | | | | | | **powerful** | | | | | | | | | | |  | | | | | |
| true | | | | | 3.27 | | | | | | | .0011 | | | | | false | | | | | | | | bright | | | | 3.03 | | | | | | | | | | | | | .0025 | | | | | | | | | | | | | | | | pale | | | | | | | | | | |  | | | | | |
| ordinary | | | | | 4.70 | | | | | | | .0000 | | | | | unusual | | | | | | | | *irrational* | | | | *2.86* | | | | | | | | | | | | | *.0043* | | | | | | | | | | | | | | | | *rational* | | | | | | | | | | |  | | | | | |
| *justified* | | | | | *3.59* | | | | | | | *.0003* | | | | | *senseless* | | | | | | | |  |  | | | | | | | | | | | | |  | | | | | | | | | | | | |  | | | | | | | | | | | | | | | | |  | | | | | |
| *organized* | | | | | *3.08* | | | | | | | *.0021* | | | | | *unorganized* | | | | | | | | **WOMEN of** | | | | | | | | | | | | Z | | | | | | | | | | *p*-level | | | | | | | | | | | | | | |  | | | | | | | | | | | | |
| *understandb* | | | | | *4.12* | | | | | | | *.0000* | | | | | *inexplicable* | | | | | | | | weaker Social endurance stronger | | | | | | | | | | | | | | | | | | | | | | | | | | | | | | | | | | | | | | | | | | | |  | | | | | |
| solid | | | | | 4.31 | | | | | | | .0000 | | | | | fragile | | | | | | | | estimated **“WorkReality”** as more: | | | | | | | | | | | | | | | | | | | | | | | | | | | | | | | | | | | | | | | | | | | |  | | | | | |
| active | | | | | 2.76 | | | | | | | .0058 | | | | | passive | | | | | | | | draining | 3.10 | | | | | | | | | | | | | .0019 | | | | | | | | | | | | | stimulat-g | | | | | | | | | | | | | | | | |  | | | | | |
| lower Sens. to Probabilities higher | | | | | | | | | | | | | | | | | | | | | | | | | uninterest-g | 3.81 | | | | | | | | | | | | | .0001 | | | | | | | | | | | | | interesting | | | | | | | | | | | | | | | | |  | | | | | |
| kind | | | | | 3.46 | | | | | | | .0005 | | | | | severe | | | | | | | | dull | 2.88 | | | | | | | | | | | | | .0039 | | | | | | | | | | | | | sharp | | | | | | | | | | | | | | | | |  | | | | | |
| good | | | | | 3.00 | | | | | | | .0027 | | | | | bad | | | | | | | | irritating | 3.07 | | | | | | | | | | | | | .0021 | | | | | | | | | | | | | pleasant | | | | | | | | | | | | | | | | |  | | | | | |
| ***simple*** | | | | | **2.61** | | | | | | | **.0091** | | | | | ***complex*** | | | | | | | | severe | 3.68 | | | | | | | | | | | | | .0002 | | | | | | | | | | | | | kind | | | | | | | | | | | | | | | | |  | | | | | |
| known | | | | | 3.17 | | | | | | | .0015 | | | | | unknown | | | | | | | | harmful | 2.71 | | | | | | | | | | | | | .0067 | | | | | | | | | | | | | useful | | | | | | | | | | | | | | | | |  | | | | | |
| existent | | | | | 3.96 | | | | | | | .0001 | | | | | imagined | | | | | | | | cold | 2.84 | | | | | | | | | | | | | .0043 | | | | | | | | | | | | | warm | | | | | | | | | | | | | | | | |  | | | | | |
| common | | | | | 4.61 | | | | | | | .0000 | | | | | rare | | | | | | | | decline | 3.15 | | | | | | | | | | | | | .0016 | | | | | | | | | | | | | progress | | | | | | | | | | | | | | | | |  | | | | | |
| typical | | | | | 3.60 | | | | | | | .0003 | | | | | atypical | | | | | | | | dark | 3.03 | | | | | | | | | | | | | .0025 | | | | | | | | | | | | | light | | | | | | | | | | | | | | | | |  | | | | | |
| **MEN of** | | | | | | | | Z | | | | | | | *p*-level | | | | | | | | |  | rough | 2.62 | | | | | | | | | | | | | .0068 | | | | | | | | | | | | | smooth | | | | | | | | | | | | | | | | | | | |  | | |
| lower Social endurance higher | | | | | | | | | | | | | | | | | | | | | | | | | **insignific** | **4.13** | | | | | | | | | | | | | **.0000** | | | | | | | | | | | | | **significant** | | | | | | | | | | | | | | | | |  | | | | | |
| estimated **“WorkReality”** as more: | | | | | | | | | | | | | | | | | | | | | | | | | artificial | 2.84 | | | | | | | | | | | | | .0044 | | | | | | | | | | | | | natural | | | | | | | | | | | | | | | | |  | | | | | |
| indifferent | | | | | 2.92 | | | | | | | .0035 | | | | | exciting | | | | | | | | imagined | 2.73 | | | | | | | | | | | | | .0063 | | | | | | | | | | | | | existent | | | | | | | | | | | | | | | | |  | | | | | |
| uninteresting | | | | | 3.12 | | | | | | | .0018 | | | | | interesting | | | | | | | | imaginary | 3.05 | | | | | | | | | | | | | .0022 | | | | | | | | | | | | | real | | | | | | | | | | | | | | | | |  | | | | | |
| abundant | | | | | 3.39 | | | | | | | .0007 | | | | | popular | | | | | | | | impossible | | | | 3.68 | | | | | | | | | | | | | .0002 | | | | | | | | | | | | | | | | possible | | | | | | | | | | |  | | | | | |
| severe | | | | | 3.42 | | | | | | | .0006 | | | | | kind | | | | | | | | *senseless* | | | | *2.94* | | | | | | | | | | | | | *.0032* | | | | | | | | | | | | | | | | *justified* | | | | | | | | | | |  | | | | | |
| bad | | | | | 3.12 | | | | | | | .0018 | | | | | good | | | | | | | | faltering | | | | 3.23 | | | | | | | | | | | | | .0012 | | | | | | | | | | | | | | | | steady | | | | | | | | | | |  | | | | | |
| *irrational* | | | | | *3.08* | | | | | | | *.0021* | | | | | *rational* | | | | | | | |  | | | |  | | | | | | | | | | | | |  | | | | | | | | | | | | | | | |  | | | | | | | | | | |  | | | | | |
| lower Empathy higher | | | | | | | | | | | | | | | | | | | | | | | | | lower Empathy higher | | | | | | | | | | | | | | | | | | | | | | | | | | | | | | | | | | | | | | | | | | | | |  | | | | |
| inevitable | | | | | 3.08 | | | | | | | .0020 | | | | | improbable | | | | | | | | interesting | | | | | 2.83 | | | | | | | | | | | | | .0046 | | | | | | | | | | | | | | | | uninteres-g | | | | | | | | | | |  | | | | |
| *justified* | | | | | *2.77* | | | | | | | *.0054* | | | | | *senseless* | | | | | | | | **massive** | | | | | **2.90** | | | | | | | | | | | | | **.0037** | | | | | | | | | | | | | | | | **delicate** | | | | | | | | | | |  | | | | |
| *reliable* | | | | | *4.12* | | | | | | | *.0000* | | | | | *unreliable* | | | | | | | | **large** | | | | | **3.40** | | | | | | | | | | | | | **.0007** | | | | | | | | | | | | | | | | **small** | | | | | | | | | | |  | | | | |
| stable | | | | | 3.54 | | | | | | | .0004 | | | | | unstable | | | | | | | | ***multi-dimen*** | | | | | ***2.93*** | | | | | | | | | | | | | ***.0033*** | | | | | | | | | | | | | | | | ***one-dimen*** | | | | | | | | | | |  | | | | |
| steady | | | | | 3.01 | | | | | | | .0026 | | | | | faltering | | | | | | | | existent | | | | | 2.99 | | | | | | | | | | | | | .0027 | | | | | | | | | | | | | | | | imagined | | | | | | | | | | |  | | | | |
| constant | | | | | 4.08 | | | | | | | .0000 | | | | | changeable | | | | | | | | common | | | | | 3.83 | | | | | | | | | | | | | .0001 | | | | | | | | | | | | | | | | rare | | | | | | | | | | |  | | | | |
| solid | | | | | 2.81 | | | | | | | .0048 | | | | | fragile | | | | | | | | possible | | | | | 3.07 | | | | | | | | | | | | | .0021 | | | | | | | | | | | | | | | | impossible | | | | | | | | | | |  | | | | |
| lower Sensation Seeking higher | | | | | | | | | | | | | | | | | | | | | | | | | lower Neuroticism higher | | | | | | | | | | | | | | | | | | | | | | | | | | | | | | | | | | | | | | | | | | | | |  | | | | |
| *justified* | | | | | 2.65 | | | | | | | .0081 | | | | | *senseless* | | | | | | | | exciting | 3.19 | | | | | | | | | | | | | .0014 | | | | | | | | | | | | | indifferent | | | | | | | | | | | | | | | | |  | | | | | |
| *organized* | | | | | 2.76 | | | | | | | .0057 | | | | | *unorganized* | | | | | | | | bright | 3.08 | | | | | | | | | | | | | .0020 | | | | | | | | | | | | | pale | | | | | | | | | | | | | | | | |  | | | | | |
| *understandb* | | | | | 2.89 | | | | | | | .0039 | | | | | *inexplicable* | | | | | | | | interesting | 4.54 | | | | | | | | | | | | | .0000 | | | | | | | | | | | | | uninter-g | | | | | | | | | | | | | | | | |  | | | | | |
| decline | | | | | 3.35 | | | | | | | .0008 | | | | | progress | | | | | | | | **significant** | **3.63** | | | | | | | | | | | | | **.0003** | | | | | | | | | | | | | **insignific.** | | | | | | | | | | | | | | | | |  | | | | | |
| *irrational* | | | | | 3.65 | | | | | | | .0003 | | | | | *rational* | | | | | | | | ***continuous*** | ***3.01*** | | | | | | | | | | | | | ***.0027*** | | | | | | | | | | | | | ***discrete*** | | | | | | | | | | | | | | | | |  | | | | | |
| unrestrained | | | | | 2.68 | | | | | | | .0074 | | | | | restrained | | | | | | | | possible | 2.89 | | | | | | | | | | | | | .0038 | | | | | | | | | | | | | impossible | | | | | | | | | | | | | | | | |  | | | | | |
| lower Motor endurance higher | | | | | | | | | | | | | | | | | | | | | | | | | *justified* | | *2.83* | | | | | | | | | | | | | *.0045* | | | | | | | | | | | | | *senseless* | | | | | | | | | | | | | | | | |  | | | | |
| draining | | | | | 3.42 | | | | | | | .0006 | | | | | stimulat-g | | | | | | | | **MEN of** | | | | | | | | | | | | Z | | | | | | | | | | *p*-level | | | | | | | | | | | | | | |  | | | | | | | | | | | | |
| abundant | | | | | 3.26 | | | | | | | .0011 | | | | | popular | | | | | | | | slower Motor Tempo faster | | | | | | | | | | | | | | | | | | | | | | | | | | | | | | | | | | | | | | | | | | | |  | | | | | |
| dirty | | | | | 2.72 | | | | | | | .0065 | | | | | pure | | | | | | | | estimated **“WorkReality”** as more: | | | | | | | | | | | | | | | | | | | | | | | | | | | | | | | | | | | | | | | | | | | |  | | | | | |
| bad | | | | | 2.95 | | | | | | | .0031 | | | | | good | | | | | | | | indifferent | 3.66 | | | | | | | | | | | | | .0003 | | | | | | | | | | | | | exciting | | | | | | | | | | | | | | | | |  | | | | | |
| **weak** | | | | | **3.07** | | | | | | | **.0021** | | | | | **powerful** | | | | | | | | bad | 3.51 | | | | | | | | | | | | | .0005 | | | | | | | | | | | | | good | | | | | | | | | | | | | | | | |  | | | | | |
| impossible | | | | | 2.76 | | | | | | | .0057 | | | | | possible | | | | | | | | **weak** | **4.06** | | | | | | | | | | | | | **.0000** | | | | | | | | | | | | | **powerful** | | | | | | | | | | | | | | | | |  | | | | | |
|  | | | | |  | | | | | | |  | | | | |  | | | | | | | | imaginary | 2.61 | | | | | | | | | | | | | .0090 | | | | | | | | | | | | | real | | | | | | | | | | | | | | | | |  | | | | | |
|  | | | | |  | | | | | | |  | | | | |  | | | | | | | | unrestrained | | | | 3.03 | | | | | | | | | | | | | .0025 | | | | | | | | | | | | | | | | restrained | | | | | | | | | | |  | | | | | |
| **MEN of** | | | | | | Z | | | | | | | *p*-level | | | | | | |  | | | | | **WOMEN of** | | | | | | | | | | | | | | | | Z | | | | | | | | | *p*-level | | | | | | | | | | | | | | |  | | | | |  | | | | |
| slower Motor tempo faster | | | | | | | | | | | | | | | | | | | | | | | | | weaker Social endurance stronger | | | | | | | | | | | | | | | | | | | | | | | | | | | | | | | | | | | | | | | | | | | | |  | | | | |
| estimated **“Timing”** as more: | | | | | | | | | | | | | | | | | | | | | | | | | estimated **“Timing”** as more: | | | | | | | | | | | | | | | | | | | | | | | | | | | | | | | | | | | | | | | | | | | | |  | | | | |
| draining | | | | | 4.17 | | | | | | | .0000 | | | | | stimulat-g | | | | | | | | irritating | | | | | 3.17 | | | | | | | | | | | | | .0015 | | | | | | | | | | | | | | | | pleasant | | | | | | | | | | |  | | | | |
| slow | | | | | 3.77 | | | | | | | .0001 | | | | | fast | | | | | | | | cold | | | | | 3.03 | | | | | | | | | | | | | .0024 | | | | | | | | | | | | | | | | warm | | | | | | | | | | |  | | | | |
| irritating | | | | | 2.80 | | | | | | | .0051 | | | | | pleasant | | | | | | | | severe | | | | | 2.70 | | | | | | | | | | | | | .0069 | | | | | | | | | | | | | | | | kind | | | | | | | | | | |  | | | | |
| severe | | | | | 3.41 | | | | | | | .0006 | | | | | kind | | | | | | | | **weak** | | | | | **3.02** | | | | | | | | | | | | | **.0025** | | | | | | | | | | | | | | | | **powerful** | | | | | | | | | | |  | | | | |
| cold | | | | | 2.87 | | | | | | | .0040 | | | | | warm | | | | | | | | false | | | | | 2.74 | | | | | | | | | | | | | .0060 | | | | | | | | | | | | | | | | true | | | | | | | | | | |  | | | | |
| bad | | | | | 3.34 | | | | | | | .0008 | | | | | good | | | | | | | | imagined | | | | | 3.25 | | | | | | | | | | | | | .0012 | | | | | | | | | | | | | | | | existent | | | | | | | | | | |  | | | | |
| **weak** | | | | | **2.93** | | | | | | | **.0034** | | | | | **powerful** | | | | | | | | impossible | | | | | 3.18 | | | | | | | | | | | | | .0014 | | | | | | | | | | | | | | | | possible | | | | | | | | | | |  | | | | |
| **insignific.** | | | | | **2.76** | | | | | | | **.0058** | | | | | **significant** | | | | | | | | *blurred* | | | | | *3.38* | | | | | | | | | | | | | *.0007* | | | | | | | | | | | | | | | | *clear* | | | | | | | | | | |  | | | | |
| ***one-dim*** | | | | | ***3.12*** | | | | | | | ***.0018*** | | | | | ***multi-dim*** | | | | | | | | *imprecise* | | | | | *3.06* | | | | | | | | | | | | | *.0022* | | | | | | | | | | | | | | | | *precise* | | | | | | | | | | |  | | | | |
| impossible | | | | | 3.46 | | | | | | | .0005 | | | | | possible | | | | | | | | faltering | | | | | 2.71 | | | | | | | | | | | | | .0066 | | | | | | | | | | | | | | | | steady | | | | | | | | | | |  | | | | |
| improbable | | | | | 4.09 | | | | | | | .0000 | | | | | inevitable | | | | | | | | unstable | | | | | 3.18 | | | | | | | | | | | | | .0014 | | | | | | | | | | | | | | | | stable | | | | | | | | | | |  | | | | |
| rare | | | | | 3.30 | | | | | | | .0009 | | | | | common | | | | | | | | fragile | | | | | 3.51 | | | | | | | | | | | | | .0004 | | | | | | | | | | | | | | | | solid | | | | | | | | | | |  | | | | |
| imagined | | | | | 2.91 | | | | | | | .0036 | | | | | existent | | | | | | | |  | | | | |  | | | | | | | | | | | | |  | | | | | | | | | | | | | | | |  | | | | | | | | | | |  | | | | |
| *imprecise* | | | | | *2.80* | | | | | | | *.0051* | | | | | *precise* | | | | | | | |  | | | | |  | | | | | | | | | | | | |  | | | | | | | | | | | | | | | |  | | | | | | | | | | |  | | | | |
| *unreliable* | | | | | *3.25* | | | | | | | *.0011* | | | | | *reliable* | | | | | | | |  | | | | |  | | | | | | | | | | | | |  | | | | | | | | | | | | | | | |  | | | | | | | | | | |  | | | | |
| lower Sensit. to probabilities higher | | | | | | | | | | | | | | | | | | | | | | | | | lower Neuroticism higher | | | | | | | | | | | | | | | | | | | | | | | | | | | | | | | | | | | | | | | | | | | | |  | | | | |
| good | | | | | 4.11 | | | | | .0000 | | | | | | | | bad | | | | | | | exciting | | | | | | 3.56 | | | | | | | | | | | | | .0004 | | | | | | | | | | | | | | | | indifferent | | | | | | | | | | |  | | | |
| warm | | | | | 2.99 | | | | | .0027 | | | | | | | | cold | | | | | | | **significant** | | | | | | **2.77** | | | | | | | | | | | | | **.0057** | | | | | | | | | | | | | | | | **insignific.** | | | | | | | | | | |  | | | |
| **significant** | | | | | **3.41** | | | | | **.0006** | | | | | | | | **insignific.** | | | | | | | **large** | | | | | | **3.50** | | | | | | | | | | | | | **.0004** | | | | | | | | | | | | | | | | **small** | | | | | | | | | | |  | | | |
| existent | | | | | | 4.65 | | | | | .0000 | | | | | | | imagined | | | | | | | possible | | | | | | | | 2.77 | | | | | | | | | | | | | .0057 | | | | | | | | | | | imposs. | | | | | | | | | | |  | | | | | | |
| known | | | | | | 5.19 | | | | | .0000 | | | | | | | unknown | | | | | | | dense | | | | | | | | 3.56 | | | | | | | | | | | | | .0004 | | | | | | | | | | | scattered | | | | | | | | | | |  | | | | | | |
| ordinary | | | | | | 2.71 | | | | | .0067 | | | | | | | unusual | | | | | | | infinite | | | | | | | | 3.11 | | | | | | | | | | | | | .0019 | | | | | | | | | | | finite | | | | | | | | | | |  | | | | | | |
| typical | | | | | 5.04 | | | | | .0000 | | | | | | | | atypical | | | | | | | **WOMEN of** | | | | | | | | | | | | | Z | | | | | | | | | | *p*-level | | | | | | | | | | | | | | |  | | | | | | | | | |  | |
| common | | | | | 4.42 | | | | | .0000 | | | | | | | | rare | | | | | | | weaker Social endurance stronger | | | | | | | | | | | | | | | | | | | | | | | | | | | | | | | | | | | | | | | | | | | | |  | | | | |
| real | | | | | 2.83 | | | | | .0047 | | | | | | | | imaginary | | | | | | | estimated **“SimplOr”** as more: | | | | | | | | | | | | | | | | | | | | | | | | | | | | | | | | | | | | | | | | | | | | |  | | | | |
| *clear* | | | *3.63* | | | | | | | *.0003* | | | | | | | | *blurred* | | | | | | | draining | | | | | 2.66 | | | | | | | | | | | | | .0078 | | | | | | | | | | | | stimulating | | | | | | | | | | | | | | |  | | | | |
| *regular* | | | *3.07* | | | | | | | *.0021* | | | | | | | | *irregular* | | | | | | | calms | | | | | 2.97 | | | | | | | | | | | | | .0029 | | | | | | | | | | | | arouses | | | | | | | | | | | | | | |  | | | | |
| *reliable* | | | *2.67* | | | | | | | *.0067* | | | | | | | | *unreliable* | | | | | | | severe | | | | | 3.16 | | | | | | | | | | | | | .0016 | | | | | | | | | | | | kind | | | | | | | | | | | | | | |  | | | | |
| active | | | | | | 3.88 | | | | | .0001 | | | | | | | passive | | | | | | | cold | | | | | | | | | 3.06 | | | | | | | | | | | | .0022 | | | | | | | | | | | warm | | | | | | | | | | | | | | | |  | |
| fast | | | | | | 3.81 | | | | | .0001 | | | | | | | slow | | | | | | | **insignific-t** | | | | | | | | | **3.71** | | | | | | | | | | | | **.0002** | | | | | | | | | | | **significant** | | | | | | | | | | | | | | | |  | |
| unrestrained | | | | | | 4.30 | | | | | .0000 | | | | | | | restrained | | | | | | | *irregular* | | | | | | | | | *3.09* | | | | | | | | | | | | *.0020* | | | | | | | | | | | *regular* | | | | | | | | | | | | | | | |  | |
| weaker Social endurance stronger | | | | | | | | | | | | | | | | | | | | | | | | | *irrational* | | | | | *2.91* | | | | | | | | | | | | | *.0036* | | | | | | | | | | | | | | | | *rational* | | | | | | | | | | |  | | | | |
| cold | | | | | | | 3.10 | | | | | | | .0019 | | | | | | | warm | | | | unstable | | | | | | | | | | | 3.31 | | | | | | | | | | | | | .0009 | | | | | | | | | | | | stable | | | | | | | | | | | | |  |
| **massive** | | | | | **3.20** | | | | | | | **.0014** | | | | | | | **delicate** | | | | | | weaker Intellectual endurance stronger | | | | | | | | | | | | | | | | | | | | | | | | | | | | | | | | | | | | | | | | | | | |  | | | | | |
| unknown | | | | | | | 3.61 | | | | | | | .0003 | | | | | | | known | | | | exciting | | | | | | | | | | | 3.49 | | | | | | | | | | | | | .0005 | | | | | | | | | | | | indifferent | | | | | | | | | | | | |  |
| atypical | | | | | | | 3.67 | | | | | | | .0002 | | | | | | | typical | | | | stimulating | | | | | | | | | | | 3.32 | | | | | | | | | | | | | .0009 | | | | | | | | | | | | draining | | | | | | | | | | | | |  |
| rare | | | | | | | 2.96 | | | | | | | .0031 | | | | | | | common | | | | kind | | | | | | | | | | | 2.82 | | | | | | | | | | | | | .0049 | | | | | | | | | | | | severe | | | | | | | | | | | | |  |
| imagined | | | | | | | 3.77 | | | | | | | .0002 | | | | | | | existent | | | | natural | | | | | | | | | | | 2.89 | | | | | | | | | | | | | .0039 | | | | | | | | | | | | artificial | | | | | | | | | | | | |  |
| **MEN of** | | | | | Z | | | | | | | *p*-level | | | | | | | | | |  | | | **MEN of** | | | | | | | | | | | | Z | | | | | *p*-level | | | | | | | | | | | | | | | |  | | | | | | | | | | |  | | | | | |
| weaker Social endurance stronger | | | | | | | | | | | | | | | | | | | | | | | | | lower Sensation Seeking higher | | | | | | | | | | | | | | | | | | | | | | | | | | | | | | | | | | | | | | | | | | | |  | | | | | |
| estimated **“SimplOr”** as more: | | | | | | | | | | | | | | | | | | | | | | | | | estimated **“SimplOr”** as more: | | | | | | | | | | | | | | | | | | | | | | | | | | | | | | | | | | | | | | | | | | | |  | | | | | |
| cold | | | | 3.10 | | | | | | | | | | | | .0019 | | | | | | | warm | | good | | | 2.98 | | | | | | | | | | | | | | | | | | | | | | | .0029 | | | | | | | | | | | | | bad | | | | | | | | | | |
| **massive** | | | | **3.20** | | | | | | | | | | | | **.0014** | | | | | | | **delicate** | | light | | | 3.03 | | | | | | | | | | | | | | | | | | | | | | | .0024 | | | | | | | | | | | | | dark | | | | | | | | | | |
| unknown | | | | 3.61 | | | | | | | | | | | | .0003 | | | | | | | known | | ordinary | | | 2.83 | | | | | | | | | | | | | | | | | | | | | | | .0047 | | | | | | | | | | | | | unusual | | | | | | | | | | |
| atypical | | | | 3.67 | | | | | | | | | | | | .0002 | | | | | | | typical | | known | | | 2.82 | | | | | | | | | | | | | | | | | | | | | | | .0048 | | | | | | | | | | | | | unknown | | | | | | | | | | |
| rare | | | | 2.96 | | | | | | | | | | | | .0031 | | | | | | | common | | imagined | | | 2.93 | | | | | | | | | | | | | | | | | | | | | | | .0034 | | | | | | | | | | | | | existent | | | | | | | | | | |
| imagined | | | | 3.77 | | | | | | | | | | | | .0002 | | | | | | | existent | | common | | | 2.98 | | | | | | | | | | | | | | | | | | | | | | | .0029 | | | | | | | | | | | | | rare | | | | | | | | | | |
| lower Sens. to Probabilities higher | | | | | | | | | | | | | | | | | | | | | | | | | *reliable* | | | *2.90* | | | | | | | | | | | | | | | | | | | | | | | *.0037* | | | | | | | | | | | | | *unreliable* | | | | | | | | | | |
| pleasant | | | | 2.74 | | | | | | | | | | | | .0062 | | | | | | | irritating | | *obvious* | | | *3.01* | | | | | | | | | | | | | | | | | | | | | | | *.0026* | | | | | | | | | | | | | *obscure* | | | | | | | | | | |
| good | | | | 2.80 | | | | | | | | | | | | .0051 | | | | | | | bad | | solid | | | 3.14 | | | | | | | | | | | | | | | | | | | | | | | .0017 | | | | | | | | | | | | | fragile | | | | | | | | | | |
| **smooth** | | | | **2.68** | | | | | | | | | | | | **.0074** | | | | | | | **rough** | | steady | | | 3.72 | | | | | | | | | | | | | | | | | | | | | | | .0002 | | | | | | | | | | | | | faltering | | | | | | | | | | |
| true | | | | 3.09 | | | | | | | | | | | | .0020 | | | | | | | false | | stable | | | 2.95 | | | | | | | | | | | | | | | | | | | | | | | .0031 | | | | | | | | | | | | | unstable | | | | | | | | | | |
| *rational* | | | | *4.07* | | | | | | | | | | | | *.0000* | | | | | | | *irrational* | |  | | |  | | | | | | | | | | | | | | | | | | | | | | |  | | | | | | | | | | | | |  | | | | | | | | | | |
| stable | | | | 2.82 | | | | | | | | | | | | .0048 | | | | | | | unstable | |  | | |  | | | | | | | | | | | | | | | | | | | | | | |  | | | | | | | | | | | | |  | | | | | | | | | | |
