# Supplementary material for: Observer Bias: An Interaction of Temperament Traits with Biases in the Semantic Perception of Lexical Material
Source: PLoS One. 2014 Jan 27;9(1):e85677. doi: 10.1371/journal.pone.0085677 (PMC3903487; doi:10.1371/journal.pone.0085677)
Supplement: Table S3 — Significant sex differences in estimations of selected temperament groups. The font alternates between the scales of the Stimulation factor (underlined), Evaluation (normal), Power (bold), Complexity (bold italic), Reality-Probability (normal), Organization (italic) and Stability-Limitation (normal). “Work” concepts: Work, Effort, Task, Activity. (DOC) [file pone.0085677.s004.doc]

**Supporting Information, Table S3.** Significant SEX differences in estimations of selected temperament groups. The font alternates between the scales of the Stimulation factor (underlined), Evaluation (normal), **Power** (**bold**), Complexity (***bold italic***), Reality-Probability (normal), *Organization* (*italic*) and Stability-Limitation (normal). “Work” concepts: Work, Effort, Task, Activity.

| **Study 1** | | | Z | | *p*-level | | |  | **Study 1** | | Z | *p*-level | |  | | | |  |
| --- | --- | --- | --- | --- | --- | --- | --- | --- | --- | --- | --- | --- | --- | --- | --- | --- | --- | --- |
| Men with **Low Motor Endurance** Women | | | | | | | | | Men with **High Motor Endurance** Women | | | | | | | | |  |
| estimated **“Work”** as more: | | | | | | | | | estimated **“Work”** as more: | | | | | | | | |  |
| pale | | | 3.10 | | .0019 | bright | | | trivial | 3.31 | | .0009 | original | | | | |  |
| uninteresting | | | 3.67 | | .0002 | interesting | | | ***simple*** | ***3.71*** | | ***.0002*** | ***complex*** | | | | |  |
| decline | | | 3.90 | | .0001 | progress | | | rare | 2.78 | | .0054 | common | | | | |  |
| dangerous | | 3.56 | | .0004 | | | safe | | Men with **High Social Endurance** Women | | | | | | | | |  |
| ***discrete*** | | | ***3.90*** | | ***.0001*** | ***continuous*** | | | trivial | 3.86 | | .0001 | original | | | | |  |
| ***one-dimen*** | | | ***5.32*** | | ***.0000*** | ***multi-dimen*** | | | **insignificant** | **3.14** | | **.0017** | **significant** | | | | |  |
| ***uniform*** | | | ***3.48*** | | ***.0005*** | ***diverse*** | | | ***simple*** | ***2.84*** | | ***.0044*** | ***complex*** | | | | |  |
| ***simple*** | | | ***2.98*** | | ***.0028*** | ***complex*** | | | imagined | 3.33 | | .0009 | existent | | | | |  |
| ***artificial*** | ***2.98*** | | | | ***.0029*** | ***natural*** | | | rare | 2.96 | | .0031 | common | |  | | | |
| imaginary | 3.63 | | | | .0003 | real | | | *unorganized* | *3.20* | | *.0014* | *organized* | |  | | | |
| impossible | | | 4.47 | | .0000 | possible | | | Men with **High Social Tempo**  Women | | | | | | | | |  |
| rare | 2.98 | | | | .0029 | common | | | calms | 2.95 | | .0032 | arouses | | | | | |
| inexplicable | | | 3.09 | | .0020 | understand | | | ***discrete*** | ***3.31*** | | ***.0009*** | ***continuous*** | | |  | | |
| unusual | | | 3.22 | | .0013 | ordinary | | | ***simple*** | ***2.89*** | | ***.0039*** | ***complex*** | | |  | | |
| *blurred* | | | *3.20* | | *.0014* | *clear* | | | rare | 4.26 | | .0000 | common | | |  | | |
| *senseless* | | | *4.25* | | *.0000* | *justified* | | | unusual | 3.31 | | .0009 | ordinary | | |  | | |
| *unreliable* | | | *2.91* | | *.0036* | *reliable* | | | irreplaceable | 2.99 | | .0028 | replaceable | | |  | | |
| *imprecise* | | | *2.72* | | *.0066* | *precise* | | | impossible | 3.23 | | .0013 | possible | | |  | | |
| faltering | | | 4.11 | | .0000 | steady | | | finite | 2.98 | | .0029 | infinite | | |  | | |
| *unorganized* | | | *4.67* | | *.0000* | *organized* | | | Men with **Low Social Endurance** Women | | | | | | | | |  |
| slow | | | 3.13 | | .0018 | fast | | | pale | 3.29 | | .0010 | bright | | |  | | |
| Men with **Low Social Tempo** Women | | | | | | | | | ***one-dimen*** | ***3.31*** | | ***.0009*** | | ***multi-dim*** | | |  | |
| rough | | | 3.48 | | .0005 | smooth | | | ***uniform*** | ***4.26*** | | ***.0000*** | | ***diverse*** | | |  | |
| **insignificant** | | | **3.42** | | **.0006** | **significant** | | | ***simple*** | ***3.71*** | | ***.0002*** | | ***complex*** | | |  | |
| ***uniform*** | | | ***3.04*** | | ***.0024*** | ***diverse*** | | | ***artificial*** | ***3.00*** | | ***.0027*** | | ***natural*** | | |  | |
| imagined | | | 2.76 | | .0058 | existent | | | impossible | 3.28 | | .0010 | | possible | | |  | |
|  | | |  | |  |  | | | rare | 3.02 | | .0025 | | common | | |  | |
|  | | |  | |  |  | | | inexplicable | 3.01 | | .0026 | | understan | | |  | |
|  | | |  | |  |  | | | slow | 3.97 | | .0001 | | fast | | |  | |
|  | | |  | |  |  | | | finite | 3.15 | | .0016 | | infinite | | |  | |
| **Study 2** | | | Z | | *p*-level | | |  | **Study 2** | | Z | *p*-level | |  | | |  | |
| Men with **High Social Endurance** Women | | | | | | | | | Men with **High Social Tempo** Women | | | | | | | |  | |
| estimated **“Work”** as more: | | | | | | | | | estimated **“Work”** as more: | | | | | | | |  | |
| trivial | | | 3.86 | | .0001 | original | | | calms | 2.95 | | .0032 | | arouses | | |  | |
| **insignificant** | | | **3.14** | | **.0017** | **significant** | | | ***discrete*** | ***3.31*** | | ***.0009*** | | ***continuo.*** | | |  | |
| ***simple*** | | | ***2.84*** | | ***.0044*** | ***complex*** | | | ***simple*** | ***2.89*** | | ***.0039*** | | ***complex*** | | |  | |
| imagined | | | 3.33 | | .0009 | existent | | | impossible | 3.23 | | .0013 | | possible | | |  | |
| rare | | | 2.96 | | .0031 | common | | | rare | 4.26 | | .0000 | | common | | |  | |
| *unorganized* | | | *3.20* | | *.0014* | *organized* | | | irreplaceable | 2.99 | | .0028 | | replaceabl | | |  | |
|  | | |  | |  |  | | | unusual | 3.31 | | .0009 | | ordinary | | |  | |
|  | | |  | |  |  | | | finite | 2.98 | | .0029 | | infinite | | |  | |
| **Study 1** | | | Z | | *p*-level | | |  | **Study 1** | | Z | *p*-level | |  | | |  | |
| Men with **Low Social Endurance** Women | | | | | | | | | Men with **High Social Tempo** Women | | | | | | | |  | |
| estimated **Reality, Life, Present** as more: | | | | | | | | | estimated **Reality, Life, Present** as more: | | | | | | | |  | |
| indifferent | | | 3.10 | | .0019 | exciting | | | ***discrete*** | ***3.24*** | | ***.0012*** | | ***continuo.*** | | |  | |
| calms | | | 2.96 | | .0031 | arouses | | | ***ordered*** | ***2.85*** | | ***.0044*** | | ***chaotic*** | | |  | |
| **weak** | | | **2.95** | | **.0032** | **powerful** | | | ***simple*** | ***3.10*** | | ***.0020*** | | ***complex*** | | |  | |
| ***easy*** | | | ***2.74*** | | ***.0062*** | ***difficult*** | | | ***replaceable*** | ***2.66*** | | ***.0077*** | | ***irreplace.*** | | |  | |
| imagined | | | 2.72 | | .0066 | existent | | | ***easy*** | ***3.66*** | | ***.0003*** | | ***difficult*** | | |  | |
| improbable | | | 2.71 | | .0068 | inevitable | | | imagined | 3.21 | | .0013 | | existent | | |  | |
|  | | |  | |  |  | | | imaginary | 3.41 | | .0006 | | real | | |  | |
|  | | |  | |  |  | | | impossible | 2.82 | | .0048 | | possible | | |  | |
|  | | |  | |  |  | | | rare | 3.03 | | .0025 | | common | | |  | |
