# Supplementary material for: Observer Bias: An Interaction of Temperament Traits with Biases in the Semantic Perception of Lexical Material
Source: PLoS One. 2014 Jan 27;9(1):e85677. doi: 10.1371/journal.pone.0085677 (PMC3903487; doi:10.1371/journal.pone.0085677)
Supplement: Text S1 — Validation summary of the Structure of Temperament Questionnaire. (DOC) [file pone.0085677.s005.doc]

**Supporting Information 1 (Text S1). Validation summary of the Structure of Temperament Questionnaire**

During the experimental validation of the STQ in the 1980-90s, the performance of subjects on the following measures were compared with STQ scales in a series of studies: speed of writing, reading and generation of words, maximal and optimal tempo of performance in sensory-motor tasks and intellectual (including unsolvable) tasks, performance on non-verbal tasks with which subjects were unfamiliar, rigidity of perception in tactile and visual modalities, duration of the switch from one way of solving the task to another, mobility in attention, variability in line drawing (Rusalov, 1979, 1989, Rusalov & Trofimova, 2007). The first version of STQ had 8 scales: the four temperament scales (ergonicity, plasticity, tempo and emotionality) assessed in social and physical activity. Each scale had 12 items (Rusalov, 1989). The Extended version included additional 4 scales related to intellectual activity. In the studies of the concurrent validity of the initial STQ it was compared to Eysenck’s EPQ (Brebner & Stough, 1993; Rusalov, 1989; Zinko, 2006), NEO-FFI (Bodunov et al., 1996, Dumenci, 1995), Strelau’s PTS (Bodunov et al., 1996; Ruch et al., 1991; Strelau, 1999, Trofimova, 2009), meaning attribution to neutral objects (Trofimova, 1999), the Motivation for Achievement scale (Vorobieva, 2004), adaptivity strategies in the Dembo-Hoppe Level of Aspiration experiment (Zin’ko, 2006), 25 measures of Mobility (Rathee & Singh, 2001), Dissociative Experiences Scale (Beere & Pica, 1995, Vasyura, 2008). References to STQ validation with the Rogers Adaptivity scale, the Torrance’s Nonverbal Tests of Creative Thinking, Rotter’s Locus of Control scale, a choice of profession, with other 8 measures of plasticity, STAI, MAS, Wechsler, Shepard and Gotshield Figure tests, Rosenzveig test, Cattell’s 16-PF inventory, and with the school grades of high-school students, can be found in the work of Rusalov and Trofimova (2007).

The administration of the English version of the STQ (STQ-E) to American, Australian and Canadian samples demonstrated that it has a factor structure similar to that of the Russian language version, and that it has good reliability and internal consistency (Bishop Jacks & Tandy, 1993; Bishop & Hertenstein, 2004; Dumenci, 1995, 1996 (initial version of the STQ); Rusalov, 1997, 2004; Stough et al., 1991; Rusalov and Trofimova, 2007; Trofimova, 2010b). Chinese (STQ-C), Urdu (STQ-U) and Polish (STQ-P) Extended versions of the STQ, administered among corresponding populations, showed reliability coefficients in the range .70-.86, item-total correlations in the range .42-.73, and all versions demonstrated robust factor structures similar to those of the original version (Trofimova, 2010b).

The Confirmatory Factor Analysis of the Compact STQ (STQ-77) using data from a Canadian sample shows a satisfactory fit of the traditional 4-factor STQ activity-specific model, grouping the scales to the factors of Motor, Social, Intellectual activity and Emotionality and having 2 correlated residuals (from the new scale of Sensitivity to Sensations to Impulsivity and Neuroticism scales) with the CFI > .90, RMSEA < .07 and RMSR < .06 (Trofimova, 2010a). The studies of reliability and content, concurrent and discriminant validity of STQ-77 scales showed that the reliability of these scales is in the range of .70-.86. The time of testing correlated statistically significantly with the Social tempo scale (*r* = -.31) (Trofimova, 2010c), and the time of performance on a task involving the classification of common words also had the most significant negative correlations with Social tempo, as well as with Self-Confidence scale of STQ-77 (-.36 and -.29, respectively) (Trofimova, 2010c). High school grades show activity-specific correlations with the STQ-77 scales (Trofimova & Sulis, 2011): the grades in athletics correlate with the STQ-77 scales of Motor Ergonicity and tempo (*r* = .53 and .45), the grades in verbal assignments have *r* = .28 with Social Ergonicity, and *r* = .27 with Social tempo scale, and the grades in math and science correlate only with the scales of Intellectual Ergonicity and Plasticity (*r* = .26 and .22, respectively) (Trofimova and Sulis, 2009). Significant positive correlations are found between the new STQ-77 scales of Impulsivity, Sensitivity to Sensations, Empathy and the corresponding scales of Impulsiveness (*r* = .51), Venturesomeness (.64) and Empathy (.73) of Eysenck’s I-7 questionnaire (Trofimova & Sulis, 2011). STQ-77 Sensitivity to Sensation scale shows good agreement with Impulsivity scale (*r* =.68) and Zuckerman Sensation Seeking Scale (*r* =.37) (Trofimova, 2010c). Clinical symptoms of anxiety and comorbid symptoms of anxiety and depression correlated with high Impulsivity, low Plasticity, low Motor Tempo and Endurance as measured by the STQ-77 (Trofimova & Sulis, 2010).

Extraversion, as measured by the Big Five (NEO-FFI), correlates with Social Ergonicity and Impulsivity scales of the STQ-77 with *r* = .46 and .52, respectively, and Neuroticism scales of NEO-FFI and STQ-77 correlate with *r* = .38, and all these values show large effect sizes (Trofimova, 2010a). Openness to Experience of Big Five correlate significantly with the STQ-77 scales of Intellectual Ergonicity (*r* =.31), Sensitivity to Probabilities (*r* =.40), Impulsivity (*r* =.25) and Empathy (*r* =.52); the scale of Agreeableness of Big Five correlate significantly with the Empathy scale of the STQ-77 (*r* =.46), and the scale of Conscientiousness of Big Five has the most significant correlation with the STQ-77 scales of ERM (*r* =.35) and ERI (*r* =.34).

*References for the Attachment A*

Beere D, Pica M (1995) The predisposition to dissociate: the temperamental traits of flexibility/rigidity daily rhythm emotionality and interactional speed. Dissociation 8/4: 236-240

Bishop D, Jacks H, Tandy S (1993) Structure of Temperament Questionnaire (STQ): Results from a US sample. Pers Ind Diff 14(3): 485-487

Bishop D, Hertenstrein M (2004) A confirmatory factor analysis of Structure of Temperament Questionnaire. Edu Psychol Meas 64/6: 1019-1029

Bodunov MV, Bezdenezhnykh BN, Alexandrov YI (1996) Peculiarities of psychodiagnostic test item responses and the structure of individual experience Psychol J (Psikhologicheskiy Zhurnal) 17(4): 87-96

Dumenci L (1996) Factorial validity of scores on the Structure of Temperament Questionnaire. Edu Psychol Meas 56(3): 487-493 US: Sage Publications

Rathee N, Singh R (2001) Mobility or/and Lability of the Nervous Processes as Temperamental Trait(s). Pers Indiv Diff 31: 1091-1104

Ruch W Angleitner A , Strelau J (1991) The Strelau Temperament Inventory – Revised (STI-R): Validity studies. Eur J Pers 5: 287-308

Rusalov VM (2004) Formal-dynamical properties of individual (Temperament) Short theory and methods of measurement for various age groups Moscow: Russian Academy of sciences IPAN (In Russian)

Rusalov VM, Trofimova IN (2007) Structure of temperament and its measurement PSP: Psychological Services Press Toronto

Stough C, Brebner J , Cooper C (1991) The Rusalov Structure of Temperament Questionnaire (STQ): results from an Australian sample. Pers Indiv Diff 12: 1355-1357

Trofimova I (2009) Exploration of the benefits of an activity-specific test of temperament. Psychol Rep 105: 643-658

Trofimova I (2010a) An investigation into differences between the structure of temperament and the structure of personality. Am J Psychol 123/4: 467-480

Trofimova I (2010b) Exploration of the activity-specific model of temperament in four cultures. Inter J Psychol Psychol Ther 10/1: 79-95

Trofimova I (2010c) Questioning the “general arousal” models Open Beh Sci Psychol, 4: 1-8

Trofimova I, Sulis W (2010) An investigation of temperament in adults with comorbid depression and anxiety. Adv Biosci Biotech 1/3: 190-199, DOI: 104236/abb201013027

Trofimova I, Sulis W (2011) Is temperament activity-specific? Validation of the Structure of Temperament Questionnaire – Compact (STQ-77). Inter J Psychol Psychol Ther 11/4: 167-180

Vasyura SA (2008) Psychology of male and female communicative activity. Span J Psychol 11/1: 289-300

Vorobieva EV (2004) Modern psychogenetic studies of intelligence and theory of motivation for achievements. J Appl Psychol 3: 53-59

Zin’ko EV (2006) Characteristics of self-image and of level of aspiration and its parameters. Psychol J (Psikhologicheskii Zjurnal) 3: 18-30; 4: 15-25
